# The application of random forest-based models in prognostication of gastrointestinal tract malignancies: a systematic review

**DOI:** 10.3389/frai.2025.1517670

**Published:** 2025-07-18

**Authors:** Zhina Mohamadi, Ahmad Shafizadeh, Yasaman Aliyan, Seyedeh Fatemeh Shayesteh, Parsa Goudarzi, Alireza Khodabandeh, Amirali Vaghari, Helma Ashrafi, Omid Bahrami, Armin ZarinKhat, Yalda Khodabandeh, Kimia Pouyan

**Affiliations:** ^1^Faculty of Medicine, Kermanshah University of Medical Sciences, Kermanshah, Iran; ^2^Faculty of Medicine, Tehran Medical Sciences, Islamic Azad University, Tehran, Iran; ^3^Faculty of Medicine, Tehran Medical Science Islamic Azad University, Tehran, Iran; ^4^Faculty of Allied Medicine, Tehran University of Medical Sciences, Tehran, Iran; ^5^Faculty of Medicine, Shahid Beheshti University of Medical Sciences, Tehran, Iran; ^6^Student Research Committee, Qazvin University of Medical Sciences, Qazvin, Iran; ^7^Faculty of Medicine, Shahroud University of Medical Sciences, Semnan, Iran; ^8^School of Medicine, Tehran University of Medical Sciences, Tehran, Iran; ^9^Faculty of Medicine, Guilan University of Medical Sciences, Rasht, Iran; ^10^Faculty of Medicine, Ahvaz University of Medical Sciences, Ahvaz, Iran

**Keywords:** random forest, prognostication, GI tract cancers, malignancy, prognose

## Abstract

**Introduction:**

Malignancies of the GI tract account for one-third of cancer-related deaths globally and more than 25% of all cancer diagnoses. The rising prevalence of GI tract malignancies and the shortcomings of existing treatment approaches highlight the need for better predictive prediction models. RF’s machine-learning method can predict cancers by using numerous decision trees to locate, classify, and forecast data. This systematic study aims to assess how well RF models predict the prognosis of GI tract malignancies.

**Methods:**

Following PRISMA criteria, we performed a systematic search in PubMed, Scopus, Google Scholar, and Web of Science until May 28, 2024. Studies used RF models to forecast the prognosis of GI tract malignancies, including esophageal, gastric, and colorectal cancers. The QUIPS approach was used to evaluate the quality of the included studies.

**Results:**

Out of 1846 records, 86 studies met inclusion requirements; eight were disqualified. Numerous studies showed that when combining clinical, genetic, and pathological data, RF models were very accurate and dependable in predicting the prognosis of GI tract malignancies, responses, recurrence, survival rates, and metastatic risks, distinguishing between operable and inoperable tumors, and patient outcomes. RF models outperformed conventional prognostic techniques in terms of accuracy; several research studies reported prediction accuracies of over 80% in survival rate estimates.

**Conclusion:**

RF models, in terms of accuracy, performed better than the conventional approaches and provided better capabilities for clinical decision-making. Such models can increase the life quality and survival of patients by personalizing their treatment regimens for cancers of the GI tract. These models can, in a significant manner, raise patients’ survival and quality of life through hastening clinical decision-making and providing personalized treatment options.

## Introduction

Over 25% of all cancer cases worldwide and about one-third of cancer-related deaths are caused by gastrointestinal (GI) tract malignancies, which are a result of major lifestyle changes brought on by socioeconomic growth, increased consumption of processed foods, and higher rates of alcohol and tobacco use (Huang et al., 2023). By 2040, GI cancer mortality is expected to increase by 58 and 73%, to 7.5 million and 5.6 million cases, respectively, due to demographic shifts and global population expansion (Ferlay et al., 2021).

Despite the potential of novel therapeutic techniques like immunotherapy, their effectiveness varies according to the patient’s demographics (Wang et al., 2023). Patients with high PD-L1 expression or microsatellite instability-high (MSI-H) usually experience poor outcomes from immune checkpoint drugs targeting PD-1 and PD-L1 inhibitors (Shen and Zhao, 2018). Especially in the cases of patients receiving immunotherapy, conventional prognostic factors, including clinical staging and tumor markers, sometimes may not be able to precisely predict the treatment outcomes (Zhou et al., 2024). The clinical variability of GI malignancies, which arise from a range of genetic, pathological, and clinical factors, complicates the establishment of conventional therapeutic approaches (Chen et al., 2022).

Esophageal cancer (EC) is the eighth most common and sixth most lethal gastrointestinal disease worldwide, with a mere 20% five-year survival rate (Siegel et al., 2022; Uhlenhopp et al., 2020; Kasai et al., 2024). Despite improvements in early identification and treatment, gastric cancer (GC) continues to rank third in the world for cancer-related fatalities (Yu et al., 2024; Bray et al., 2018). Colorectal cancer (CRC) is the second most common cause of cancer-related death in the United States and one of the most common cancers in Western countries (Du et al., 2022; Van Cutsem et al., 2014).

Most of the models now used for the clinical assessment of GI malignancies are based on conventional statistical methods. However, these techniques frequently fail to analyze the high-dimensional and complicated data inherent in cancer prediction. Because machine learning (ML) techniques, in particular Random Forest (RF), can handle diverse datasets, manage missing values, and capture nonlinear relationships between variables, they provide a tempting alternative (Breiman, 2001; An et al., 2022).

RF is a powerful ensemble learning method that performs better than other machine learning methods such as support vector machines (SVM) and neural networks. It is resistant to missing data, scalable, and interpretable—all of which are typical issues in clinical datasets (Song et al., 2022). Despite their strength, neural networks may need extensive data preparation and are prone to overfitting. However, SVM is less interpretable in a therapeutic setting and has trouble with big datasets (Książek et al., 2019). RF is particularly well-suited for prognostic modeling in GI malignancies because of its ability to integrate several data sources, identify important components, and produce intelligible forecasts (Parmar et al., 2015; Tang and Ishwaran, 2017; Bennett and Campbell, 2000). The concepts of personalized medicine align with RF’s shown capacity to evaluate intricate clinical datasets and its resilience in handling missing data (Kaur et al., 2024).

Although the majority of recent research has focused on machine learning approaches in general, few studies have focused on RF’s unique advantages in addressing the unique challenges of GI malignancies, including clinical heterogeneity and missing data (Abbas et al., 2024; Christou and Tsoulfas, 2021). By filling up these gaps, the current study investigates how well RF models could predict clinical outcomes for patients with gastrointestinal cancer as compared to conventional statistical methods.

To improve the predicted accuracy of RF models, future research should evaluate them using bigger and more varied datasets. There should be efforts to incorporate these models into routine clinical decision-making due to their effectiveness in handling missing data and identifying key factors. RF models can also support personalized treatment by integrating various patient data (Rizinde et al., 2023). Addressing implementation challenges, like data preprocessing and clinician training, is essential for practical use. Bioinformaticians, data scientists, and oncologists must collaborate to standardize these methods (Asif et al., 2023). Finally, research must examine the broader use of RF models in other cancer types. The results of the review study demonstrate the propensity of RF algorithms to guide future machine learning research and enhance clinical decision-making in patient care and treatment planning by accurately predicting outcomes for various GI cancer types.

## Methods

This investigation was conducted in compliance with the Preferred Reporting Items for Systematic Reviews and Meta-Analyses (PRISMA2020) criteria to enhance the coordination and design of systematic reviews (Page et al., 2021). The registration DOI for our Open Science Framework (OSF) systematic review is DOI 10.17605/OSF. IO/X25ZN (OSF, 2025).

### Information sources and search strategy

We developed a thorough search approach to find research using RF-based models for GI cancer prognostication. A comprehensive and detailed search of several databases, from each database’s inception to May 28, 2024, was conducted. The databases included PubMed/MEDLINE, Scopus, Google Scholar, and Web of Science. The results shown in Table 1 show that a controlled vocabulary along with keywords from each database was used to find the use of RF-based models for the prediction of GI tract cancers.

**Table 1:** Search strategy and article retrieval process for the application of random forest-based (RF) models in prognostication of gastrointestinal tract malignancies.

Database	Search strategy	Results
PubMed/MEDLINE	(“random forest”[Title/Abstract] OR “RF”[Title/Abstract]) AND (“Prognosis”[Title/Abstract] OR “prognoses”[Title/Abstract] OR “prognostication”[Title/Abstract] OR “prediction”[Title/Abstract] OR “Prognostic”[Title/Abstract]) AND (“gastrointestinal cancer”[Title/Abstract] OR “gastrointestinal cancers”[Title/Abstract] OR “colonic neoplasm”[Title/Abstract] OR “esophageal cancer”[Title/Abstract] OR “stomach cancer”[Title/Abstract] OR “gastric cancer”[Title/Abstract] OR “intestinal cancer”[Title/Abstract] OR “colorectal cancer”[Title/Abstract] OR “rectal cancer”[Title/Abstract] OR “anal cancer”[Title/Abstract] OR “small bowel cancer”[Title/Abstract] OR “duodenal cancer”[Title/Abstract])	447
Web of science	(TS = (Random forest) OR TS = (RF)) AND (TS = (“Gastrointestinal cancers”) OR TS = (“Esophageal Neoplasms”) OR TS = (“Stomach Neoplasm”) OR TS = (“Rectal Neoplasms”) OR TS = (“Gastrointestinal Neoplasms”) OR TS = (“Gastric Cancer”) OR TS = (“Colon Cancer”) OR TS = (“Anal Cancer”) OR TS = (“small bowel cancer”) OR TS = (“duodenal cancer”)) AND (TS = (prognostication) OR TS = (prognosis) OR TS = (prediction))	646
Scopus	(TITLE-ABS-KEY (“Random forest”) OR TITLE-ABS-KEY(RF)) AND (TITLE-ABS-KEY (“Gastrointestinal cancers”) OR TITLE-ABS-KEY (“Esophageal Cancer”) OR TITLE-ABS-KEY (“Stomach Cancer”) OR TITLE-ABS-KEY (“Intestinal Cancer”) OR TITLE-ABS-KEY (“Colon Cancer”) OR TITLE-ABS-KEY (“Rectal Cancer”) OR TITLE-ABS-KEY (“Anal Cancer”)) AND (TITLE-ABS-KEY(prognostication) OR TITLE-ABS-KEY(prognosis) OR TITLE-ABS-KEY(prediction))	839
Google scholar	allintitle: random forest “gastric cancer” OR “esophageal cancer” OR “colorectal cancer” OR “colonic cancer” OR “anal cancer” OR “rectal cancer” OR “gastrointestinal cancer”	40
Total	Articles were identified from four electronic databases	1932
Duplicate removal	Duplicates identified and excluded	1,491
Abstract and title screening	Articles were excluded after screening abstracts and titles	1,846
Inclusion for analysis	Articles with accessible full texts that met eligibility criteria were included for full review and data extraction.	86
Final articles	8 articles were excluded due to lack of full text	78

### Data screening and eligibility criteria

We used RAYYAN.ai to assess the search results (Ouzzani et al., 2016). Using a variety of artificial intelligence tools, this platform facilitates the screening and decision-making procedures in systematic reviews. Five reviewers (Zh. M., Y. A., A. S., F. Sh., and M. A.) evaluated the abstracts and titles of the papers found using our search approach impartially and independently. After the two reviewers had resolved their disagreements through a consensus-building process, a third independent reviewer (A. ZKH) verified the final choices. The RAYYAN platform helped with the process by eliminating unnecessary information from the search results and resolving any overlaps or discrepancies in reviewer scores.

To find research that used RF-based models to create or validate prognostic models for GI malignancies, the inclusion criteria were specially created. This supports our goal of assessing RF’s performance and application in this situation. This investigation includes every original English-language study that used RF to develop a model for predicting the prognosis of a particular kind of GI cancer (Table 2). The inclusion criteria were only looked at the level of the article titles and abstracts during the first screening stage. However, we checked the contained articles’ entire texts in the next step. Articles where the whole text was unavailable or where the usage of RF was not required for model creation were not included in the review.

**Table 2:** Step-by-step checklist to include an article.

Inclusion checklist
1. A Journal article
2. An English language article
3. A GI tract malignancy
4. Developing an outcome predictor model
5. Utilizing RF to build the model
6. Essential application of RF in model development*
7. Article full text available

*The essential application of RF was determined by the use of RF in the method section of the article and by comparing the results of the model with other algorithm-based models.

### Data extraction and quality assessment

The retrieved variables were selected to guarantee a thorough assessment of RF’s usefulness in prognostic modeling for GI malignancies. Using the full texts of the included articles, we extracted the following information to align with our research objectives: Title, Author, Year, Nation, Study Objective, Population, pathology kind, RF’s use in the model, development or validation of a model, Validation type, prognostication factor or factors, method, result, conclusion, and evidence quality (Table 3).

**Table 3:** Esophageal cancer (EC).

References	Dataset	Task	Approaches	Disease type	Performance (metric)	Results
Rahman et al. (2020)	Clinical and imaging data (post-surgery)	Recurrence prediction	Random Forest (RF), Elastic Net Regularized Logistic Regression, XGBoost, hyperparameter tuning	Adenocarcinoma	High predictive performance (AUC), especially for high-risk recurrence cases	Successfully identified high-risk recurrence patients with optimized hyperparameters
Ou et al. (2019)	Radiomic features extracted from CT scans	Tumor classification (respectable vs. non-respectable)	Random Forest (RF)	Squamous Cell Carcinoma	High accuracy (AUC outperformed other ML models)	RF proved superior in differentiating tumor resectability using radiomic features
Paul et al. (2017)	Clinical and imaging data	Prediction of treatment outcomes and patient survival	Random Forest (RF) combined with Genetic Algorithm	Locally Advanced EC	High AUC, minimal misclassification error	Key imaging and clinical features identified for predicting outcomes with low error rates

The models that were previously used in studies on GI cancer were considered validation models. The process of verifying the applicability of a model using a population different from the sample population described in the data extraction phase was referred to as external validation in this context. While external validation findings are meant to be generalizable to the reference population, internal validation focuses mainly on the results’ robustness when applied to the training dataset (Altman and Royston, 2000). Using the QUIPS tool, four assessors (F. Sh, A. Kh, Y. A., P. G., and M. A.) independently assessed each article to determine the risk of bias and the quality of the included studies (Hayden et al., 2006). The instrument consists of six distinct domains: study participation, study confounding, study attrition, outcome measurement, prognostic factor measurement, statistical analysis, and reporting. For each domain, a set of three to seven prompting items is utilized. Studies evaluated as high quality in these domains were prioritized in the synthesis of data to guarantee strong conclusions.

Due to observation of considerable heterogeneity in RF model implementation across studies, instead of conducting a meta-analysis, a narrative and systematic approach, highlighting trends in predictive performance for each cancer type, was chosen.

## Results

### Study selection

**Figure 1:**
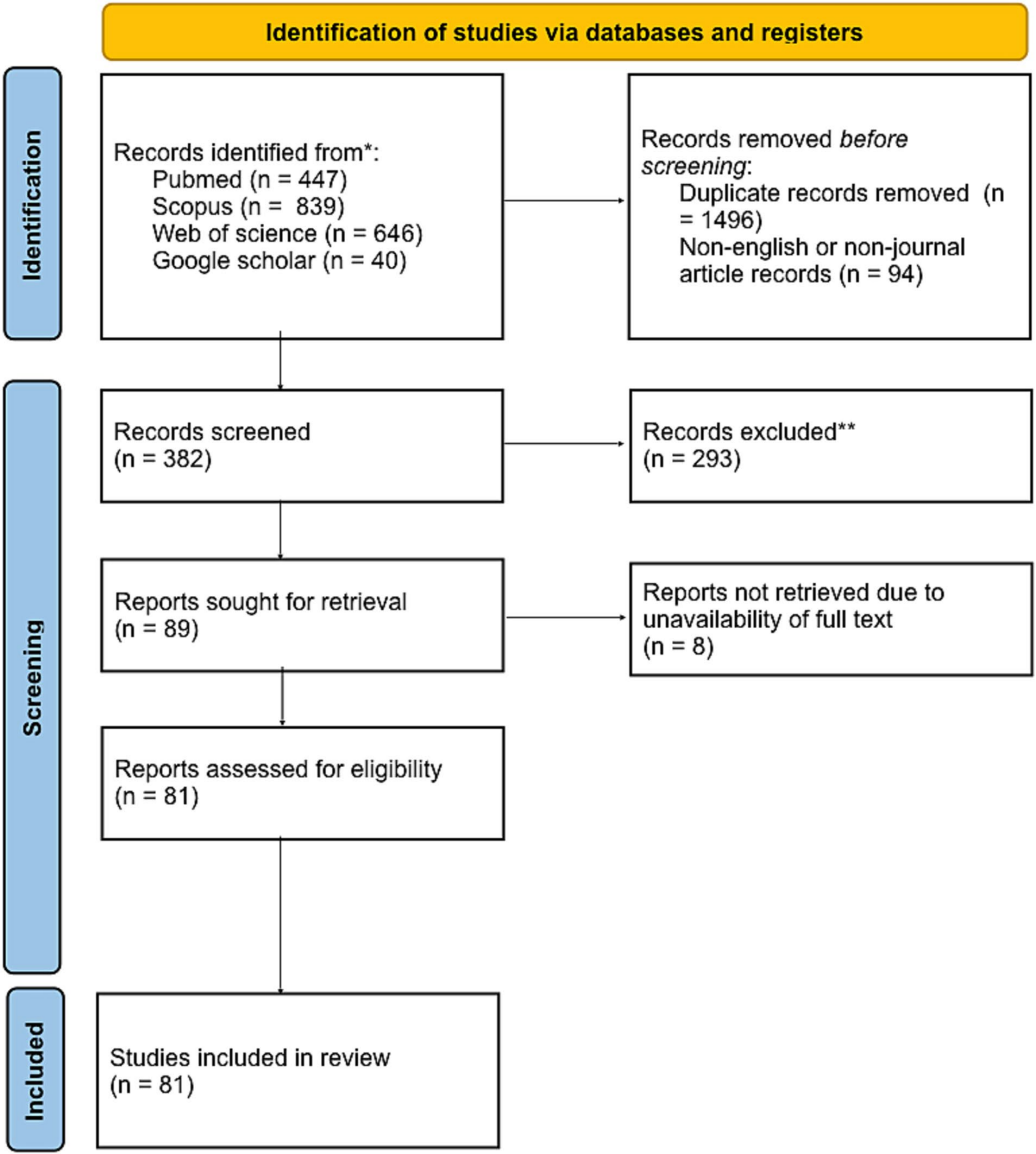
PRISMA 2020 flow diagram for new systematic reviews which included searches of databases.

A systematic exploration of four electronic databases identified 1,932 articles. After removing 1,846 items, 1,491 were duplicates. The remaining articles were split into 86 accessible and 8 non-accessible (Figure 1). We extracted data from our 86 full-text articles using 16 factors (author’s name, title, year of publication, country, aim of a study, population studied, type of pathology, use of RF in model, model development or validation, type of validation, model used for validation, factors used for prognostication, method, outcome, conclusion, quality of the evidence).

### Study characteristics

The reviewed papers span the years 2011 through 2024. Fewer studies came from North America, Europe, and the Middle East, with the majority of the research being done in Asia, particularly China. Studies that are cross-sectional and retrospective make up the majority of the literature. The majority of the papers (33 and 36, respectively) showed moderate and low risk of bias, according to the risk of bias evaluation, while only a small subgroup (Ferlay et al., 2021) was found to have a high risk of bias. 27 of the 86 included studies briefly noted their approach to handle missing data.

There were 5 different cancers mentioned in the 86 articles found in full text, which are categorized by the number of articles they are mentioned in and the type of validation used for them. An abstract of the methods used in the articles with the highest quality of evidence for each of the 5 cancers is gathered and tabled along with the other mentioned information (Table 4).

**Table 4:** Gastric cancer (GC).

References	Dataset	Task	Approaches	Disease type	Performance (metric)	Results
Tian et al. (2023) and Li et al. (2018)	Radiomic and clinical-pathological data	Prediction of metastases, serosal invasion, survival	Random Forest (RF), Support Vector Machines (SVM), Artificial Neural Networks (ANN)	Advanced Gastric Cancer	High AUC, RF models outperformed others in many datasets	RF consistently outperformed other ML models, achieving reliable survival and metastasis predictions
Huang et al. (2021) and Liu et al. (2021)	Clinical and radiomic data	Overall survival prediction	RF ensembles and feature selection methods	Serosal invasion/metastases	High predictive accuracy, consistently robust AUC values	RF provided insights into critical radiomic and clinical predictors affecting survival

### Findings

86 eligible articles were identified, of which 50 presented model development, 46 presented only internal validation, 27 presented only external validation, and 13 models had both internal and external validation. All of the articles were validated using an RF model, a tool mostly used for assessing the performance and robustness of the prognostic models, minimizing the effect of overfitting (a major cause for diminishing predictive ability for unknown data) or predicting the outcome (in 50% of the cases). The number and type of validation utilized for each cancer is visible in Table 4.

### Esophageal cancer (EC)

EC is generally divided into squamous cell carcinoma (SCC) and adenocarcinoma and typical treatments for EC are chemotherapy, radiation, and surgery (Acharya et al., 2023). Moreover, tumor stage and respectability typically determine the outcome of this type of cancer (Quail and Joyce, 2013). To conduct an improved clinical decision analysis, it is necessary to predict EC patients’ response to treatment, recurrence, and survival rates. Previous studies have applied machine learning models, especially RF, to predict these outcomes with remarkable success. RF is strong against high-dimensional data, where there are complex relationships among variables. The methodology employed by RF involves the creation of several uncorrelated decision trees, each derived from distinct random samples of the dataset. This will outperform classification and reduce overfitting in the models. In the OCCAMS Consortium study, RF was used in a group model with Elastic Net Regularized Logistic Regression and XGBoost to find out the recurrence rate of esophageal adenocarcinoma after surgery. An optimized version with hyperparameter tuning showed excellent predictive performance, especially in identifying patients at high risk of recurrence (Rahman et al., 2020). Another study on SCC looked at how RF could be used on patients to separate tumors that could be removed from their bodies from those that could not use radiomic features taken from CT scans. The model behaved with high accuracy AUCs and outperformed several other machine learning models (Ou et al., 2019). In a research initiative by GARF focusing on patients with locally advanced EC, RF was combined with a genetic algorithm to identify the most pertinent imaging and clinical characteristics that might predict treatment outcomes and patient lifespan (Paul et al., 2017). The RF classifier provided the minimum misclassification error and gave the maximum AUC score; thus, its strength in handling complex medical images and clinical data has been demonstrated (Chaddad et al., 2018). Generally, RF has constantly shown that it is one of the most powerful tools in providing predictions of the outcomes of EC, and hence, it has allowed relevant and important insight into patient management and treatment (Thavanesan et al., 2024).

RF has been successful in predicting the treatment responses, recurrence, and survival rates of EC patients. With RF, many decision trees are grown on random samples, which decrease the overfitting and increase the accuracy of prediction. Previous studies have shown the role of RF in regulating the outcome of ECs by demonstrating its ability to identify operable tumors and predict cancer recurrence accurately.

### Gastric cancer (GC)

Starting in the gastric mucosa, GC is a cancer that can progress from early-stage T1 GC to advanced metastatic illness (Rawla and Barsouk, 2019). The size and location of the tumor, the level of histological differentiation, and the existence of certain indicators like *Helicobacter pylori* infection are all factors that increase the chance of having this illness (Hallinan and Venkatesh, 2013). For the illness to be effectively managed, a precise evaluation of the patient’s prognosis—including survival rates, the possibility of distant metastases, and the reaction to chemotherapy—is essential (Stone et al., 2023). To forecast the clinical outcomes of GC patients, including distant metastases, serosal invasion, or survival, this research created machine learning models. These models have been created using tumor markers, radiomic characteristics taken from CT images, and clinical-pathological data. RF, SVM, and ANN are the main machine-learning algorithms examined in this regard. The AUC, sensitivity, and accuracy of these models were compared against one another. Notably, in the majority of the examined datasets, some RF models had competitive or better prediction accuracies, making them extremely predictive of patient outcomes (Tian et al., 2023; Li et al., 2018). To ensure that these models are more resilient across various datasets, external validation is helpful. In general, the ease of handling and processing a dataset with numerous variables has led to the widespread use of RF. The RF models were trained using clinical and radiomic data to predict the overall survival time of patients with GC who had serosal invasion or close metastases (Huang et al., 2021; Liu et al., 2021). RF ensembles various tree-only models, which reduces the risk of single-model overfitting and improves substantial prediction quality (Tian et al., 2023; Liu et al., 2023). RF demonstrated strong predictive performance by consistently achieving high AUC scores in each of these. For instance, in many situations, RF could accurately predict survival and metastasis; in some cases, it could even outperform other models like SVM or neural networks (Li et al., 2018; Liu et al., 2023). Moreover, the feature importance measures calculated by the RF models have pointed out the most critical factors affecting patients’ outcomes and helped clinical decision-making in the treatment of GC (Tian et al., 2023; Liu et al., 2021).

This research developed machine-learning models using tumor markers, CT image features, and clinical data to predict clinical outcomes in GC patients. The main algorithms studied were RF, SVM, and ANN, with RF often showing competitive prediction accuracy. RF models were trained to predict survival in patients with serosal invasion or metastases and identified key factors that influence outcomes, aiding treatment decisions.

### Colon cancer (CC)

CC originates in the colon or rectum and often presents with unnatural growths that can spread and invade adjacent tissues is known as CC (Alzahrani et al., 2021). This presented study aims to enhance the classification of CC by developing predictive models using the RF algorithm together with feature selection techniques. Methodological steps include the four major phases: acquiring gene expression data, model construction without and with feature selection, and comparative analysis. Data consisted of 62 CC samples, where 40 were tumor biopsies and 22 were normal controls. Before using feature selection approaches such as MDA and MDG to modify model performance, RF-based models were developed. The RF classifier is used here because of its excellent performance in managing complex, noisy, and high-dimensional data. RF works particularly effectively in datasets with large feature dimensions because it builds several trees from random picks and utilizes the outputs of those trees to generate predictions. In the present investigation, RF showed excellent prediction ability in distinguishing between normal tissues and CC (Yao et al., 2022). In turn, feature selection with the MDA and MDG methods improved the general performance of the model in terms of much higher accuracy, precision, recall, and F1 score because only the most relevant features were used for classification. The comparative analysis showed that the RF model’s predictive performance was improved by the feature selection procedure. Additionally, the use of RF in several settings in CC research points to its possible value for both predictive and diagnostic evaluations (Li et al., 2021). By adding more information like immune cell fractions, histopathological images, and genomic profiles (Zhou et al., 2019), RF was able to find key markers that set tumor tissue apart from normal samples. When RF was combined with LASSO, it became even easier to find the immune cells and genetic markers that were involved. This improved the accuracy of the prognostic and diagnostic models. This model’s strong performance was validated by tests that used AUC and Harrell’s concordance index (c-index), which are essential for forecasting a patient’s outcome and creating more accurate forecasts (Shafi et al., 2020). The RF consistently outperformed the other approaches in terms of classifying, predicting, and offering useful information about many aspects of CC outcomes.

Shortly, RF showed strong performance in distinguishing between normal and cancerous tissues. Using feature selection methods like MDA and MDG enhanced model accuracy and other performance metrics. RF also provided valuable insights for diagnostic and prognostic evaluations of CC outcomes (Table 5).

**Table 5:** Colon cancer (CC).

References	Dataset	Task	Approaches	Disease type	Performance (metric)	Results
Yao et al. (2022)	Gene expression data	Tumor classification (normal vs. tumor tissues)	Random Forest (RF)	Tumor/normal tissues	High classification accuracy	RF distinguished between normal and tumor tissues effectively with robust accuracy
Li et al. (2021) and Zhou et al. (2019)	Genomic, histopathological, and immune cell profiles	Biomarker identification	RF combined with LASSO, feature selection techniques (MDA, MDG)	Tumor/normal tissues	Improved accuracy, F1-score, precision, and recall with selected features	Identified immune cells and genetic markers crucial for diagnostic and prognostic models

### Rectal cancer (RC)

RC remains an important focus for predictive modeling across several studies, predicting improved outcomes and prognosis (Peng et al., 2007). Therefore, in one study, a total of 33 patients diagnosed with mid-to-lower rectal adenocarcinoma underwent NCRT, following which they received radical surgery. RF was applied to develop a predictive model that could provide a prediction about the tumor regression grade, which is a measure of response related to NCRT. The RF model was trained on the survival fraction data with 200 trees and tested by AUC, accuracy, and kappa values. Strong predictive capability and highly effective classification for treatment responses were shown for this RF model (Park et al., 2021). There was another study that involved 211 patients with locally advanced RC, which involved chemoradiotherapy and surgery. Radiomics features were taken from contrast-enhanced CT images taken before treatment, and RF was used to choose the most relevant features that could predict a pathological complete response. Feature ranking regarding its importance and minimizing errors was done by the approach known as the Fast Unified RF for Survival, Regression, and Classification, referred to as RF-SRC (Li et al., 2023). RF was combined with other machine learning methods, namely LASSO and SVM, to develop a radiomics score that predicted response to treatment, which is named Radscore. Based on several performance criteria, including improved AUCs, accuracy, and sensitivity, the suggested model appeared to perform well (Qiu et al., 2022). A radiomics nomogram was developed for clinical decision-making based on predictions by RF and clinical indicators in which, data from the Surveillance, Epidemiology, and End Results database and two hospitals in China supported a different study that developed a predictive model for liver metastasis in RC patients. RF was core in this process among the other machine learning algorithms. The model was trained with a well-established technique called 10-fold cross-validation, whereas SMOTE was used to handle class balancing (Li et al., 2023). Performance was quantified using AUC, accuracy, sensitivity, specificity, and F1-score, and in this regard, it was robust for the RF model. Feature importance was evaluated by permutation importance, while the SHAP method gave further insight into the contribution of each variable. RF has always been good at working with big datasets, picking out the most important features, and then building a good model for predicting what will happen with RC, like TRG response, and likelihood of pCR. In these models, its application underlines effectiveness in improving decision-making and planning treatments for RC patients (Andersson et al., 2024).

Here, RF selected relevant features and combined them with other machine learning methods to create a radiomics score, named Radscore, which performed well on various metrics. Additionally, RF was used in creating a predictive model for liver metastasis, demonstrating effectiveness in handling large datasets and improving treatment decisions for RC patients (Table 6).

**Table 6:** Rectal cancer (RC).

References	Dataset	Task	Approaches	Disease type	Performance (Metric)	Results
Park et al. (2021)	Clinical survival fraction data	Tumor regression grade (TRG) prediction	Random Forest (RF), 200 decision trees	Mid-to-lower rectal adenocarcinoma	Strong predictive capability (high AUC, accuracy, kappa values)	RF effectively predicted TRG responses post-NCRT with high accuracy
Li et al. (2023) and Qiu et al. (2022)	Radiomics data from contrast-enhanced CT scans	Pathological complete response (pCR) prediction	RF-SRC (Fast Unified RF for Survival, Regression, and Classification), LASSO, SVM	Locally advanced rectal cancer	High AUC, sensitivity, specificity, and predictive accuracy	Combined models (RF and radiomics) accurately predicted pCR, aiding clinical decisions
Andersson et al. (2024)	Clinical and imaging data	Liver metastasis prediction	RF combined with radiomics nomogram, 10-fold cross-validation, and SMOTE for balancing	Advanced rectal cancer	High AUC, feature importance explained with SHAP, robust predictive model	RF identified critical predictors for liver metastases using SMOTE and cross-validation

### Colorectal cancer (CRC)

**Figure 2:**
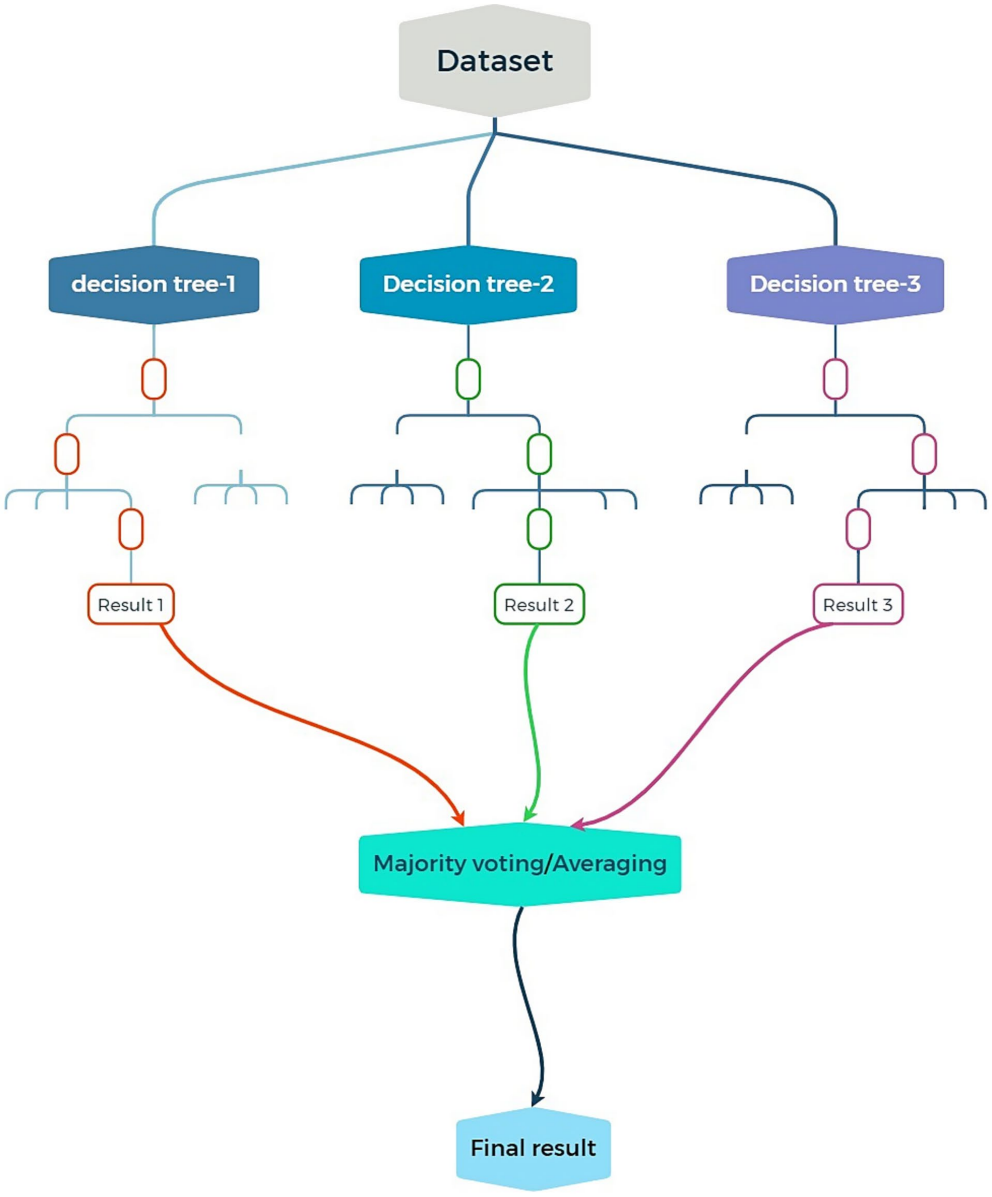
Schematic illustration of an RF model: combining multiple decision trees to improve accuracy and reduce overfitting in predictions.

CRC is a malignancy that can develop in the colon or rectum. When it reaches an advanced level, it might cause issues like stage IV liver metastases, which calls for intricate therapeutic measures (Kow, 2019). Chemotherapy, radiation therapy, and surgery are the main forms of treatment. The prognosis of patients is significantly impacted by many variables, such as the kind of cancer, histological aspects, and genetic features, including KRAS mutations (Mishra et al., 2013). Particularly for lymph node metastases, exact survival and metastatic risk prediction are crucial for good treatment planning (Kim and Choi, 2019). To predict patient outcomes, including survival and the chance of metastasis, this research was designed to take advantage of machine learning techniques, namely the RF algorithm. In one study, we used receiver operating characteristic analysis to assess the performance of an RF model we constructed based on radiomic characteristics taken from MRI images, which showed a very high predicted accuracy of patient survival (Takamatsu et al., 2019). Another study of stage IV CRC patients developed an RF model to predict the risk of lymph node metastasis (LNM) based on a wide range of clinical and imaging features (Daye et al., 2021). The AUC values >0.90 (Tabari et al., 2024), reflect these models’ extremely high predictive power. In each of the reviewed investigations, the most commonly used was the RF algorithm. Researchers accepted the use of RF in analyzing variant clinical, radiomic, and imaging data to predict both survival outcomes and LNM risk. As illustrated in Figure 2, RF constructs multiple decision trees based on randomly selected features from the dataset to reduce overfitting and optimize accuracy. The capacity to categorize patient outcomes based on survival or risk of LNM is demonstrated by the RF models’ AUC values. Since they show which elements—such as radiomic or clinical features—are more pertinent to the model’s predictions, these feature importance measurements were quite helpful. Because of this, the outcomes were simpler to comprehend (Pourhoseingholi et al., 2017).

Overall, predicting patient outcomes using machine learning techniques, particularly the RF algorithm, is key for effective treatment planning. Studies showed high accuracy in predicting survival and lymph node metastasis risk using RF models based on various clinical and imaging data. Feature importance measures helped clarify the most crucial prediction elements (Tables 7, 8).

**Table 7:** Colorectal cancer (CRC).

References	Dataset	Task	Approaches	Disease type	Performance (metric)	Results
Takamatsu et al. (2019)	Radiomic features extracted from MRI scans	Survival prediction	Random forest (RF)	Stage IV CRC	High AUC, strong predictive accuracy	RF achieved high predictive accuracy for survival outcomes based on radiomic features
Daye et al. (2021) and Tabari et al. (2024)	Clinical and imaging data	Lymph node metastasis (LNM) prediction	RF with feature selection	Advanced CRC	AUC > 0.90, identified most crucial predictors (e.g., clinical, radiomic features)	RF identified key radiomic and clinical predictors of LNM, enhancing prognostic modeling

**Table 8:** Comparison of the included articles discussed 2 cancers, and 13 studies were both internally and externally validated.

Cancer	Model development	Model validation	Model d + v	Internal validation	External validation	Ex+In validation
Esophageal cancer	3	2	5	5	2	3
Gastric cancer	17	2	3	12	5	1
Colon cancer	8	1	0	6	2	1
Rectal cancer	6	1	2	5	3	1
Colorectal cancer	21	6	2	15	10	4

## Discussion

This systematic review shows the effectiveness of randomized RF models in detecting and predicting GI cancers. RF models have consistently shown good accuracy and reliability in diagnosing GI tract malignancy types, including EC, RC, GC, CC, and CRC, in several investigations (Zhang et al., 2023; Kolisnik et al., 2023). They also provided reliable predictions for patient outcomes, including survival rates and the likelihood of relapse (Zhang et al., 2023). RF’s strengths, such as managing complex and heterogeneous datasets and handling missing values, make it an attractive tool for doctors and researchers. In addition, RF models are known for their ability to combine molecular, clinical, and pathological data, thus providing a holistic approach to cancer prediction (Xu et al., 2022).

The findings of the research show that RF-based models are more effective than traditional prediction methods that depend on statistical methods and experimental guidelines in predicting outcomes associated with GI cancer. The reason RF is better than conventional models is because it can capture nonlinear correlations and interactions between variables. Intrinsic algorithms can reduce the nature of excessive risk. Also, these algorithms can increase the generalizability of RF to diverse data sets. In addition, RF’s ability provides a more holistic approach to understanding the progression of cancer to integrate multimodal data, such as genetic, clinical, and demographic factors, potentially leading to more personalized treatment strategies. These results imply that RF models may effectively address the deficiencies in treatment prediction and decision-making associated with GI cancers, which are not adequately managed by conventional markers (Afrash et al., 2023; Liu et al., 2024). RF-based models are valuable tools for early detection and accurate prediction, which is essential to improve survival rates in patients with GI cancer (Alghafees et al., 2024). Application of these models helps healthcare providers to better diagnose high-risk patients and also determine whether immunotherapy or aggressive interventions are beneficial to them (D'Orsi et al., 2024). Furthermore, the adoption of RF models leads to saving costs by optimizing the use of healthcare resources and reducing diagnostic errors (Orji and Ukwandu, 2024). The results suggest the importance of RF models for GI cancers and that these models have a vital role in the future of personalized medicine in oncology (Chen et al., 2024).

### RF strengths and applications

The effectiveness of Random Forest (RF) models in identifying and forecasting the course of gastrointestinal (GI) malignancies is highlighted in this comprehensive study. Several studies utilizing RC, GC, and CRC have shown the significant accuracy of RF models (e.g., Rahman et al., 2020; Qiu et al., 2022 RF’s ability to comprehend complex, high-dimensional data and accommodate for missing values makes it a valuable approach in the research of cancer). Additionally, to enhance survival prediction and personalized treatment plans for patients with GI cancer, RF models use multimodal data, such as genetic, clinical, and pathological features (Xu et al., 2022). For example, studies on CC and RC have shown that using RF models in conjunction with feature selection methods such as LASSO greatly improved prediction accuracy (Li et al., 2021; Qiu et al., 2022).

As mentioned earlier, Random Forest models are naturally robust to missing values, and many studies leveraged this strength without extensive preprocessing. Most of the included studies did not mention their approach to handle missing data, but still successfully trained RF models. Given RF’s built-in mechanisms for handling gaps, we consider this acceptable for the scope of our review, though future work could benefit from more detailed reporting of imputation strategies.

### Interpretability of RF models

Even though RF models are strong, clinical practitioners nevertheless have a significant difficulty with their interpretability. Effective use of techniques like SHAP values and permutation significance can improve decision-making transparency. While permutation significance analysis aids in determining the most important factors influencing outcomes, SHAP values offer insights into how each feature contributes to model predictions, allowing doctors to have a more nuanced view (Ali et al., 2023). Additionally, by including feature significance charts in clinical reports, doctors may be able to have a better understanding of the key predictors and use the results of the RF model to influence their treatment choices (Sadeghi et al., 2024).

### Clinical applicability

To successfully incorporate RF models into clinical procedures, a few obstacles need to be removed. Medical professionals’ lack of technical expertise is one of the main problems; many of them might not understand machine learning principles or particular RF applications well enough (Esmaeilzadeh, 2024). Furthermore, because hospitals can lack the infrastructure required to support more complex modeling techniques, incorporating these sorts of tools into standard clinical practice could be difficult. By developing automated, user-friendly RF-based clinical apps and enhancing training programs to educate medical personnel about machine learning, these problems may be addressed and they will be prepared to deploy machine learning in patient care (Stoumpos et al., 2023).

### Limitations

Despite the sector’s extremely positive results, several limitations must be noted. One major problem discouraging physicians from using RF algorithms in their daily practice is their complicated clinical applicability. For instance, in research on EC analysis, different clinical conditions and patient characteristics may yield surprising model performance, and hence physicians cannot consistently apply the results. Moreover, most of the included studies are retrospective, which restricts the generalizability of their findings since they cannot precisely reflect the variety that would have been present in real clinical settings because they usually rely on data that has already been collected. For example, because the majority of research predicting GC outcomes relied on historical data that could be subject to selection bias or lack adequate patient demographics, results often were biased and the model’s dependability was poor in new and diverse patient populations.

Moreover, there are also problems concerning consistency with specific input variables in the RF models identified across the different research studies. Indeed, small changes in the imaging data, biomarkers used, or clinical scenario can greatly influence the performance and consistency of such models. These differences render it challenging to confirm findings across patient groups or various healthcare settings, while limiting comparisons across studies. In this respect, the present work aims to point out that the application of machine learning models, such as RF, in various settings regarding patient demographics and treatment should be demonstrated, as well as the need for carefully designed prospective clinical trials and the need for external validation. To realize the full potential of predictive modeling in enhancing cancer treatment and patient outcomes, several steps should be taken.

### Future directions

To increase the efficacy and usefulness of RF models in cancer, future research should focus on developing user-friendly interfaces that facilitate their integration into clinical procedures. Standardizing input variables is necessary to improve repeatability across investigations, and prospective trials and extensive validations across a range of patient groups are needed to evaluate the model’s effectiveness. Furthermore, examining hybrid approaches that combine RF and neural networks may significantly improve predictions for complex outcomes like survival and metastasis, ultimately leading to better patient care and clinical judgment. Further, to ensure that these prognostic tools generalize across diverse patient populations and can be safely deployed in clinics, future efforts should prioritize prospective, multi-center external validation and real-world integration studies.

## Conclusion

This extensive review shows that machine learning models are very reliable and accurate in finding out and forecasting GI malignancies like EC, RC, GC, CC, and CRC. Moreover, there exists a large body of literature on how well RF handles such intricate pathological and molecular clinical data for the accurate forecasting of patient outcomes related to survival rates and recurrence risks. On the other hand, traditional prediction algorithms could not identify complex patterns or even recognize and control the diversity of GI malignancies. The results of this study identify the importance of RF models in improving the accuracy of the prediction that will inform treatment options for patients with GI cancers. The ability of these models to handle high volumes of diverse data and identify nonlinear relationships between variables makes them efficient in clinical applications. It aids doctors to better identify high-risk patients and adopt more appropriate treatments for them. RF has been able to act as a powerful tool to reduce the distances in treatment predictions, especially in cases where traditional prediction markers are inadequate.

RF-based models are promising to have a huge say in future developments related to customized medicine. The integration of the models into current treatment strategies will likely improve survival rates and the general quality of life in patients with GI cancers. Cost savings can be realized through the use of the models by reducing diagnostic mistakes and improving healthcare resource distribution. Further studies are needed to be carried out to encourage wide application of the models in real-world scenarios.

## Data Availability

The original contributions presented in the study are included in the article/Supplementary material, further inquiries can be directed to the corresponding author.
