# Supplementary material for: The application of random forest-based models in prognostication of gastrointestinal tract malignancies: a systematic review
Source: Front Artif Intell. 2025 Jul 18;8:1517670. doi: 10.3389/frai.2025.1517670 (PMC12315591; doi:10.3389/frai.2025.1517670)
Supplement: Supplementary file 1 [file Table_1.DOCX]

| **Author** | **Title** | **Year** | **Country** | **Aim of study** | **Population** | **Type of pathology** | **Use of Random Forest in the model** | **Model development or Validation** | **Type of validation** | **Model used for validation** | **Factor(s) used for prognostication** | **Method** | **Outcome** | **Conclusion** | **Risk of bias** |
| --- | --- | --- | --- | --- | --- | --- | --- | --- | --- | --- | --- | --- | --- | --- | --- |
| Ma, N., et al. | Bioinformatics evaluation of a novel angiogenesis related genes-based signature for predicting prognosis and therapeutic efficacy in patients with gastric cancer(52) | 2022 | USA | based signature for  predicting prognosis and therapeutic efficacy in  patients with gastric cancer | not provided.  (36 ARGs) | Gastric cancer (GC) | identifing critical genes and construct a diagnostic  predictive model | model development | internal validation | RF,LASSO, SVM, and  XGBoost | univariate and multivariate Cox proportional hazard  regression analyses | multivariate lasso-based  logistic regression analysis | the survivals of patients with GC were significantly  decreased in the high-ARG  score group  compared with those in the low-ARG score  group | the ARG signature may act as an important  prognostic prediction marker for GC patients | Low |
| Lv, L., et al. | Radiomic analysis for predicting prognosis of colorectal cancer from preoperative 18F-FDG PET/CT(53) | 2022 | China | to leverage a combination of clinical, biological, and  radiological data to build a predictive model for  colorectal cancer prognosis | 196 | Colorectal cancer | assess the performance and robustness of the prognostic models. | model development | internal & external validation | Random Survival Forest (RSF) | C-index,CI,Moreover, four features (including two  clinical features and two  radiomics features), tumor metabolic factors, such as SUVmean, SUVmax. | Preoperative clinical factors, serum tumor markers,  F-FDG PET/CT protocol and imaging interpretation,  Medical image delineation, Feature Engineering,  Random Survival  Forest ,statistical analysis | The ability to distinguish  low-risk and high-risk patient subgroups suggests these  models have the potential to improve prognostic  assessment and inform  personalized treatment  approaches for colorectal cancer patients. | F FDG PET/CT radiomics combined with clinical  features in the study may  fuel the identifcation of new imaging biomarkers, and  could be instructive in the  predictive prognosis of  colorectal cancer, especially in  stage III | Low |
| Lukomski, L., et al | Analyzing the Impact of Oncological Data at Different Time Points and Tumor Biomarkers on Artificial Intelligence Predictions for Five- Year Survival in Esophageal Cancer(54) | 2024 | Germany | evaluate the predictive  efficacy of AI methods for survival prognosis across different time points of  oncological treatment | 1002 | colorectal cancer | The models were validated using random survival forest analysis and showed  significant stratification of low-risk and high-risk  groups | model validation | external validation | two Machine Learning (ML) methods, Random Forest and XG-Boost, as well as  two Deep  Learning (DL) algorithms, Artificial Neural Networks and TabNet | histopathological lymph node status (pN),  histopathological tumor size (pT), clinical tumor size  (cT), age at the time of  surgery, and postoperative tracheostomy. | The BL dataset included:  Artificial Intelligence (AI),  including Machine learning (ML) and Deep  Learning (DL), The PS  dataset with biomarkers, the important predictive features included patient age at the  time of surgery,  TP-53 gene mutation,  Mesothelin expression,  thymidine phosphorylase  (TYMP) expression, NANOG homebox protein  expression, and indoleamine 2,3-dioxygenase (IDO) expressed on tumor-  infiltrating  lymphocytes, as well as  tumor-infiltrating Mast- and Natural killer cells | Model training on the BL dataset demonstrated  similar predictive  performances for all models but the AI models  outperformed LR when  trained with the PS datasets | Different  AI methods similarly predict the long-term survival status of patients with EC and  outperform LR the state-of- the-art classification model.  Survival status can be  predicted with similar  predictive  performance with patient data at an early stage of treatment when utilizing additional biomarker  analysis. This suggests that individual survival  predictions can be made  early in cancer treatment  by utilizing biomarkers,  reducing the necessity for  the pathological TNM status post-surgery | Moderate |
| Lu, T., et al | Establishment of a prognostic model for gastric cancer patients who underwent radical gastrectomy using machine learning: a two-center study(55) | 2023 | China | a prognostic model was  developed for gastric cancer patients who  underwent radical  gastrectomy using machine learning, employing  advanced  computational techniques to investigate postoperative mortality risk factors in  such patients. | 295 | Gastric cancer (GC) | RF, as an extension of the DT method, combines  multiple  DTs, with the majority vote  among the trees determining the final class  prediction of the model. RF incurs a substantial training cost, and the  decision-making process of the model is susceptible to the specific  division offeature values | model development | external validation | logistic regression (LR),  decision tree (DT), random forest  (RF), and gradient boosting machine (GBM). Model  performance  was assessed by comparing the area under the curve  (AUC) for each model | age, sex, maximum  tumor diameter, nerve or vascular invasion, TNM  stage, gastrectomy type, lymphocyte count, and  carcinoembryonic antigen (CEA) level | LR-based nomogram model & four machine learning  models | The nomogram model,  constructed based on LR, demonstrated excellent  clinical prognostic  evaluation capabilities. | Machine learning algorithms are robust performance  assessment  tools for evaluating the  prognosis of gastric cancer patients who have  undergone  radical gastrectomy. The LR- based nomogram model can aid clinicians in making  more reliable clinical  decisions | Low |
| Lu, L. B., et al. | Development and validation of serological dynamic risk score to predict outcome in gastric cancer with adjuvant chemotherapy: a multicentre, longitudinal, cohort study(56) | 2024 | China | We aimed to develop a risk score in real-time predicting outcomes for postoperative GC patients using blood  chemistry tests. | 2737 | Gastric cancer (GC) | the randomforest  recursivefeature  elimination (RFRFE)  algorithm was employed in  parallel for factor  selection. | model development | internal & external validation | The least  absolute shrinkage and  selection operator (LASSO) and random forest recursive feature elimination (RF-RFE) | The Cox regression model derived six risk factors to construct a  composite score (low-risk: 0-2 score; high risk: 3-6 score), including CEA,  CA125, CA199,  haemoglobin, albumin, and neutrophil to lymphocyte  ratio | We gathered demographic information, clinical or  pathological  characteristics, laboratory  test results, and outcome  data of the training cohort  from the database of Xijing  Hospital . Survival was  calculated using the Kaplan–  Meier method with a  log-rank test , and  univariate comparisons were  performed using an  unadjusted Cox model . Missing longitudinal data  were handled  usingmultiple imputations (MI) with a random forest. | we identified CEA, HGB, CA125, ALB, CA199, and NLR as  the six most important prognostic indicators for patients with GC  after surgery. In the current  study, we found a  progressive improvement in the accuracy of the HI-GC risk score over time after  surgery.  Notably, the HI-GC risk  score demonstrated superior predictive  performance in patients who completed adjuvant chemotherapy while  showing a relative deficiency within 1 year. | The dynamic risk score is an accurate and userfriendly  serological  risk assessment tool for  predicting outcomes and assisting clinical decisions after gastrectomy | Low |
| Long, X., et al. | Identification of critical genes to predict recurrence and death in colon cancer: Integrating gene expression and bioinformatics analysis(57) | 2018 | China | The purpose of this study was to screen the critical genes for future diagnosis and treatment of colon  cancer by bioinformatics method. | none (only gens) | Colon adenocarcinoma , Adrenocortical carcinoma , Bladder Urothelial  Carcinoma , Breast invasive  carcinoma , Cervical  squamous cell carcinoma and endocervical  adenocarcinoma | to screen gene sets related to the prognosis in DEGs. | model development | external validation | Random forest survival  model and KEGG pathway enrichment | differently expressed genes (DEGs). gens: HSP90AB1, VCAN,  CLDN2, EPHB6, EIF3E, GSPT1, PRKDC, RPS2, GARS  etc. | bioinformatics approaches to identify gene alteration  that contribute to colon  cancer progression via  analysis of TCGA RNA  sequencing data and other publicly GEO microarray  data. Random forest survival model and KEGG pathway  enrichment analysis , gene  ontology (GO) , search tool for the retrieval of interacting genes (STRING) database  version 10.0 on line. | These data demonstrated  VCAN were associated with tumorigenesis and may be as biomarker for  identification of the  pathological grade of colon cancer | In conclusion, we  demonstrated for the first time that  VCAN is over-expressed in colorectal cancer and  VCAN promotes colorectal cancer cell growth in vitro. These data suggest VCAN might serve as a potential target in the diagnosis and/ or treatment in colorectal  cancer. | Moderate |
| Liu, Y., et al | Identification of high-risk factors associated with mortality at 1-, 3-, and 5-year intervals in gastric cancer patients undergoing radical surgery and immunotherapy: an 8-year multicenter retrospective analysis(58) | 2023 | China | This research endeavors to devise a  machine learning algorithm to recognize risk factors with a high probability of  inducing mortality among patients diagnosed with  gastric cancer, both prior to and during their course of treatment | 1015 | gastric cancer: lymphnode metastasis,peripheral nerve metastasis, H. pylori  infection | we employ an iterative  methodology to dynamically modify the  model’sparameters  and observe its outcomes, aiming to ascertain the  model parameters  that yield optimal results | model development | internal & external validation | extreme gradient boosting  (XGBoost), random forest  (RF), and k-nearest neighbor algorithm (KNN) | age, tumor invasion, tumor lymph node metastasis, tumor peripheral nerve  invasion (PNI),  multiple tumors, tumor size, carcinoembryonic antigen  (CEA) level, carbohydrate antigen 125 (CA125) level, carbohydrate antigen 72-4 (CA72-4) level, and H.  pylori infection. | The models  (XGBoost ,RF ,KNN ) were subjected to internal  validation through  employment of the k-fold cross validation technique, and subsequently, an  external dataset was utilized to externally validate the  models | the XGBoost algorithm  demonstrated superior  predictive capacity regarding  the risk  factors that affect mortality after combination therapy in gastric cancer patients  for a duration of one year, three years, and five years posttreatment. | The XGBoost algorithm can assist clinicians in identifying pivotal  prognostic factors that are of clinical significance and can contribute toward individualized patient  monitoring and  management. | Low |
| Liu, Y., et al | Gut Microbiota-  Based Algorithms in the Prediction of  Metachronous  Adenoma in  Colorectal Cancer Patients Following Surgery(59) | 2020 | China | study suggests that gut microbiota is a potential predictive  biomarker for MA. | 41 | Colorectal cancer (CRC) | To determine the potential of  bacterial taxa in  discriminating MA, we aimed to identified a minimal set of bacterial genera that  maximally  differentiated nMA from MA. | model validation | external validation | multivariate logistic  regression analysis and RF | Escherichia–Shigella, BMI and synchronous-adenoma | Cold biopsy forceps , Fecal samples and All the samples were snap-frozen in cryovial immediately  following collection and stored at 80C until DNA extraction. | (1)There was a significant  correlation between pre-  operative gut microbiota and the development of MA  among CRC patients after surgery. (2) Specific  members of the predominant gut microbiota, including  Escherichia–Shigella and  Acinetobacter,were identified as independent risk factors  for MA. (3)The  microbiotabased RF model was established utilizing  these specific members of predominant gut microbiota combined with independent clinical riskfactors (BMI) and the status of synchronousadenoma,  showing a good  performance (AUC,0.885) to predict MA among CRC  patients after surgery.(4)The microbiota-based RF model exhibited good ability inthe prediction of MA using  fecaland off-tumorsamples (AUC,0.835 and0.889, respectively). (5)Arisk-  scoring system was  proposed with four independent predictive  factors got an AUC  of 0.94and0.835 for  thepredictionofMAindiscover yand  validationcohort. | specific members of the  dominant gut microbiota as non-invasive biomarkers for prediction of MA or CRC  after surgical resection. The newly established RF  algorithm and the risk-  scoring system have a good ability to predict the  development of MA after surgical resection, and  therefore, the novel  approaches hold potential to  guide individual post-  operative surveillance plan for CRC patients in future clinical application. | Moderate |
| Liu, Y., et al. | Identification of high-  risk factors for  recurrence of colon cancer following  complete mesocolic excision: An 8-year retrospective study(60) | 2023 | China | study aimed to develop a  machine learning model that can identify high-risk factors before, during, and after  surgery, and predict the  occurrence of postoperative colon cancer recurrence. | 1187 | Colon cancer , Colorectal cancer | all the models were utilized to: (1) score and rank the  significance of all the  variables. Variables that  appeared in the top ten  rankings in all four models and were also significant in both univariate and  multivariate regression  analyses were chosen (2)  Evaluation and development of prediction models | model development | external validation | extreme gradient boosting  (XGBoost), random forest  (RF), support vector machine (SVM), and k-nearest  neighbor algorithm (KNN) | pre operative valuable:  patient demographics  (gender, age, smoking  history, alcohol history, and body mass index), basic  clinical characteristics  (American Society of  Anesthesiologists score,  nutrition risk screening 2002  score, surgical history,  disease duration, adjuvant chemo therapy history, and adjuvant radiotherapy  history), basic medical  history (anemia, diabetes, ileus, hypertension,  hyperlipidemia, and coronary artery disease), laboratory  tests (albumin,  carcinoembryonic antigen,  carbohydrate antigen 19–9, carbohydrate antigen 125,  and carbo hydrate antigen  72–4), tumor characteristics (T-stage, N-stage, peripheral nerve invasion, vas cular  invasion, tumor size, tumor  number, tumor conﬁguration, and pathologic type)  Intraoperative variables:  collected included surgical approach, type of surgery, duration of surgery,  intraoperative bleeding,  number of surgically cleared lymph nodes, and whether it was an emergency surgery.  Postoperative variables  collected included laboratory test indices (carcino  embryonic antigen,  carbohydrate antigen 19–9, carbohydrate antigen 125, carbohydrate antigen 72–4, procalcitonin, C-reactive  protein, serum amyloid A,  and neutrophil to lympho cyte ratio) and tumor  characteristics (tumor  recurrence). | clinical data from a database of colon cancer patients ,  The statistical software  programs SPSS and  R ,Univariate and  multivariate regression  analyses were conducted and four predictive model (XGBoost,RF,KNN,SVM) | The calibration curve  analysis showed that the  predicted probabilities from the XGBoost model were  well-calibrated with the actual probabilities. The Brier score of  XGBoost was the lowest  among the four models,  indicating good accuracy of the predicted probabilities. The DCA curves showed  that all four models had a  net clinical beneﬁt, with  XGBoost having the highest net beneﬁt at most  probability thresholds After comprehensive comparison, the XGBoost algorithm was chosen to construct the  predictive model . in this  study The results obtained  from the ROC curve showed an AUC value of 0.91 for the external validation set, which is a strong indication that the prediction model has high  accuracy in determining the occurrence of the disease | The model identifies  postoperative tumor  recurrence as a significant  obstacle in the management  of CME after sur gery,  highlighting factors such as postoperative CEA, tumor  size, lymphatic and liver  metasta sis, and number of tumors as closely associated  with the risk of recurrence | Low |
| Liu, T., et al. | Four transcription profile–based  models identify novel  prognostic  signatures in  oesophageal cancer(61) | 2020 | China | identifing novel prognostic signatures in oesophageal cancer | 159 | Oesophageal cancer (ESCA) | algorithms were used to identify the potential  prognostic markers .  Coefficients from adjusted Cox regressions were used to construct prognostic  signatures. Random Forest- Feature Selection(RFS-FS) and Support Vector  Machine-Recursive Feature Elimination (SVM-RFE) were applied to rank marker  importance | model development | internal & external validation | two regularization semi-  parametric algorithms (AIC and LASSO Cox models) and two machine learning algorithms (RFS and SVM classifiers) | Age (years) ＜60 ≥60  Gender Male Female  Local invasion T1 T2 T3 T4 TX  Lymph node metastasis N0 N1 N2 N3 NX  Distant metastasis M0 M1 MX  Type ESAD ESCC | The random assignment was conducted blindly by a  computerized random  assignment , the association between differentially  expressed genes (DEGs) and overall  survival (OS) was assessed with the Kaplan-Meier  survival analysis. In the  training phase, Akaike  information criterion (AIC)  adjusted Cox regression,  least absolute shrinkage and selection operator (LASSO) adjusted Cox regression | data showed that the  LASSO signature developed in this study could stratify  ESCA patients into good and poor survival groups  effectively. These results  were further validated in the internal ESCC dataset and external independent ESCC cohort.  Furthermore, FABP3, the most heavily weighted  marker in prognostic  signature, was identified as a  novel oncogenic gene in  ESCC. In summary, we  combined four algorithms, included two types of  adjusted Cox regressions and two machine learning algorithms, to investigate RNA-Seq, miRNA-Seq and adjuvant clinical data of  ESCA. | results demonstrated that  constructed signatures are potential prognostic tools to predict mortality risk in  ESCA and ESCC, and  FABP3 is a novel biomarker  and newly identified  oncogenic gene in ESCC. | Low |
| Liu, S., et al. | Prediction of serosal invasion in gastric  cancer: development and validation of  multivariate models  integrating  preoperative  clinicopathological features and  radiographic findings based on late arterial phase CT images(62) | 2021 | China | To develop and validate multivariate models  integrating endoscopic  biopsy, tumor markers, and CT findings based on late arterial phase (LAP) to  predict serosal invasion in gastric cancer (GC) | 154 | Gastric cancer (GC) | The multivariate model  generated by RF showed  best performance in the four machine learning algorithms with an AUC of 0.865. The  developed model based on RF was also applied in the validation cohort and  achieved an AUC of 0.845. | model validation | external validation | least absolute shrinkage and selection operator (LASSO) for dimension reduction.  support vector machine  (SVM), random forest (RF), artificial neural network  (ANN) and k nearest  neighbors (KNN), with the LASSO | The preoperative  differentiation degree, tumor markers, CT morphological  characteristics, and CT value related and texture  parameters , The  differentiation degree, and  multiple CT findings based  on LAP differed significantly between T1–3 and T4 GCs in the primary cohort (all P <  0.05) , Six serum tumor markers, including alpha fetoprotein (AFP),  carcinoembryonic antigen  (CEA), carbohydrate antigen (CA) 125, CA199, CA724,  and CA242 | Endoscopic biopsy , CT  examinations , Image  analysis (Morphological  characteristics , CT value- related parameters , CT  texture analysis) | In conclusion, we developed and validated multivariate  models integrating  preoperative  clinicopathological fea tures and radiographic findings  based on LAP CT images to predict serosal invasion in GCs, and it achieved a  favorable performance | We developed and validated multivariate models  integrating endoscopic  biopsy, tumor markers, CT morphological  characteristics, and CT  value-related and texture  parameters to predict  serosal invasion in GCs and achieved favorable  performance | Moderate |
| Liu, J., et al. | Transcription factor expression as a  predictor of colon  cancer prognosis: A machine learning  practice(63) | 2020 | USA | to identify the fundamental transcript factors, which are associated with clinical out  comes of colon cancer  patients, by implementing an in novative cancer prognosis signature discovery process that combines the random  forest algorithm with classic Cox Proportional Hazard  (Cox PH) method. Our study will emphasize on only using TFs expression data to  conduct prognostic analysis and we will provide a new  perspective on how we can better use gene expression profiles to conduct  prognostic research. By  using proposed workflow, a TFs based prediction model has been success fully  developed for colon cancer prognosis | 925 | Colon cancer | random forest method, is  performed to refine further and reduce the TFs. Based on the RF training re sults, the most significant TFs are selected based on the  top feature importance of RF | model development | external validation | Cox PH , RF . prediction  power is tested by accuracy analysis. Further more, the  predictive model is validated on colon cancer datasets,  collected from GEO  database | A five-transcription-factors based predictive model for colon cancer prognosis has been developed byusing  TCGA colon cancer patient data. Five transcription  factors identified for the  predictive model is HOXC9, ZNF556, HEYL, HOXC4 and HOXC6. | we evaluate the prognostic prediction power by  combining the Cox PH  model with the random  forest algorithm . We picked five  top-ranked transcription  factors and built a prediction model by using Cox PH  regression. Using Kaplan- Meier  analysis, we validated our predictive model on four independent publicly  available datasets | Kaplan-Meier curve and log- rank tests were conducted  on both training and  validation datasets, the  difference of overall survival time between predicted low and high-risk groups can be clearly observed. Gene set enrichment analysis was  performed to further  investigate the difference  between low and high-risk  groups in the gene pathway level. The biological meaning was interpreted. Overall, our results prove our prediction model has a strong  prediction power on colon cancer prognosis | Transcription factors can be used to construct colon  cancer prognostic signatures with strong prediction power.  The variable selection  process used in this study has the potential to be  implemented in the  prognostic signature  discovery of other cancer  types. Our five TF-based  predictive model would help with understanding the  hidden relationship between colon cancer patient survival and transcription factor  activities. It will also provide more insights into the  precision treatment of colon  cancer patients from a  genomic information  perspective | Low |

| Liu, G., et al. | Regulatory activity based risk model  identifies survival of  stage II and III  colorectal carcinoma(64) | 2017 | China | we utilized the activity of  regulatory factors, univariate Cox regression and random forest for variable selection  and developed a multivariate Cox model to predict the  overall survival of Stage II/III colorectal carcinoma in  GSE39582 datasets | 469 | Colateral carcinoma | random forest was  implemented for variable hunting :variable hunting was performed with 100  replications and 100 steps | model development | external validation | Survival analysis, Cox  univariate regression, Cox multivariate regression and randomForestSRC | ten regulators’ activities  (EPAS1, TP73, TEAD1, DBP, NME2, GFI1, NR5A1, ELK1, NANOG and ETS2) were selected as candidate  features (regulators) | we utilized the activity of  regulatory factors, univariate Cox regression and random forest for variable selection. This finding was further  validated in five other  independent datasets  (GSE14333, GSE17536,  GSE17537, GSE33113, and GSE37892). A nomogram  including risk score was plotted to facilitate the utilization of risk score | The hazard ratios <1  suggested that their  corresponding regulators were tumor suppressor  genes, while genes with hazard ratios >1 were  cancerous genes. the risk score was an important  survival indicator for stage II and III colorectal carcinoma. These results above indicate that our risk score model  was robust across datasets. | In conclusion, our  transcription activity based risk score model  successfully predicts the  survival of stage II and III  colorectal carcinoma. To our  knowledge, this  is the first model using  activities regulatory factors  to predict survival of stage II/ III colorectal carcinoma. | Moderate |
| --- | --- | --- | --- | --- | --- | --- | --- | --- | --- | --- | --- | --- | --- | --- | --- |
| Liu, D., et al | Machine Learning- Based Model for the Prognosis of Postoperative Gastric Cancer(65) | 2022 | China | we aimed at improving the prediction accuracy of  gastric cancer in  postoperation patients by constructing a highly  effective prognostic model | 955 | Gastric cancer (GC) | were used to screen and  recombine the features to  construct an ML prognostic model | model development | internal & external validation | Boruta algorithm, neural  network, support vector  machine, and random forest, Kaplan-Meier method | Immunomarker Support  Vector Machine (SVM)  Classifier for Prediction of  Gastric Cancer Survival. the classic clinical prognosis  model was directly  constructed by LASSO  regression analysis, and the cp-riskcore of each patient. | SEER database, The  LASSO regression method  was used to construct a  clinical prognostic model.  Clinical information collected from the Affiliated Tumor  Hospital of Harbin Medical  University was used for  external verification , the  univariate Cox regression  model was used to analyze the relationship between  clinical features and survival time, clinical charachteristics too. The survival curve was  generated by Kaplan-Meier method and compared by log-rank | Experimental results  showed that the AUC values of 1, 3 and 5 years in the  training set, validation set  and external validation set of clinical prognosis model and ML prognosis model directly  established by LASSO  regression are all around 0.8 | Both models (ML & AUC)  can accurately evaluate the prognosis of postoperative patients with gastric cancer, which may be helpful for  accurate and personalized treatment of post operative patients with gastric cancer | Low |
| Lian, Q., et al. | DNA methylation  data-based  molecular subtype  classification and  prediction in patients with gastric cancer(66) | 2020 | China | to mine the specifc DNA methylation sites that  afected the prognosis for GC patients. | 398 | Gastric cancer (GC) | Trough the construction of the random forest classifer,  we found that the  classifcation accuracy of the model based on the training set was 82.35%. The area  under  the ROC curve reaches  0.795, | model development | external validation | univariate Cox model,  random forest classifer, The area under the curve | we used 22,062 methylation spectra to perform Cluster analysis, genes that are  enriched in multiple cancer- related pathways  of KEGG, including WNT  signaling pathway, FoxO  signaling pathway, cancer  signaling pathway and so on | We used the TCGA GDC API  to download the latest  clinical follow-up information (2019.01.04), which contains a total of 398 samples. A  univariate Cox proportional hazards regression model  was performed for each  methylation site and survival data. To identify the  molecular type of gastric cancer based on  methylation, we use QDMR software | The specifc genes were  enriched to the biological  pathways that were reported to be closely correlated with GC; moreover, the  subsequent transcription  factor enrichment analysis  discovered that, these genes were mainly enriched into  the cell response to  transcription factor B,  regulation of MAPK signaling pathways, and regulation of  cell proliferation and  metastasis. Eventually, the prognosis prediction model for GC patients was  constructed using the  Random Forest Classifer  model, and the training set and test set data were  carried out independent verifcation and test. | This study was based on the TCGA methylation profle of gastric cancer to identify  prognostic-specifc  methylation to construct a  classifer for gastric cancer.  Helps identify new molecular subtypes of gastric cancer. Tis classifer can provide  guidance for clinicians on  the diagnosis and prognosis of diferent epigenetic  subtypes. In addition, the  identifed subtype-specifc  molecules provide mul tiple targets for the precise  medical treatment of gastric cancer. | Low |
| Leonard, G., et al | Machine Learning Improves Prediction Over Logistic Regression on Resected Colon Cancer Patients(67) | 2022 | USA | The objective of this study was to demonstrate that ma chine learning techniques  could improve upon  traditional logistic regression methods by more accurately predicting a composite  outcome of unplanned 30-d readmission along with 30  and 90-d mortality after  colon cancer surgery using a  large national cancer  database. | 528'060 | Colon cancer | The Random Forest model  was built using the entire  training dataset with all  selected variables. The main hyperparameter for Random Forest is mtry, the number of variables randomly sampled as candidates at each tree  split.28 Values for mtry  typically begin at the square  root of the number of  variables, p and can range  from 2 to up to p.25 For this particular model, possible  mtry values included 2, 4, 6, and 8. By using 5-fold cross- validation on the training set, mtry was optimized at  4.AUC was 0.757 (95% CI: 0.752-0.762) and the Brier score was 0.089. The  accuracy was 0.889, and the  sensitivity and speci ficity were 0.089 and 0.993,  respectively. In terms of AUC, the Random Forest was the best model but  performed similarly to  XGBoost in accuracy and Brier score. | model development | internal & external validation | Machine learning algorithms used 5-fold cross-validation  for hyperparameter  optimization on the training set. eXtreme Gradient  Boosting, XGboost. | We included patients who  underwent colectomy with  curative intent. We excluded those with distal sites of  radiation and those without outcome data available. | Surgical colon cancer  patients were identified  using the 2013 National  Cancer Database (NCDB). a synthetic minority  oversampling technique  (SMOTE) was implemented and applied using XGBoost. The primary metric for model comparison was area under the curve (AUC). First, a  traditional logistic regression model was built without  performing the variable  selection. The second model was built using a machine  learning algorithm entitled  eXtreme Gradient Boosting, XGboost.15 This model  attempts to improve upon  tree-based methods by  learning from each tree with progressive and iterative  improvement. The third  model, Random Forest, also attempts to improve on  decision trees by growing many trees and then  selecting the majority  ‘opinion’.16 This ensemble method may prevent  overfitting | The entire training set was  used to build an XGBoost  model. Hyperparameter  optimization for the final  model was accomplished by 5-fold cross-validation on  the training set to maximize AUC. | We show that a large registry of surgical colon cancer  patients can be utilized to  build ML models to improve outcome prediction with  differential discriminative  ability. These results reveal the potential of these  methods to enhance risk prediction, leading to  improved strategies to  mitigate those risks. | Low |
| Low, Y. S., et al | A formalin-fixed  paraffin-embedded (FFPE)-based  prognostic signature to predict metastasis in clinically low risk  stage I/II  microsatellite stable colorectal cancer(68) | 2017 | Singapore | Our goal was to develop an  FFPE-based predictor of metastasis of left-sided, MSS, stage I and II  colorectal cancers. | (193 genes) | Colorectal Cancer (CRC) | Random Forest  implementation uses training data to derive the “out-of-  bag” estimate of  performance in new data which serves the same  purpose as those obtained  by cross validation . Random Forests also rank features  according to their  importance in prediction | model development | internal & external validation | Survival analyses (Kaplan- Meier, Cox proportional  hazards) were performed in the Python lifelines package . Additional  statistical tests were  performed in Python using SciPy package (Mann  Whitley, Fisher's exact test) and the R statistical  programming environment (Fisher's exact test). | Out of approximately 54,000 probe sets, we identified 258 probe sets as discriminative regarding metasta ses.136  of these were up-regulated  in metastasis positive  tumours; the remaining 122 were down-regulated . We used nCounter to measure the mRNA levels of the 193 genes in FFPE samples  corresponding to 144 of the 150 frozen tumour samples. our selection criteria were  high PPV and reasonable  sensitivity; a PPV of 0.66  substantially exceeds our  goal of a PPV >0.4, which is the metastasis risk for  patients with stage III tu  mours, for whom adjuvant chemotherapy is generally recommended. | we first measured the  expression levels of a large number of genes using  microarrays and RNA from fresh-frozen tumour  samples. We selected 193 genes, for which we  designed NanoString  nCounter assays that we applied to FFPE tumour  samples corresponding to the original frozen tumour samples. We also  considered miRNA  expression and mutations in selected cancer-related  genes. However, including  these data did not improve prediction. We then selected  19 of the 193 genes for  expression analysis with nCounter assays plus a  Random Forest voting  threshold, which together constitute the ColoMet19 predictor. We tested  ColoMet19 on an  independent set of FFPE tumour samples | In summary, we successfully designed and validated  ColoMet19, an expression- based predictor suitable for use with FFPE samples that estimates the propensity for metastasis of early-stage  low-risk MSS CRC patients. | ColoMet19 would need to be validated in prospective studies using the same  voting threshold on  independent sets of FFPE tumour samples from other centers  before translation to clinical practice, and tested in other ethnicities to determine its  breadth of applicability.  Similarly, ColoMet19's utility for right-sided tumours or  tumours in younger patients would need to be assessed. The adoption of this test  could then improve the  selection of early-stage CRC patients for adjuvant | Low |
| Manilich, E. A., et al | A novel data-driven prognostic model for staging of colorectal cancer(69) | 2011 | USA | The aim of this study was to develop a novel prognostic  model that captures  complex interplay among  clinical and histologic factors to predict survival of patients with colorectal cancer after a radical potentially curative  resection. | 2,505 colon cancer and  2,430 rectal cancer patients | Colorectal Cancer (CRC) | RSF analyses were used to evaluate the prognostic  significance of 17 variables. These included TNM  classification, location and size of tumor, resection  margins (defined as clear or involved), lymphovascular in vasion, differentiation,  number of lymph nodes  collected, number of positive lymph nodes, lymph node  ratio (LNR) defined as the  number of positive nodes  divided by the total number of lymph nodes collected, demographics (age, sex),  American Society of  Anesthesiologists (ASA)  class, year of surgery, and treatment approaches  (surgical, neoadjuvant, and adjuvant therapy; here  “neoadjuvant” refers to  chemotherapy given  preoperatively to reduce  tumor burden to facilitate or  enable surgery and  “adjuvant” refers to chemo therapy given  postoperatively). RSF  produced a forest of 1,000  random survival trees. Each of these bootstrap survival  trees was constructed from about two-thirds of the data; the remaining one-third (out of-bag) of the data was used to extract a Kaplan-Meier  function and generate  survival curves for each  patient. The RSF model also yielded a rank ordering of all variables based on VIMP,  which reflects how strongly each risk factor is  associated with disease-free survival | model development | external validation | Cox proportional hazard models. Random survival forest | The effect of Tumor-node- metastasis (TNM) and non- TNM factors such as  histologic grade, lymph node  ratio (number positive/  number re sected), type of operation, neoadjuvant and  adjuvant treatment,  American Society of  Anesthesiolo gists (ASA) class, and age in staging and prognosis were  evaluated. | An institutional review  board-approved colorectal  cancer registry is maintained by the Department of  Colorectal Surgery at the  Cleveland Clinic. A total of  2,505 colon cancer and  2,430 rectal cancer patients underwent radical potentially curable resections between 1969 and 2007 at a single  institution. Median  follow-up for the patients  was 64 months, with an inter quartile range from 35 to 113 months. The primary end  point was disease-free  survival. RSF use was  mentioned in use of Random forest colomn | To summarize, the novel  data-driven methodology for sur vival data identified  patterns of cancer  characteristics pre dictive of survival for patients with  resected colon and rec tal  cancer. The most important predictors selected were  LNR, which had higher  discriminatory power than  the number of positive  lymph nodes (increases in  LNR were associated with a worse disease-free survival for both colon and rectal  cancer), pT category, and  ASA class (colon can cer) or treatment type (rectal  cancer). Previous studies  have shown that LNR is a  powerful prognostic factor in solid cancer types including colorectal cancer. | A novel data-driven  methodology predicts the  survival times of patients  with colorectal cancer and  identifies patterns of cancer characteristics. The methods lead to stage groupings that could redefine the  composition of Tumor-node- metastasis (TNM) in a simple and orderly way. The higher predictive power of lymph  node ratio as compared with traditional pN lymph node  stage has specific  implications and may  address the important  question of accuracy of staging in patients when fewer than 12 nodes are identified in the resection specimen. | Low |
| Lu, T., et al. | Comparison of  Machine Learning and Logic  Regression  Algorithms for  Predicting Lymph  Node Metastasis in Patients with Gastric Cancer: A two-  Center Study(70) | 2024 | China | The study aimed to establish a model for predicting the  risk of lymph node  metastasis in gastric cancer patients using machine  learning (ML) and logistic  regression (LR) algorithms,  and to evaluate its predictive performance in clinical  practice | 369 | Gastric cancer (GC) | RF is an exten sion of the Decision tree (DT) method and functions as an  ensemble approach. It  generates multiple DTs, with the majority vote from these  trees determining the final class prediction of the  model. | model development | internal & external validation | decision tree, random forest,  support vector machine  (SVM), gradient boosting  machine (GBM), naive  Bayes, neural network, and Logistic regression (LR) | The inclusion criteria were  summarized as follows: (a)  patients with primary gastric  cancer who were initially  diag nosed in two hospitals and were treated with  concurrent surgery; (b)  confirmation of lymph node metastasis by imaging and pathology; (c) obtaining the maximum tumor diameter, nerve or vascular invasion, and the depth of tumor  invasion from the  postoperative pathology. Other indicators were  achieved from medical  records and preoperative blood tests; (d) antitumor ther apies, such as  radiotherapy and  chemotherapy were not per formed preoperatively; and  (e) the existence of complete clinical data. The exclusion  criteria were summarized as follows: (a) Combination with other malignant tumors; (b)  the presence of other  infectious diseases, blood system diseases,  autoimmune diseases, and other disease complications that might affect  inflammatory indicators  preoperatively; (c) currently receiving or having history of recently receiving anti  inflammatory or  immunosuppressive therapy; (d) Preoperative transfusion treatment; (e) severe liver  and kidney insufficiency; and (f) Incomplete clinical data.  The reporting of this study conforms to TRIPOD  guidelines | Data of a total of 369  patients who underwent radical gastrectomy from March 2016 to November  2019 were collected and  retrospectively analyzed as the training group. 7 ML and logistic models were  developed, including  decision tree, random forest, support vector machine  (SVM), gradient boosting  machine (GBM), naive  Bayes, neural network, and LR, in order to evaluate the occurrence of lymph node  metastasis in patients with  gastric cancer. The ML  model was established  following 10 cross-validation iterations within the training dataset, and subsequently, each model was assessed  using the test dataset. The model’s performance was  evaluated by comparing the area under the receiver  operating characteristic curve of each model. | Compared with the  traditional logistic model,  among the 7 ML algorithms, except for SVM, the other  models exhibited higher  accuracy and reliability, and the influences of various risk factors on the model were  more intuitive. | For the prediction of lymph node metastasis in gastric  cancer patients, the ML  algorithm out performed  traditional LR, and the GBM algorithm exhibited the most robust predictive capability. | Low |
| Lin, X., et al. | MRI-based  radiomics model for preoperative  prediction of  extramural venous invasion (EMVI) of rectal  adenocarcinoma(71) | 2024 | China | To assess EMVI  preoperatively through  radiomics technology, and use different algorithms  combined with clinical  factors to establish a variety of models in order to make the most accurate  judgments before surgery. | 212 | Extramural venous invasion (EMVI) in rectal  adenocarcinoma | the random forest was used aiming to predict EMVI  status from multi-  dimensional perspectives.  The values of AUC and ACC were used to compare the  diagnostic efficiency of each model and screen out the  optimal model for predicting EMVI status. | model validation | external validation | logistic regression (LR), random forest (RF), and support vector machine (SVM). The receiver  operating characteristic (ROC) curves were  performed to assess the  effectiveness of radiomics  models and the area under  the curve (AUC) values were calculated for quantification. | The inclusion criteria were as  follows: (i) pathologically confirmed as rectal  adenocarcinoma; (ii)  received MRI within two  weeks before resection; and (iii) completed clinical and  pathological data. The  exclusion criteria were  patients who received  antitumor treatment before MRI or had insufficient  imaging quality. The patients were randomly distributed to the training (n=148) and  validation datasets (n=64) at a proportion of 7:3. | A total of 212 patients with rectal adenocarcinoma  between September 2012  and July 2019 were included and distributed to training  and validation datasets.  Radiomics features were  extracted from pretreatment T2-weighted images.  Different prediction models (clinical model, logistic  regression [LR], random  forest [RF], support vector machine [SVM], clinical-LR model, clinical-RF model, and clinical-SVM model)  were constructed on the  basis of radiomics features and clinical factors,  respectively. The area under  the curve (AUC) and accuracy were used to  assess the predictive  efficacy of different models. Sensitivity, specificity,  positive predictive value (PPV), and negative  predictive value (NPV) were also calculated | The clinical-LR model  exhibited the best diagnostic efficiency with an AUC of  0.962 (95% confidence  interval [CI] = 0.936–0.988)  and 0.865 (95% CI = 0.770– 0.959), accuracy of 0.899  and 0.828, sensitivity of  0.867 and 0.818, specificity of 0.913 and 0.833, PPV of  0.813 and 0.720, and NPV of 0.940 and 0.897 for the  training and validation  datasets | The radiomics-based  prediction model is a  valuable tool in EMVI  detection and can assist  decision-making in clinical practice | High |
| Takamatsu, M., et al. | Prediction of early colorectal cancer metastasis by machine learning  using digital slide  images(72) | 2019 | Japan | Here we describe an LNM prediction algorithm for  submucosal invasive (T1) CRC based on machine learning. | 520 T1 CRCs, 397 cases  were included for further  image processing | Colorectal cancers (CRC) | We selected the random  forest classifier (RFC)  machine learning algorithm  to minimize the effect of  overfitting, a major cause for diminishing predictive ability for unknown data (we used  the random forest classifier  (RFC) on the morphologic  parameters of whole slide  images, which provided high predictive ability of LNM with the highest AUC of 0.938). | model development | internal validation | We performed cross  validation with repeated random subsampling. Almost all of the cases  (98.7%, 392/397) were  assign to the test dataset at least once, with 12 validation datasets. | deep submucosal invasion  (=1000 μm), positive  lymphatic invasion, positive venous invasion, poorly  differentiated clusters, and high-grade tumor budding  were risk factors for lymph node metastasis (LNM). The most important parameter  for predicting LNM was the Feret Y diameter. All but one  training dataset (92%)  showed that the Feret Y diameter was the most important parameter | Case recruitment. Image  processing. Data splitting  and predicting LNM with the machine learning and  conventional methods.  Scheme of data splitting in the initial and cross  validation datasets | Machine learning showed  better LNM predictive ability than the conventional  method on some datasets.  Cross validation revealed no significant difference  between the methods.  Machine learning resulted in  fewer false-negative cases than the conventional  method. | Machine learning on whole slide images is a potential alternative for determining treatment strategies for T1 CRC. | Moderate |

| Thavanesan, N., et al  i  f  t  i  f  f  f  f  t  i  t  f  t | Machine learning to predict curative multidisciplinary  team treatment  decisions in  oesophageal cancer(73) | 2023 | UK | to develop ML models able to predict curative OC MDT treatment decisions and  determine the relative  importance of underlying decision-critical variables. | 399 | Oesophageal cancer (OC) | used to train models  predicting OC MDT  treatment decisions: surgery (S), NACT + S or NACRT + S | model development | internal validaton | Established ML algorithms (Multinomial Logistic  regression (MLR), Random Forests (RF), Extreme  Gradient Boosting (XGB)) and Decision Tree (DT)) | Performance metrics  included Area Under the  Curve (AUC),Accuracy,  Kappa, LogLoss, F1 and Precision -Recall AUC.  Variable importance was calculated for each model | Data analyses were  conducted using RStudio.  The choice of final treatment pathway was assigned as  the outcome variable:  Surgery (S), (NACT + S), or (NACRT + S). Four  established ML algorithms were selected and  implemented via the “caret” package. All models were  developed using nested  cross-validation (CV) and  optimised for accuracy.  Variable importance was  derived for each algorithm to examine, quantify and rank overall importance a given  feature provided to the  final models. For meaningful statistical comparison of  Area Under the Receiver  Operator Characteristic  curve's (AUROCs) produced for each algorithm all  algorithms were further re- trained total of 10 times,  (now producing a total of 50 “outer-fold” models) | MLR outperformed RF, XGB and DT across performance metrics (mean AUC of 0.793 [±0.045] vs 0.757 [±0.068], 0.740 [±0.042], and 0.709  [±0.021] respectively).  Variable importance analysis identified age as a major  factor in the decision to offer surgery alone or NACT + S  across models (p < 0.05)./// 1- Predictive performance for each algorithm was  assessed on mean model  performance and  individualised outcome-class prediction. All algorithms  produced models which performed above random chance . 2- A significant difference was  demonstrated on Kruskal-  Wallis and Pairwise Wilcoxon Rank Sum test for all  classes. 3-The MLR model  highlighted cT stage as most important, but with more  salience attributed to co-  morbidities such as  connective tissue disease, lymphoma, leukaemia, and liver disease. Within tree-  based models (RF, XGB and DT) the single most  influential variable was age. | ML techniques can use  limited feature-sets to  predict curative UGI  multidisciplinary team (MDT) treatment decisions.  Explainable Artificial  Intelligence methods provide insight into decision-critical variables, highlighting under lying subconscious biases in cancer care decision-  making. Such models may allow prioritization of  caseload, improve efficiency, and offer data-driven  decision-assistance to MDTs in the future. | Low |
| --- | --- | --- | --- | --- | --- | --- | --- | --- | --- | --- | --- | --- | --- | --- | --- |
| Tian, H., et al. | Application of machine learning  algorithm in  predicting distant metastasis of T1 gastric cancer(74) | 2023 | China | The aim of this study was to develop and validate a  predictive model for DM in stage T1 GC using machine learning (ML) algorithms | 2698 | Gastric cancer (GC) | the RF model had the best prediction efcacy (AUC:  0.941, accuracy: 0.917,  recall: 0.841, specifcity:  0.927, F1-score: 0.877). Te test set was used to further validate the results, which showed that the RF model was the optimal prediction model for predicting DM in stage T1 GC, with the best predictive efcacy  (AUC=0.825). Te ability of the  RF model to accurately predict DM in stage T1 gastric cancer was also confrmed by an external validation set  (AUC=0.750).The RF seems to be one of the most widely used and accurate machine learning models in clinical  application research.  Increasing evidence has  reported that the random forest model is superior to other algorithms in dealing with data having a large  number of features and  highly nonlinear data,  probably because the RF  model uses more advanced classifcation decisions and  diferent weight ratios  compared to other  models21,22. Tis study  confrmed that the random forest prediction model can accurately predict the high- risk group with distant  metastasis in T1 patients,  which is conducive to further clinical examination for this  population to develop better diagnosis  and treatment strategies | model development | external validation | We applied seven ML  algorithms: logistic  regression, random forest  (RF), LASSO, support vector machine, k-Nearest  Neighbor, Naive Bayesian Model, Artifcial Neural  Network. | The AUC, sensitivity,  specifcity, F1-score and  accuracy were used to  evaluate and compare the predictive performance of the RF model with other  models. K-M curves were used to express diferences in survival prognosis for  each variable and  subvariable./// In the  research, we considered 11 variables totally which was  divided into three categories.  Population characteristic  variables include sex (Male, Female), age (<40, 40–60, 60–80, >80).  Clinicopathological variables include tumor size (<2 cm,  2-5 cm, >5 cm, NA),tumor location (Fundus, Body,  Antrum, Pylorus, Lesser curve, Greater curve,  Overlapping, NOS), grade  (Well, Moderate, Poorly,  Undiferenti ated, NA), M-  stage (M0, M1), N-stage (N0, N1, N2, N3) and T-stage  (T1a, T1b). | Patients with stage T1 GC from 2010 to 2017 were  screened from the public  Surveillance, Epidemiology and End Results (SEER)  database. We applied seven ML algorithms. we  performed a prognostic analysis of patients who developed distant  metastases. Independent  risk factors for prognosis  were analysed by univariate and multifactorial regression. | RF prediction model had the best prediction performance (AUC: 0.941, Accuracy:  0.917, Recall: 0.841,  Specifcity: 0.927, F1-score: 0.877). A recent study  showed that the probability of distant metastasis in  patients with stage T1 is  8.17%18. Terefore, it is  necessary to explore the risk factors and prognosis of  distant metastasis of T1  gastric cancer. Meaningfully, this is the frst study to  construct a model for  predicting distant metastasis of stage T1 gastric cancer  through machine learning  and analyse its survival and prognosis./// In our study  we found that N stage and T  stage were closely  associated with distant  metastasis. Interestingly, the rate of distant metastasis in patients with stage T1a was signifcantly higher than that in patients with stage T1b.  Tis may result from that the lymph node metastasis  occuring in submucosal patients frst, while  hematogenous metastasis occurs later in mucosal  patients during infltration into deeper layers.  According to Japanese  guidelines for the treatment of GC, patients with a tumor size>2 cm have a  signifcantly increased risk of metastasis and should  receive radical resection for clean removal | In conclusion, we  constructed and verifed a  prediction model of DM in  patients with T1 GC through ML algorithm. Te RF model has the best prediction  efciency and can accurately screen high-risk groups,  providing help for further  clinical metastasis  screening. Meanwhile, our study also found that  aggressive surgery and  adjuvant chemotherapy can improve the survival rate of patients with DM. | Low |
| Tian, Y., et al. | Prediction of  postoperative  infectious  complications in  elderly patients with colorectal cancer: a study based on  improved machine learning(75) | 2024 | China | We aimed to develop a  model for predicting  infectious complications after colorectal cancer  surgery in elderly patients  based on improved machine learning (ML) using  infammatory and nutritional indicators. | 512 | Colorectal cancer (CRC) | Categorical variables were  encoded into binary variable,  1 represents having an  ncident, 0 represents not having an incident. Gender was also encoded, 1  represents male, 0  represents female. Overftting may occur in the process of  model training, thus  destroying the performance of the model. Terefore, we rst perform single factor  analysis to flter out features hat are not statistically  signifcant, and then  ntroduce the recursive  eature elimination (RFE)  method of random forest.  This method first trains all  eatures, then recursively  removes the least important eatures, and selects the  eature set with the highest recall score. We further use he recursive feature  elimination (RFE) method to select features and rank the mportance of features. Te  univariate and RFE methods are used for feature selection o reduce 36 features to 10  eatures. These 10 features  were ASA, operation time,  diabetes, presence of stomy, umor location, NLR, PLR,  PNI, LCR, and LMR (P<0.05) | model development | internal validation | Linear Regression, Random Forest, Support Vector  Machine (SVM), BP Neural  Network (BP), Light Gradient Boosting Machine (LGBM), Extreme Gradient Boosting (XGBoost) and an improved moderately greedy XGBoost (MGA-XGBoost) model. | The performance of the  seven models was evaluated by area under the receiver  operator characteristic  curve, accuracy (ACC),  precision, recall, and F1-  score of the validation set. . Postoperative infectious  complications were notably associated with 10 items  features: American Society of Anesthesiologists scores (ASA), operation time,  diabetes, presence of stomy, tumor location, NLR, PLR,  PNI, LCR, and LMR | We employed mean  imputation, which imputed  missing value with the mean of each feature, to fll in  missing val ues. Patients  with postoperative infectious complications accounted for 24% (n=125), 70% (n=358)  in the train ing sets, and 30% (n=154) in the  validation set. There were  295 male patients (57.62%) and 217 female patients  (42.38%). To better  understand the data  characteristics of the model, the patients were divided  into an infected group and a non-infected group  according to the training set and validation set, and then the data were analyzed by  single factor analysis. To  better see whether there is a correlation between risk  factors, this paper analyzes the correlation of statisti  cally signifcant indicators in RFE methods. Te results  showed that there was a  high correlation between PNI and LMR (0.71) NLR and  PLR (0.35). To evaluate the  predictive efect of seven  machine learning models on postoperative infectious  complications in elderly  patients. The results showed that the AUC value of the  MGA-XGBoost prediction model was the highest  (0.862), and Linear  Regression, SVM, and BP all showed general predictive  ability | The results of this study  showed that with preop  erative PNI<48.48, the  incidence of postoperative infec tious complications increased. Terefore, this  study showed that  infammatory response and nutritional indi cators were signifcantly associated with postoperative infection. At the same time, this study  determined five  comprehensive infammatory  indicators related to post operative infection of  colorectal cancer by single factor analysis and RFE  method. In this study the importance of internal  features in the verifcation data set of the MGA-  XGBoost prediction model  with the highest accuracy is visually displayed by three  methods of cover, weight  and gain. LCR had the  highest correlation with  colorectal cancer recurrence  and was a more reliable biomarker. It may be  because preoperative  CRP is associated with  lymphopenia and T  lymphocyte reaction cell damage in patients with colorectal cancer, and  lymphocytes play a key role  in the host’s cytotoxic  immune response to tumors, which impairs cell mediated immunity in patients with  colorectal cancer. Mik et al. found that a total operation time of more than 180  minutes increases the risk of surgical site infection in deep incisions and organ spaces.  At the same time, the longer the operation time, the  greater the possible trauma and the more blood loss,  which further reduces the patient ‘s resistance and makes the patient more  prone to infection | In summary, our study  demonstrates for the frst  time that the MGA-XGBoost model with 10 risk factors  can predict postoperative  infectious complications in elderly CRC patients. At the same time, combining risk prediction with feature  importance analysis allows  clinicians to assess  postoperative risks and  potentially modifable drivers | Moderate |
| Tibermacine, H., et al. | Radiomics modelling in rectal cancer to  predict disease-free survival: evaluation of different  approaches(76) | 2021 | France | The aim of this study was to assess and compare  different radiomics  approaches over qualitative evaluation to predict  disease-free survival (DFS) in  patients with locally  advanced rectal cancer treated with neoadjuvant therapy. | training set: 98 patients test  set: 48 patients. | Rectal cancer | Random forest classifiers were built after tuning  hyperpara meter values  through an iterativegrid  search procedure. Tuned hyperparameters included the number of trees in the forest, the number of  predictors randomly  selected for consideration at each tree node, the  maximum depth of the  forest, and the minimum  samples split. The scaled  Brier score was used as the primary performance metric. | model validation | external validation | Random forest classifiers | radiomics models were  compared with qualitative parameters, including  sphincter invasion,  extramural vascular invasion  as determined by MRI  (mrEMVI) at baseline, and tumour regression grade  evaluated by MRI (mrTRG) after chemoradiotherapy (CRT). Hand-crafted  radiomic features were  extracted automatically  from manually segmented  ROIs and BBs, including  tumour shape and size,  intensity statistics, and  texture (including the grey  level co-occurrence matrix, grey level run-length matrix, and grey level size zone  matrix). The whole radiomic feature set also con tained higher-order statistical  features, including the  intensity and texture features derived from the images  processed with eight types  of filter (wavelet, Laplacian  of Gaussian, square, square root, logarithm, exponential, gradient, LBP 2D for 2D mod els or LBP 3D for 3D  models). Features were  computed using  PyRadiomics, an open-  source radiomics package | All patients previously  enrolled in the original  GRECCAR4 (Groupe de  REcherche en Chirurgie sur le CAncer du Rectum)  prospective II multicentre randomized study  (GRECCAR4;NCT01333709)  22 were included in the  present analysis. The  training cohort for the  present study included  patients who underwent  both baseline and post-CRT MRI. In addition, an  independent cohort served  as the test set23. All patients  in both cohorts had  neoadjuvant CRT. Training- set images were obtained  using a phased-array body coil at 1.5 or 3 T. Regions of  interest (ROIs) were  segmented manually by a  radiolo gist with 3 years of  experience in rectal imaging, who was blinded to clinical  data. Manual segmentation (MS) was performed using free open-source software. Statistical analyses, feature selection, and model  development were  undertaken using python in Anaconda3 platform with  Scikit learn and Matplotlib packages. | This study found that  different radiomics models were able to predict the risk of recurrence from MRI in  patients with LARC at  baseline and after CRT, with  the 3D MS model  outperforming qualitative analysis in the training set and all models showing  similar results on  independent testing.// In the present study, no difference was found between the seg mentation approach used.  To date, nearly all studies  published on rectal cancer radiomics have used MS for  tumour segmentation.  Although manual delineation is a relatively simple process when carried out by experts, it is a highly subjective  approach that requires  maximal human input and  time to process. As such, in an attempt to make the  process of radiomics easier, the 2D bounding box (BB)  segmentation method was investigated. The  advantages of the BB  approach include time  efficiency, possible  increased stan dardization/  reproducibility, and  incorporation of the  peritumoral environment, which may be valuable for the model’s perfor mance with minimal human input | Radiomics models can  predict DFS in patients with locally advanced rectal  cancer. | Moderate |
| Tsuji, S., et al. | Potential responders to FOLFOX therapy for colorectal cancer by Random Forests analysis(77) | 2012 | Japan | In the present study, we  used the RF algorithm to  identify classifier genes that are able to predict  responders to FOLFOX therapy for unresectable CRC | 83 | Colorectal cancer (CRC) | Using Random Forests (RF) algorithm in the training set, predictor genes for FOLFOX therapy were identified,  which were applied to test samples and sensitivity,  specificity, and out-of-bag classification accuracy were calculated. | model development | internal validation | RF algorithm & Out-of-bag classification Using these 14 classifier genes (SMURF2,  MBTD1, AP3M2, RNF141, NPEPPS, BPTF, FAM73A, APPBP2, AMZ2P1,  SRGAP1, NMT1, CSPP1,  EIF1, and CEP290)/// In the present study, two-way  hierarchical clustering  analysis failed to significantly separate responders and  non-responders (data not shown). | Random  Random Forests is not only a learning algorithm for  building a class prediction  model, but it calculates  useful characteristics about samples, such as outlier  measurements for each  sample or proximity matrixes representing the similarity  between all pairs of  samples. For subsequent analyses, we used the  proximities  obtained by a second RF analysis (Figure 1) : Age (years), Gender (male/  female), CEA (ng/ml),CA19-9 (U/ml ), Differentiation grade (well/mod/por), Primary  lesion (rt/lt), Metastatic  lesion (liver/lung/bone/  peritoneum) | A total of 83 patients with  unresectable CRC  undergoing FOLFOX therapy from April 2007 to December  2010 were recruited in this  study. Samples were divided (approximately 2 : 1 ratio)  into training and test sets.  As a result, 54 of 83 samples obtained in the first half of  this period were selected for the training set, and the  remaining 29 samples in the latter half were selected as  the test set. All patients were treated with mFOLFOX6,  After four cycles of  mFOLFOX6 therapy, all  lesions were assessed by  computed tomography, and classified as CR (disap  pearance of all target  lesions), partial response  (PR, at least a 10% decrease in the sum of the longest  diameter of target lesions), progressive disease (PD, at least a 10% increase in the sum of the longest diameter of target lesions), and stable disease (SD, neither  sufficient shrinkage to  qualify for PR nor sufficient increase to qualify for PD), according to the Response Evaluation Criteria in Solid Tumors (Therasse et al,  2000), with minor  modification. Before further  statistical analysis, we  normalised and filtered the raw data. The Affymetrix  Power Tools were used to summarise the probe  intensity of the CEL file by apt-probeset-summarise command with plier mm- skech option. In the RF  algorithm, the genes that  confer resistance to FOLFOX therapy were ranked by  frequency of occurrence in each out-of bag cross-  validation, which was  repeated at 200 000 times. The top ranking genes were  selected as predictors to maximise out-of-bag  classification accuracy. | In the training set, 22 of 27 responders (81.4%  sensitivity) and 23 of 27 non- responders (85.1%  specificity) were correctly classified. To improve the prediction model, we  removed the outliers  determined by RF, and the model could correctly  classify 21 of 23 responders (91.3%) and 22 of 23 non-  responders (95.6%) in the training set, and 80.0%  sensitivity and 92.8%  specificity, with an accuracy of 69.2% in 29 independent test samples. | Random Forests on gene-  expression data for CRC  patients was effectively able to stratify responders to  FOLFOX therapy with high accuracy, and use of  pharmacogenomics in  anticancer therapy is the first step in planning  personalised therapy | Low |

| Tian, H., et al. | Application of  Machine Learning  Algorithms to Predict Lymph Node  Metastasis in Early Gastric Cancer(78) | 2021 | China | This study aimed to  establish the best early  gastric cancer lymph node metastasis (LNM) prediction model through machine  learning (ML) to better guide clinical  diagnosis and treatment decisions. | 2294 | Gastric cancer (GC) | we applied 7 ML algorithms and combined them with  patient pathological  information to develop the best prediction model for early gastric cancer lymph node metastasis | model development | external & internal validation | Generalized linear model (GLM), RPART, random  forest (RF), gradient  boosting machine (GBM), support vector machine (SVM), regularized dual  averaging (RDA), and the neural network (NNET) | we used the AUROC, F1-  score value, sensitivity, and specificity to evaluate the  performance of the model./// First, univariate analysis  showed that race, tumour  size, tumour grade, tumour tissue type, tumour location,  and depth of tumour  invasion were related to  LNM, and the results were  statistically significant (P <  0.05). We conducted binary logistic regression analysis  on factors P < 0.1 (age, race, tumour size, tumour grade,  tumour tissue type, tumour site, and depth of invasion). Tumour size, tumour grade and infiltration depth were independent risk factors for LNM in patients with EGC.  The external validation set also confirmed that tumour size, tumour grade, and  depth of invasion were risk factors for lymph node  metastasis (P < 0.05) | The data were obtained from the Surveillance,  Epidemiology and End  Results (SEER) database of the National Cancer  Institute. The inclusion  criteria were as follows: (1)  Patients undergoing surgical treatment; (2) A pathological diagnosis of early gastric  cancer; (3) Complete survival information. Missing values were detected using the  mice package and filled with predictive mean matching.  The database patients were randomly divided into a  training set and a test set at a ratio of 8:2, and hospital  patients were used as the  external verification set. The training set was used for  model development, and the test set was used for  evaluation  and verification. Considering that the proportion of  patients with LNM was too low, we used the ROSE  package to balance the  training set. Since then, 7  types of ML algorithms have been established by the  training set | The parameters of the  training set were adjusted to balance the model and avoid overfitting the model. After  balancing the parameters of the training set, we found  that the GBM model had the best predictive ability, with  AUCROC = 0.825. external  validation set also verifies  the applicability of the RDA prediction model (AUCROC= 0.73). Therefore, we believe that the RDA model is robust in predicting LNM | Tumour size, tumour grade,  and depth of tumour  invasion were independent risk factors for early gastric cancer LNM. ML predicted LNM risk more accurately,  and the RDA model had the best predictive performance  and could better guide  clinical diagnosis and  treatment decisions. | Low |
| --- | --- | --- | --- | --- | --- | --- | --- | --- | --- | --- | --- | --- | --- | --- | --- |
| Tabari, A., et al. | Quantitative  peritumoral magnetic resonance imaging  fingerprinting  improves machine  learning-based  prediction of overall survival in colorectal cancer(79) | 2024 | USA | To investigate magnetic  resonance imaging (MRI)-  based peritumoral texture  features as prognostic  indicators of survival in  patients with colorectal liver metastasis (CRLM) | 48 | colorectal cancer (CRC)  colorectal liver metastasis (CRLM). | A random forest-based  machine learning model was applied to predict the  outcome. The area under the  curve (AUC) for using  radiomic features using the random forest algorithm was  generated by receiver  operating characteristic (ROC) analysis. | model development | internal validation | random forest-based  machine learning model ///  Parameter optimization was  performed using 3-fold  cross-validation to create a  generalized model and  minimize the risk of  overﬁtting. To ensure the  generalizability of our  ﬁndings, future work should  involve an external validation  study across multiple  centers. | All patients met the following  criteria: (a) biopsy proven  colorectal adenocarcinoma;  (b) presence of CRLM; (c)  T1-weighted post-contrast  magnetic resonance imaging  (MRI) within 3 months prior  to preoperative FOLFIRI or  FOLFOX-based  chemotherapy. Patient age,  sex, lesion size, location of  primary tumor, tumor Ki-ras2  Kirsten rat sarcoma viral  oncogene homolog (KRAS)  status, tumor microsatellite  instability (MSI), stage of  colon cancer, radiotherapy  status, chemotherapy  regimen, and follow-up  duration were collected from  the medical records. All-  cause mortality was the  assessed outcome.\\\ MRI-  based radiomics features :  A total of 112 radiomic  features were derived from a  10 mm region surrounding  each segmented tumor.  Univariate logistic regression  and Wald test results  showed 42 features  including: 8 ﬁrst order, 5  GLDM, 5 GLRLM, 5 GLSZM,  2 NGTDM, and 17 GLCM to  be independently correlated  with the prediction of  patients’ OS (all P < 0.05 on  Wald test). | In this institutional review board (IRB) approved  retrospective study, forty- eight patients with biopsy proven stage IV CRC with liver metastasis who  underwent treatment with front-line standard  chemotherapy protocols  were identified, between  January 2007 and December  2015. The treatment of all  patients was carried out by a team of oncologists at the  Massachusetts General  Hospital. A contrast-  enhanced abdominal MRI  was acquired within 3  months before starting  treatment. All the liver MRI  exams were done on either a 1.5T MRI scanner or a 3T  magnet with a body coil  positioned over the  abdomen. The Digital  Imaging and  Communications in Medicine (DICOM) images were  reviewed by radiologists with expertise in liver imaging  and blinded to the clinical  findings to identify the  CRLM. The window levels were adjusted to optimize visualization of the liver  mass. Manual delineations included a 10 mm region surrounding each  segmented tumor and semi- automatic volumetric  segmentation methods were applied using 3D slicer. A  multiparametric vector was derived from each lesion. All images were normalized  prior to extracting  quantitative features. The  dataset was partitioned into training and testing sets at a ratio of 4:1. Recursive  feature elimination was  applied to select and test the effectiveness of different  numbers of radiomic  features. The survival  prediction score was derived from the Cox model with  Kaplan-Meier survival curve, for each patient. The  contribution of each texture feature to OS prediction was assessed using univariate  logistic regression analysis. | The median lesion size was  25.73 mm (range 8.5–103.8 mm). Microsatellite instability was low in 40.4% (38/94) of tumors, with Ki-ras2 Kirsten rat sarcoma viral oncogene  homolog (KRAS) mutation  detected in 68 out of 94  (72%) tumors. The mean  survival was 35 months ± 21 months, and local disease  progression was observed in 35.5% of patients. Univariate regression analysis identified  42 texture features [8 first  order, 5 gray level  dependence matrix (GLDM),  5 gray level run time length matrix (GLRLM), 5 gray level size zone matrix (GLSZM), 2 neighboring gray tone  difference matrix (NGTDM), and 17 gray level co  occurrence matrix (GLCM)] independently associated with metastatic disease  progression (P < 0.03). The random forest model  achieved an area under the curve (AUC) of 0.88. | In conclusion, machine  learning-based predictive  models that incorporate  radiomic features from the  peritumoral region may  enhance the identification of lesions with better OS prior to initiating treatment. This information could be  valuable for treatment  optimization in patients with CRLM. In the next phase of this study, the goal is to  conduct an external  validation in a multicenter setting. | Low |
| Alinia, Sh et al | Predicting mortality and recurrence in  colorectal cancer:  Comparative  assessment of  predictive models(80) | 2024 | Iran | forecasting early  recurrence and mortality subsequent to curative surgical procedures in a cohort of 284 patients  undergoing resection for colorectal cancer. | 284 individuals | colorectal cancer | prediction of mortality and recurrence | Model validation | internal validation | Cox proportional hazards, Machine learning models, Deep Learning Neural  Network | gender, BMI, age | In this retrospective cohort  study, 284 CRC patients  who underwent surgical  resection between, 2001 and  2017 were analyzed for  demographics, clinical  factors, recurrence, and  mortality. Data were  categorized for analysis, and various predictive models  were assessed for their  performance in predicting death and recurrence using R and Python software | For both death and  recurrence random Forests showed perfect sensitivity (100%), but low specificity (0%) and poor overall  accuracy (50%). | This study has demonstrated  that the utilization of ML methods, specifically  mboost and Gradient  Boosting models, | Moderate |
| Chen, Q et al | A four-lncRNA  signature for  predicting prognosis of recurrence  patients with gastric cancer(81) | 2021 | China | to develop a multi-long  noncoding RNA (lncRNA)  signature for the prediction of gastric cancer (GC) based on differential gene  expression between  recurrence and  nonrecurrence patients. | RNA seq data of 407 GC, mRNA seq of 477 samples | gastric cancer (GC) | The Random Forest–Out-of- Bag (RF–OOB) algorithm is  used to identify recurrence- related feature lncRNAs from the preselected differentially expressed lncRNAs. | Model development | external validation | R software (version 3.4.1), SVM–RFE and RF–OOB  algorithms, Cox | gender, four-lncRNA  risk score model status | We obtained RNA  sequencing data (including mRNA and  lncRNA).Two data sets  including GSE26253 and GSE62254 were chosen. there were no specific  information of  targeted drug molecules. Meanwhile, the detailed clinical  characteristics of the  patients were analyzed.In  order to exclude the effect of  therapeutic  schedule on the screening of the present lncRNAs, corre  lation analysis was used to eliminate the doubt in this  aspect, and the results  showed that four important lncRNAs  were not related to  radiotherapy, chemotherapy, and  targeted therapy. | RF–OOB algorithm : These results suggested that the seven-feature lncRNAs  were more indicative of  recurrence than the eight- feature  lncRNAs and were thus used in further analysis | In conclusion, we generated  a recurrence-related  four-lncRNA signature  predictive of individual  mortality  risk of DFS in GC patients. This study  suggested potential  prognostic biomarkers and therapeutic  targets for recurrence GC  and provided novel insights into  the underlying mechanisms of GC progression. | Low |
| Chen, T et al | Identification of three metabolic subtypes in gastric cancer and the construction of a metabolic pathway- based risk model  that predicts the  overall survival of GC patients(82) | 2023 | China | to identify GC subtypes and genes related to prognosis, based on changes in the  activity of core metabolic pathways in GC tumor  samples. | 865 GC samples and  patient  information | Gastric cancer (GC) | To select the most significant DEGs from the initial, larger set of DEGs. | Model development | internal validation | Random forest,univariate Cox regression, Lasso  regression | Age,Sex, Race, Tumor grade | We collected the original  gene expression profiling  data of tumorand adjacent  normal tissues, as well as  the clinical characteristics of Gc cohorts., 865 GC  samples and patient  information were included in the analysis. This included  patients from  the GSE84437 and  GSE26253 cohorts. We used  the 865 GEO GC  studycohort with 865 patient information for validation of  our prognostic  model. The annotation  results were divided into  epithelial cells,B cells, T cells and other cell types. Using  the scMetabolism  package,each cell was  scored using the VISION  algorithm to derive activity  scores for different cell types  in different metabolic  pathways. | the ROC curve (AUCs) were 0.71  (at 1 year), 0.77 (at 3 years), and 0.73 (at 5 years). | In conclusion, our work identified  significant GC prognosis-  related metabolic pathways in different GC subtypes and provided new insights into  GC-subtype prognostic  assessment. | Low |
| Daye,D et al | Quantitative tumor heterogeneity MRI profiling improves machine learning– based  prognostication in patients with  metastatic colon cancer(83) | 2020 | USA | to assess the role of  quantitative MRI-based  measures of intra-tumor  heterogeneity as predictors of survival in patients with  metastatic colorectal cancer. | 55 patients | colorectal cancer | Random forest-based  machine was used to predict patient survival outcomes  during the study period. | model development | internal validation | logistic regression, The  Kaplan-Meier survival  analysis, random forest, Python 3.8. | Age,Sex, KRAS status,  Microsatellite instability  (MSI), Colon cancer  site,Extent of metastatic disease, Chemotherapy regimen | In this IRB-approved  retrospective study, we  identified 55 patients with stage 4 colon cancer with  known hepatic  metastasis on MRI. Ninety- four metastatic hepatic  lesions were identified on  post-contrast images and  manually volumetrically  segmented. A heterogeneity phenotype vector was  extracted from each lesion.  Univariate regression  analysis was used to assess the contribution of 110  extracted features to survival prediction. A random forest– based machine learning  technique was applied  to the feature vector and to the standard prognostic  clinical and pathologic  variables. The dataset was divided into a training and test set at a ratio of 4:1.  ROC analysis and confusion matrix analysis were used to  assess classification  performance. | A random forest–based  model of the radiomics  features applied to the test ing set for patient survival prediction revealed an area under the curve  (AUC) of 0.93. A model that combines both clinical/  pathology features  with radiomics features for survival prediction (AUC = 0.94) has im proved  performance compared to a model that only include  clinical/pathology features (AUC = 0.83). | MRI-based texture features are associated with patient outcomes and improve the performance of standard  clinicaland pathological  variables for predicting  patient survival in metastatic colorectal cancer. | Low |
| Du, H et al | Identification and  Comprehensive  Analysis of FREM2 Mutation as a  Potential Prognostic Biomarker in  Colorectal Cancer(84) | 2022 | China | to identify genes that were  mutated in colorectal cancer (CRC) and to explore their  biological effects and  prognostic valueinCRC  patients. | RNA sequencing data  (count value) of 399 samples | colorectal cancer (CRC) | The random forest model was used to build a  predictive model for FREM2 mutation status based on  gene expression data in COAD patients. | Model development | internal validation | Cox regression analysis, RF method, Kaplan–Meier (K–M) survival curve  analysis and time-dependent ROC | age, gender, tumor stage, and risk score of COAD  patients with  FREM2 mutations | We performed somatic  mutation analysis using data sets  from The Cancer Genome Atlas and International  Cancer Genome  Consortium, and identified that FREM2 had the highest mutation frequency in  patients with colon  adenocarcinoma  (COAD). COAD patients  were divided into FREM2- mutated type (n = 36) and FREM2-wild type  (n = 278), and a Kaplan- Meier survival curve was generated to perform  prognostic analysis. A  FREM2-mutation prognosis model was constructed  using random forest method, and the  performance of the model  was evaluated using receiver operating characteristic  curve. | The efficacy of the model in 36 FREM2-mutant samples was  83.9% | In conclusion, CRC  patients had a high level of FREM2 mutations  associated with a worse  prognosis, which  indicated that FREM2  mutations may be potential prognostic markers in CRC. | Low |
| Guan, X et al | Computed  Tomography-Based Deep Learning  Nomogram Can  Accurately Predict Lymph Node  Metastasis in Gastric Cancer(85) | 2023 | China | To evaluate and verify the  predictive performance of  computed tomography deep learning on the presurgical  evaluation of lymph node metastasis in patients with gastric cancer. | 347 patients | Gastric cancer | The random forest is utilized as one of the classifiers to  build and evaluate  classification models based on selected features from  deep learning and radiomics data. | Model development | internal validation | ResNet50-RF model, The  classifcation models on the basis of RF or SVM. | Age, gender, Tumor  location,Tumor morphology, Laboratory tests, median  (IQR),CEA level, CA742 level, CT-reported LN status, | 347 patients were  retrospectively selected  (training cohort: 242, test  cohort: 105). The enhanced computed tomog raphy  arterial phase images of  gastric cancer were used for lesion segmentation,  radiomics and deep learning feature extrac tion. Three  methods were used for  feature selection. Support  vector machine (SVM) or  random forest (RF) was used to build  models. The classifcation  performance of the models  was evaluated using the area under the receiver operating characteristic  curve (AUC). We also  established a nomogram that included clinical  predictors. | ResNet50-RF model(Its  AUC, accuracy, sensitivity and specifcity were  0.9803, 0.9810, 0.9839,  0.9767, respectively), The  classifcation models on the basis of RF or SVM ( Its  AUC, accuracy, sensitivity and specifcity were 0.9606, 0.9619,  0.9677,0.9535,respectively)T he analysis results showed  that the ResNet50-RF model had the best classifcation  performance. | The computed tomography- based deep learning  nomogram can accurately and efectively evaluate  lymph node metastasis in  patients with gastric cancer before surgery | Moderate |

| Guan, Xu et al | An easy-to-use  artificial intelligence preoperative lymph node metastasis  predictor (LN-  MASTER) in rectal cancer based on a  privacy-preserving computing platform: multicenter  retrospective cohort study(86) | 2023 | China | use machine learning (ML) models to predict the LNM status before surgery based on a privacy-preserving  computing platform (PPCP) | 6578 patients | Rectal cancer (RC) | for predicting LNM status in RC patients. | model development | internal validation | Logistic Regression  (LR),SVM,Random Forest, Extreme Gradient Boosting | sex, age, BMI, Comorbidity, Distance from lower edge of tumor to anus, CEA,  CA19-9,Histology, Vascular/ lymphatic vessel invasion, T stage, Lymph node  metastasis | A total of 6578 RC patients were enrolled in this study. ML models, including  logistic regression, support  vector machine, extreme  gradient boosting (XGB), and random forest, were used to establish the prediction  models. The areas under  the receiver operating  characteristic curves (AUCs) were calculated to compare  the accuracy of the ML  models with the US  guidelines and clinical  diagnosis of LNM. Last, model establishment and  validation were performed in  the PPCP without the  exchange of raw data  among different institutions | randomforest (AUC, 0.82; 95% CI, 0.81–0.84;  P=0.189),XGB[AUC, 0.84;  95% confidence interval (CI), 0.83–0.86], LR (AUC, 0.76;  95% CI, 0.74–0.78; P=  0.004), SVM  models (AUC, 0.79; 95% CI, 0.78–0.81; P=0.024) | The proposed easy-to-use model showed good  performance for LNM  prediction, and the web tool can help  clinicians make treatment-  based decisions for patients with RC. Furthermore, PPCP enables state-of-the-art  model  development despite the  limited local data availability. | Moderate |
| --- | --- | --- | --- | --- | --- | --- | --- | --- | --- | --- | --- | --- | --- | --- | --- |
| Huang, Ch et al | Clinical Significance of Serum CA125,  CA19-9, CA72-4,  and Fibrinogen-to- Lymphocyte Ratio in Gastric Cancer With Peritoneal  Dissemination(87) | 2019 | USA | to extract the important risk factors of PD in GC | 391 patients | gastric cancer (GC) | the RF algorithm is crucial for understanding the  relative importance of  different risk factors and enhancing the predictive models for PD in GC. | Model development | internal validation | random forest, logistic  regression, nomogram  models, | age, sex, BMI,  indicators(NLR,PLR,ALR,FL R, Hb, CEA, CA19-9, CA125, CA72-4) | : The clinical data of 391  patients with GC were  collected, including 86 cases of PD. Then, 1:3 matching  was performed by  propensity score matching (PSM), and the  clinical data of the matched  344 patients were analyzed by univariate and  multivariate  conditional logistic  regression. Classification tree analysis was used to  obtain the decision  rules and a random forest  algorithm to extract the  important risk factors of PD in GC. A  nomogram model for risk  assessment of PD in GC  was established by using the rms  package of R software. | The area under the curve  (AUC) of CA125 was 0.820, the 95% CI was 0.764–  0.876, the sensitivity was 79.1%, and the  specificity was 84.9%. The AUC of CA72-4 was 0.717, the 95% CI  was 0.649–0.785, the  sensitivity was 57%, and the specificity was  86.4%. The AUC of CA19-9 was 0.684, the 95% CI was 0.615–  0.753, the sensitivity was  57%, and the specificity was 79.8%.  The AUC of FLR was 0.653, the 95% CI was 0.586–  0.720, the  sensitivity was 65.1%, and the specificity was 65.5% | CA125 > 17.3 U/ml, CA19-9 > 27.315 U/ml, and FLR >  2.555 were the  risk factors for GC with PD.  The decision rules and  nomogram model  constructed by  CA125, CA19-9, CA72-4,  and FLR can correctly  predict the risk of PD in GC. | Low |
| Jin, M et al | Fusobacteria  alterations are  associated with  colorectal cancer  liver metastasis and a poor prognosis(88) | 2024 | China | 16S RNA sequencing  technology was employed, in order to examine the gut microbial richness and  composition in patients with CRC with LM or NLM. | 126 patients | Liver metastasis in colorectal cancer (CRC) | RF as classification model : Random forest algorithm  was used to identify  bacterial species that could  distinguish  the LM status. | Model validation | internal validation | Random forest classification model, Bioinformatics  analysis, | age, sex , Tumor location,  Differentiation, AJCC stage, | In the present study,  high-throughput  16S RNA sequencing  technology was employed, in order  to examine the gut microbial richness and composition in patients with CRC with LM or NLM. A discovery cohort (cohort 2; LM=18; NLM=36) and a validation cohort  (cohort 3;  LM=13; NLM=41) were  established using fresh  feces. In addi-  tion, primary carcinoma tissue samples were also analyzed  (LM=8 and NLM=10) as a supplementary discovery cohort | In the discovery cohort of fecal samples, a relative high accuracy of  Actinobacteria (AUC=0.603), Bacteroidetes  (AUC=0.611), Firmicutes  (AUC=0.650), Fusobacterium (AUC=0.898) and  Proteobacteria (AUC=0.505) in predicting  LM was demonstrated (Fig. 6C and E). Similarly, in the validation cohort of fecal  samples, a relative high  accuracy  of Actinobacteria  (AUC=0.612), Bacteroidetes (AUC=0.557),  Firmicutes (AUC=0.623),  Fusobacterium (AUC=0.893) and  Proteobacteria (AUC=0.538)  in predicting LM was  demonstrated. | On the whole, the find-  ings of the present study  demonstrated that CRC with LM  and NLM exhibit distinct gut microbiota characteristics.  Fusobacteria detection thus has potential for use in  predicting  LM and a poor prognosis of patients with CRC. | Moderate |
| Kasai, A et al | A novel CT-based radiomics  model for predicting  response and  prognosis  of  chemoradiotherapy in esophageal squamous cell  carcinoma(89) | 2024 | Japan | develop and validate a novel CT-based radiomics model  for the prediction of the  response to CRT as well as the  prognosis of ESCC patients afer CRT | 50 patients | esophageal  squamous cell carcinoma (ESCC) | to predict prognosis | Both | internal validation | g Random Forest (RF)  model, Naive Bayes (NB) model, Ridge Regression (RR) model,  Artifcial Neural Network  (ANN) model, and Support  Vector Machine (SVM) model | Lymph node metastasis, age, gender, Serum SCC, Tumor location, | First, we extracted a total of 476 radiomics features from three-dimensional CT  images of cancer lesions in training cohort, selected 110 features associated with the CRT response by ROC  analysis (AUC≥ 0.7) and identifed 12 independent features, excluding  correlated features by  Pearson’s correlation  analysis (r≥ 0.7). Based on the 12 features, we  constructed 5 prediction  models of diferent machine  learning algorithms (Random Forest (RF), Ridge  Regression, Naive Bayes,  Support Vector Machine,  and Artifcial Neural Network  models). Among those, the RF model showed the  highest AUC in the training cohort (0.99 [95%CI 0.86– 1.00]) as well as in the  validation cohort (0.92  [95%CI 0.71–0.99]) to  predict the CRT response. Additionally, Kaplan-Meyer analysis of the validation  cohort and all the patient  data showed signifcantly  longer progression-free and overall survival in the high | Te RF model showed the  highest AUC (0.92 [95%CI  0.71–0.99]), which was  signifcantly higher compared with ANN and SVM by  DeLong’s test | In conclusion, we have  developed a CT-based  radiomics model using AI, which may have the  potential to predict the CRT response as well as the  prognosis for ESCC patients with non-invasiveness and  cost-efectiveness. | High |
| Keshtvarz Hesam Abadi, A et al | Comparison of  random forest and logistic regression methods in  predicting mortality in colorectal cancer patients and its  related factors(90) | 2019 | Iran | The purpose of this study was to predict the mortality rate of colorectal cancer in  Iranian patients and  determine the effective  factors on the mortality of patients with colorectal  cancer using random forest and logistic regression  methods. | 304 patients | colorectal cancer | It was used to predict  colorectal cancer patients and determine the  associated risk factors. | Model Development | external validation | random forest, logistic  regression. | Cancer stage, patient age, age at diagnosis, immune evasion mechanism, and tumor differentiation grade (Differentiation). | Data from 304 patients with colorectal cancer registry  from the Gastroenterology and Liver Research Center of Shahid Beheshti  University of Medical  Sciences during the years  2009 to 2014 were used as a retrospective study. Data  analysis was performed  using random forest and  logistic regression methods. To analyze the data, R  software version 3.4.3 was considered | The area under the curve  (AUC) for logistic regression and random forest methods are 80% and 98%,  respectively, indicating  better performance for the random forest method. | Variables such as Cancer  stage, age of diagnosis,  patient’s age, HLA, and  degree of differentiation are considered as the most  important factors affecting mortality in colorectal  cancer, that the patients’  longevity can be increased with the early diagnosis of cancer and screening  programs. | Low |
| Kim, J et al | Clinical scoring  system for the  prediction of survival of patients with advanced gastric cancer(91) | 2020 | Korea | to construct and validate a  risk scoring system  based on easily obtained clinicopathological and  labora tory parameters to predict the median overall survival and  probability for 1year survival in patients with advanced  gastric cancer who initiated first-line chemotherapy with a combination of a  fluoropyrimidine and a  platinum  agent according to the risk score | 1733 patients | gastric cancer | Objective and Data  Preparation-Data Splitting- Variable Selection Using  Random Forest-Integration into Cox Regression Model-  Model Validation and  Performance-Additional  Analyses-Conclusion and Future Directions.  Random Forest was crucial for selecting important  predictors from clinical and laboratory data to build a  survival prediction model for advanced gastric cancer  patients undergoing specific  chemotherapy. Its strength in managing complex  interactions and non-linear  relationships among  variables enabled the  accurate prediction of  survival outcomes in this study. | model development | internal validation | Random Forest Survival  Algorithm, Cox Regression Model | Serum Neutrophil-  Lymphocyte Ratio (NLR),  Alkaline Phosphatase Level, Albumin Level, Performance Status (eg; ECOG,  Karnofsky), Histologic  Differentiation (eg;  differentiation of tumors) | A total of 1733 patients  treated at the  Samsung Medical Center, Korea were included in the  study, and  clinicopathological and  laboratory data were  retrospectively analysed.  The dataset was split into a training set (n=1156, 67%) and a validation set (n=577, 33%). Top-ranked variables were identified using the  random forest survival  algorithm and integrated into a Cox  regression model, thereby constructing the scoring system  for predicting the overall survival of patients with advanced  gastric cancer. | The random forest survival  algorithm has outstanding  performance among survival prediction  models with high-  dimensional variables by  reducing the  dimensionality of datasets, and it can manage complex  interaction structures  consisting of highly  correlated  variables. The scoring  system  determined four distinct risk groups in the validation  dataset with median overall  survival of 17.1 months  (95% CI=14.9 to 20.5  months), 12.9 months  (95%CI=11.4 to 14.6  months), 8.1 months  (95%CI=5.3 to 12.3 months) and 3.9 months (95%CI=1.5 to 8.2 months), respectively. The area under the curve to  estimate the discrimination  performance of the scoring  system was 66.1 considering 1 year overall survival | We developed a simple and clinically  useful predictive scoring  model in a homogeneous  population with advanced gastric cancer treated with fluoropyrimidine-containing and platinum-containing  chemotherapy. However, additional independent validation  will be required before the scoring model can be used commonly. | Low |
| Klaassen, R et al | Feasibility of CT  radiomics to predict treatment response of individual liver  metastases in esophagogastric  cancer  patients(92) | 2018 | Netherlands | to investigate the utility of a CT radiomics approach in  predicting the response to  chemotherapy of individual liver metastases in patients with esophagogastric cancer (EGC). | 196 liver metastases | liver metastases in  esophagogastric cancer | Study Objective and Data Collection-Response  Classification-Role of  Random Forest (RF)-  Statistical Analysis and  Validation- Clinical  Implications and Conclusion.  Random Forest (RF) was crucial in this study for  effectively using a  comprehensive set of  radiomics features to predict chemotherapy response in  liver metastases. Its ability to handle complex data and  perform well in classification highlights its importance in advancing personalized  oncology. Further validation in larger, independent  cohorts is needed to confirm these findings for broader  clinical use. | model development | internal validation | Random Forest (RF) | Radiomics Features,  Treatment Response  Categories,  Partial Responding (PR),  Complete Remission (CR), Quantitative Assessment, Model Development,  Validation Strategy,  Performance Metrics | In this study, 18 patients with metastatic  esophagogastric cancer  underwent chemotherapy.  Liver metastases were  analyzed using CT scans,  extracting 370 radiomics  features per lesion. Random forest models were  developed to predict  treatment response—partial response (>65% volume  decrease) and complete  remission (100% volume  decrease)—using leave-one- out cross-validation. The  study found that CT  radiomics could potentially distinguish responding from  non-responding liver  metastases, highlighting the need for further validation in independent patient groups. | The RF model for PR lesions showed an average training AUC of 0.79 (range: 0.74–  0.83) and 0.65(95% ci: 0.57– 0.73) for the combined  validation set. The RF-model for CR lesions had an  average training AUC of 0.87 (range: 0.83–0.90) and 0.79 (95% ci 0.72–0.87) for the  valida tion set. | The study concludes that CT radiomics can potentially  predict chemotherapy  response in liver metastases of esophagogastric cancer patients. It highlights  variability in response,  demonstrates the model's  ability to distinguish between partial and complete  remission lesions based on  pre-treatment CT scans, and stresses the necessity for  additional validation in  independent patient cohorts to confirm these findings. | Low |
| Kuwayama, N et al | Applying artificial  intelligence using  routine clinical data for  preoperative  diagnosis and  prognosis evaluation of gastric cancer(93) | 2023 | Japan | To evaluate the prognosis of gastric cancer using artificial intelligence (AI) machine  learning technology based on routine blood collection data and to stratify patients into distinct prognostic  groups different from  conventional tumor-node-  metastasis (TNM)  classification. | 1687 patients | gastric cancer | Machine Learning  Approach-Feature  Importance-Performance Metrics-Comparison with Other Methods.  RF was pivotal for managing feature interactions and  identifying key predictors of survival and recurrence in  gastric cancer. Its ensemble approach and feature  importance revealed critical clinicopathological factors for long-term outcome  prediction, enhancing  prognostic stratification  beyond TNM classification in gastric cancer. | model development | internal validation | Logistic Regression (LR), Random Forest (RF),  Gradient Boosting (GB),  Deep Neural Network (DNN) | Demographic information (e.g., age, gender),  Tumor characteristics (e.g., tumor size, histological type, differentiation grade),  Treatment information (e.g., type of treatment received, response to treatment),  Clinical symptoms and signs (e.g., weight loss, pain,  dysphagia),  Pathological staging  information (e.g., TNM  classification),  5-Year Survival,  5-Year Recurrence-Free Survival | This study employed AI  machine learning to evaluate the prognostic value of  blood collection data in  gastric cancer. Clinical data from gastric cancer patients were analyzed using four  machine learning methods: Logistic Regression (LR),  Random Forest (RF),  Gradient Boosting (GB), and Deep Neural Network (DNN). These methods aimed to  classify patients into groups with either favorable or poor post-5-year prognosis based on clinicopathological data  and recurrence occurrence.  Feature selection was  performed to identify the  most relevant predictors,  which were then used for  patient clustering using the k-medoids method.  Predictive performance was assessed using accuracy  and Area Under the Curve  (AUC) for both 5-year  survival and recurrence-free survival. This approach  aimed to develop a novel prognostic stratification method that differs from traditional TNM  classification, potentially  improving prognostic  accuracy in gastric cancer. | The prediction accuracy and area under  the curve (AUC) for 5-year  survival were as follows: LR, 76.8% and 0.702; RF, 72.5% and 0.721; GB, 75.3% and  0.73;  DNN, 76.9% and 0.682,  respectively. The prediction  accuracy  and AUC for 5-year  recurrence-free survival were as follows:  LR, 85.5% and 0.692; RF, 79.0% and 0.721; GB,  80.5% and 0.718; DNN, 83.2% and 0.670. | The study concludes that AI machine learning, using  blood collection data,  effectively evaluates gastric  cancer prognosis. By  employing various machine learning models, including logistic regression, random forest, gradient boosting,  and deep neural networks, the study categorized  patients based on clinical  data and post-5-year relapse occurrence. This approach  offers a distinct stratification of prognosis compared to  traditional TNM  classification, showcasing AI's potential for  personalized prognostic assessment in gastric  cancer. | Low |
| Larue, RTHM et all | Pre-treatment CT  radiomics to predict 3-year  overall survival  following  chemoradiotherapy of  esophageal cancer(94) | 2018 | Netherlands | The aim of the study is to investigate the prognostic  value of pretreatment CT radiomic features in  predicting the overall  survival of esophageal  cancer patients following chemoradiotherapy. | 165 + 74 = 239 | esophageal cancer | Feature Selection- Model  Development-Prediction and Evaluation-Performance  Assessment-Clinical Utility Random Forest was crucial in this study for integrating and analyzing a large set of radiomic features derived  from pretreatment CT scans of esophageal cancer  patients. It served as a  powerful tool for building  predictive models of 3-year overall survival post-  chemoradiotherapy,  demonstrating its capability to leverage complex data  and improve prognostic assessments in clinical oncology. | both model development and model validation | Both internal and external validation | Random Forest (RF) Models, Clinical RF Model | Radiomic Features,  demographic data, tumor stage, treatment specifics  (chemotherapy and  radiotherapy regimens) | Two datasets of independent centers were analyzed,  consisting of esophageal  cancer patients treated with concurrent chemotherapy  (Carboplatin/Paclitaxel) and 41.4Gy radiother apy,  followed by surgery if  feasible. In total, 1049  radiomic features were  calculated from the primary tumor volume. Recursive  feature elimination was  performed to select the 40 most relevant predictors.  Using these 40 features and six clinical variables as input, two random forest (RF)  models predicting  3-year overall survival were developed | In total 165 patients from  center 1 and 74 patients  from center 2 were used.  The radiomics-based RF  model yielded an area under the curve (AUC) of 0.69  (95%CI 0.61–0.77), | A RF model predicting 3-  year overall survival based  on pretreatment CT radiomic features was developed and validated in two independent datasets of esophageal  cancer patients.  The radiomics model had better prognostic power compared to the model  using standard  clinical variables. | Moderate |

| Li, H et al | Integrative Analysis of  Histopathological Images and  Genomic Data in  Colon  Adenocarcinoma(95) | 2021 | China | The aim of the study is to  develop and validate an  integrative prognostic model for colon adenocarcinoma  (COAD) that combines  histopathological image  features with genomic data to improve the prediction of patient survival and assist in clinical decision-making. | 719 whole slide  histopathological images of  218 patients  were downloaded from  TCIA. | Colon adenocarcinoma | Model Construction-  Calculation of  Histopathological-Genomic  Prognosis Factor (HGPF)- Feature Importance-  Performance Evaluation. the Random Forest  algorithm is crucial in  developing a comprehensive and reliable prognostic  model by integrating multi- omics features, assessing the importance of these  features, and ultimately  improving the prediction of  survival outcomes in  patients with colon  adenocarcinoma. | model development | internal validation | Least Absolute Shrinkage and Selection Operator  (LASSO), Support Vector Machine (SVM), Weighted Gene Co-Expression  Network Analysis (WGCNA), Random Forest, Time-  Dependent Receiver  Operating Characteristic  (ROC) Curve, Kaplan-Meier Analysis | Histopathological Image  Features, Genomic Data,  HTSeq-counts mRNA  expression data from The Cancer Genome Atlas  (TCGA), Histopathological- Genomic Prognosis Factor (HGPF) | We downloaded 719 whole- slide histopathological  images from TCIA, and 459 corresponding HTSeq-  counts mRNA expression and clinical data were  obtained from  TCGA. Histopathological image features were  extracted by CellProfiler. Prognostic image  features were selected by  the least absolute shrinkage and selection operator  (LASSO)  and support vector machine (SVM) algorithms. The co-  expression gene module correlated with prognostic image features was  identified by weighted gene co expression network  analysis (WGCNA). Random forest was employed to  construct an  integrative prognostic model and calculate the  histopathological-genomic prognosis  factor (HGPF). | RF: In the training set, the 1-, 3-, and 5-year AUCs were 0.948, 0.916, and  0.933 respectively. In the test set, the 1-, 3-, and 5- year AUCs were  0.913, 0.894, and 0.924, respectively | These results suggested that the histopathological image features had a  certain ability to predict the survival of COAD patients. The integrative prognostic model  based on the  histopathological images  and genomic features could further improve the  prognosis prediction in  COAD, which may assist the clinical decision in the future. | High |
| --- | --- | --- | --- | --- | --- | --- | --- | --- | --- | --- | --- | --- | --- | --- | --- |
| Li, H et al | Establishment and  application of three predictive models of anastomotic leakage after rectal cancer  sphincter-preserving surgery(96) | 2023 | China | To develop nomogram,  decision tree, and random forest prediction models for AL  following sphincter-  preserving surgery for rectal cancer and to evaluate the pre dictive efficacy of the  three mode | 497 patients | rectal cancer | Prediction of Anastomotic Leakage (AL)- Comparison of Predictive Efficacy-  Evaluation of Predictive  Performance-Clinical Utility- Machine Learning Algorithm. the Random Forest model is used to accurately predict  the risk of AL following  sphincter-preserving surgery for rectal cancer,  outperforming other  predictive models and  offering significant clinical  benefits in terms of early  identification and prevention of this complication. | both model development and model validation | internal validation | Nomogram Model, Decision Tree Model, Random Forest Model | Age, Gender, Body Mass  Index (BMI), Smoking status, previous abdominal  surgeries, diabetes,  cardiovascular disease,  Tumor size and location, Stage of cancer (TNM  classification), Duration of surgery, Type of surgical  approach (e.g., laparoscopic vs. open surgery),  Intraoperative blood loss,  use of stomas,  Postoperative complications, Early postoperative nutrition | The clinical information of 497 patients with rectal  cancer who underwent  sphinc ter-preserving  surgery at Jincheng People’s Hospital of Shanxi Province between  January 2017 and  September 2022 was  analyzed in this study.  Patients were  divided into two groups: AL and no AL. Using univariate and multivariate analy ses, we identified factors  influencing postoperative  AL. These factors were used to  establish nomogram,  decision tree, and random forest models. The  sensitivity,  specificity, recall, accuracy, and area under the receiver operating characteristic  curve (AUC) were compared between the three models. | AL occurred in 10.26% of  the 497 patients with rectal cancer. The nomogram  model had an AUC of 0.922, sen sitivity of 0.745,  specificity of 0.966,  accuracy of 0.936, recall of  0.987, and accuracy of  0.946. The above indices in the decision tree model were 0.919, 0.833, 0.862, 0.951,  0.994, and 0.955,  respectively and in the  random forest model  were 1.000, 1.000, 1.000, 0.951, 0.994, and 0.955,  respectively. The DeLong test revealed that the AUC value of the decision-tree  model was lower than that of the random forest model (P < 0.05). | The random forest model may be used to identify  patients at high risk of AL after sphincter-preserving surgery for  rectal cancer owing to its  strong predictive effect and stability. | High |
| Li, J et al | A multicenter  random forest model for effective  prognosis prediction in  collaborative clinical research network(97) | 2020 | China | The aim of the study is to develop a multicenter  random forest prognosis  prediction model that can  utilize federated clinical data mining from horizontally  partitioned datasets to  improve the accuracy of  prognostic predictions. The study aims to address the  challenges associated with sharing sensitive biomedical data across multiple  institutions, ensuring privacy preservation while  maintaining or enhancing the  performance of the  prediction model compared to a centrally trained version. | ??? | colorectal cancer | Multicenter Prediction  Model- Overcoming  Performance Limitations- Feature Importance and Selection-Validation and  Evaluation- Privacy  Preserving Collaboration.  the study utilized the random  forest algorithm to create a multicenter model that  excels in privacy-preserving prognosis prediction,  facilitating effective  collaboration and advancing medical artificial intelligence. | model development | external validation | Random Forests (RF),  Differentially Private  Generative Adversarial  Network (GAN), Receiver  Operating Characteristic  (ROC) Curves, Filter-Based Linear Discriminant Analysis, Commonly Used Machine  Learning Methods, | Horizontally Partitioned  Datasets, Random Forests  (RF), GAN, Feature Selection without Sharing Patient-  Level Information, ROC  curves, Accuracy, Sensitivity, and Specificity, Colorectal  Cancer Datasets from the US and China,  Discrimination Ability,  Calibration Ability | In this study, a multicenter  random forest prognosis prediction model is  proposed that  enables federated clinical  data mining from horizontally partitioned datasets. By  using a novel data enhance ment approach based on a differentially private  generative adversarial  network customized to  clinical prognosis  data, the proposed model is able to provide a multicenter RF model with performances on par with—or even  better than—centrally  trained RF but without the need to aggregate the raw data. Moreover, our model also  incorporates an importance ranking step designed for  feature selection without  sharing patient-level informa tion. | for the group with low  heterogeneity, the  multicenter random forest  prediction model pro vided by the proposed model has better discrimination ability than thecentrally trained RF (multicenter RF vs. RF:  0.7676 to 0.7354,  p-value < 0.0001 from  t-test). Regarding the  calibration ability, the logistic regression model shows the best calibration ability in all the candidate  prediction models, and the multicenter random forest  model performs  slightly worse than the  logistic regression model (multicenter RF vs. LR:  4.0392 to 3.1393,  p-value = 0.0011 from  t-test). | The proposed random forest model exhibits ideal  prediction capability using  multicenter clinical  data and overcomes the performance limitation  arising from privacy  guarantees. It can also  provide feature  importance ranking across institutions without pooling the data at a central site.  This study offers a practical solution for building a  prognosis prediction model  in the collaborative clinical  research network and solves practical issues in real-world applications of medical  artificial intelligence. | Moderate |
| Li, Q et al | Screening lncRNAs with diagnostic and prognostic value for human stomach  adenocarcinoma  based on machine  learning and  mRNA-lncRNA co- expression network analysis(98) | 2020 | China | The aim of this study was to find the long noncoding  RNAs  (lncRNAs) acting as  diagnostic and prognostic biomarker of STAD. | 375 STAD tissues and 32  normal adjacent samples  from patients with STAD  were included in this study. | Stomach adenocarcinoma | Feature Selection- Model Construction. The study employs Random  Forest, a machine learning algorithm, to develop a  diagnostic model using  lncRNAs as biomarkers for  stomach adenocarcinoma  (STAD). Random Forest  ranks differentially expressed lncRNAs (DElncRNAs) by  their impact on accuracy to identify optimal diagnostic biomarkers. It constructs a predictive model alongside decision trees and SVM,  demonstrating strong  performance in diagnosing  STAD via metrics like AUC,  sensitivity, and specificity.  Thus, Random Forest is  pivotal in selecting predictive biomarkers and building a  robust diagnostic model for STAD in this study. | model development | internal validation | Decision Tree Model-  Random Forests Model- Support Vector Machine (SVM) Model | LINC01235, ESM1 | Base on TCGA dataset, the  differentially expressed  mRNAs (DEmRNAs) and  lncRNAs (DElncRNAs) were identified between STAD and normal tissue. The machine  learning and survival  analysis were performed to evaluate the potential  diagnostic and  prognostic value of lncRNAs  for STAD. We also build the co-expression network and functional annotation. The expression of selected  candidate mRNAs and  lncRNAs were  validated by Quantitative  real-time polymerase chain reaction (qRT-PCR) and  GSE27342  dataset. GSE27342 dataset were also to perform gene set enrichment analysis. | The area under curve (AUC) of the  decision tree model, random forests model, and support vector machine (SVM) model were 0.797, 0.981, and  0.983 | Our study identified three DElncRNAs as potential diagnostic biomark ers of STAD. Among them,  LINC01235 also was a  prognostic lncRNA  biomarkers | Moderate |
| Li, Z et al | Computed  tomography-based radiomics for  prediction of  neoadjuvant  chemotherapy  outcomes in locally advanced gastric cancer: A  pilot study(99) | 2018 | China | The aim of this study was to investigate the wealth of  radiomics  for pre-treatment computed tomography (CT) in the  prediction of the  pathological response of locally advanced  gastric cancer with  preoperative chemotherapy. | 30 patients | gastric cancer | Feature Selection-Model Training and Validation- Outcome Prediction-  Classifier Building In  this study, Random Forest is used for both feature  selection and classification. It selects key radiomic  features via Recursive  Feature Elimination (RFE-RF) and predicts neoadjuvant  chemotherapy outcomes. Comparing its performance with other classifiers,  Random Forest enhances  the model's robustness and  accuracy. | model development | internal validation | Filter based on linear  discriminant analysis (LDA), Random Forest, Support  Vector Machine (SVM), k-  Nearest Neighbors (k-  NN),Naïve Bayes, Logistic Regression, Decision Trees, Neural Networks, Gradient Boosting Machines | Tumor Regression Grade  (TRG), Radiomics Features from Pre-treatment CT  Scans, Feature Selection Methods (Filter-based  methods,  Wrapper methods,  Embedded methods,  Dimensionality reduction methods), Classification Models (Random Forest, Support Vector Machine (SVM),  k-Nearest Neighbors (k-NN), Naive Bayes,  Logistic Regression,  Decision Trees,  Neural Networks,  Gradient Boosting Machines) | Thirty consecutive patients with CT-staged II/III gastric  cancer receiving  neoadjuvant chemotherapy were enrolled in this study  between December 2014  and March 2017. All patients underwent upper abdominal CT  during the unenhanced, late arterial phase (AP) and portal venous phase (PP) before  the administration of  neoadjuvant chemotherapy. In total, 19,985 radiomics  features were extracted in the AP and PP for each  patient.  Four methods were adopted during feature selection and eight methods were used in the process of building the classifier model. Thirty-two combinations of feature  selection and classification methods were examined.  Receiver  operating characteristic  (ROC) curves were used to evaluate the capability of  each combination of feature selection  and classification method to predict a non-good response (non-GR) based on tumor  regression grade (TRG). | RF, which achieved the  highest  prognostic performance in PP (AUC, 0.722±0.108;  accuracy, 0.793; sensitivity, 0.636; specificity, 0.889;  Z=2.039; P=0.041) | It is possible to predict non- GR after neoadjuvant  chemotherapy in locally  advanced gastric  cancers based on the  radiomics of CT. | High |
| Chao Li et al. | Radiomics Signature Based on Support  Vector Machines for the Prediction of  Pathological  Complete Response to Neoadjuvant  Chemoradiotherapy in Locally Advanced Rectal Cancer(100) | 2023 | China | The aim of this study is to develop and validate a  radiomics model based on  pretreatment ^18F-  fluorodeoxyglucose (FDG)  positron emission  tomography/computed  tomography (PET/CT)  images to predict  lymphovascular invasion (LVI) in patients with rectal cancer. | The study involved a total of  211 patients with locally  advanced rectal cancer  (LARC). These patients were  divided into two cohorts: a  training cohort with 148  patients and a validation  cohort with 63 patients. The  analysis focused on  evaluating the predictive  performance of various  radiomics signatures derived  from different machine  learning algorithms on these  cohorts. | Locally advanced rectal cancer | The random forest algorithm was used to select important  radiomic features from  pretreatment ^18F-FDG  PET/CT images. These  selected features were then used to build the predictive model for lymphovascular  invasion(LVI) in rectal cancer patients. | Model Development:  Radiomic feature extraction, feature selection using  random forest, and logistic  regression for model  construction.  Model Validation: Internal  validation using training and validation sets, with AUC as the primary performance  metric. | Internal validation | Radiomics-based predictive model combining random  forest (for feature selection) and logistic regression (for prediction). | Radiomic features extracted  from ^18F-FDG PET/CT  images, including texture, shape, and intensity  descriptors. | Patient Selection: 268 rectal cancer patients.  Image Acquisition: ^18F- FDG PET/CT scans.  Feature Extraction: Radiomic features from PET images.  Feature Selection: Random forest algorithm.  Model Development:  Logistic regression.  Model Validation: Internal validation with AUC as the performance metric.. | Performance of the SVM-  based Radscore: The SVM- derived radiomics score  (Radscore) showed superior predictive capabilities  compared to other machine learning methods, such as  least absolute shrinkage and selection operator (LASSO) and random forest (RF). The SVM Radscore achieved  area under the receiver  operating characteristic  curves (AUCs) of 0.880 in  the training cohort and 0.830 in the validation cohort.  Development of a Predictive Nomogram: By integrating  the SVM-based Radscore  with clinical indicators, the  researchers developed a  nomogram for predicting  pCR. This nomogram further improved predictive  performance, with AUCs of 0.910 in the training cohort and 0.866 in the validation  cohort. The nomogram was validated through calibration curves and decision curve  analyses, confirming its effectiveness and clinical applicability. | A Radscore derived from  SVM and based on  pretreatment contrast-  enhanced planning  CT scans was developed for predicting pCR in patients  with LARC. The Radscore exhibited  promising performance in accurately predicting pCR. Moreover, the proposed  nomogram,  which incorporates the  SVM-derived Radscore and clinical indicators, holds  considerable  value as a non-invasive tool for evaluating treatment  outcomes in LARC patients. This approach  has the potential to enhance clinical decision making and  contribute to personalized patient management  strategies. | High |
| Duco T. Mülder et al. | Development and validation of  colorectal cancer  risk prediction tools: A comparison of  models(101) | 2023 | Netherlands | The aim of the study was to develop and validate  colorectal cancer (CRC) risk  prediction tools by  comparing the effectiveness of a logistic regression (LR) model and a random forest (RF) model. The goal was to improve cancer screening  programs by allowing for risk-adjusted screening intensities, thereby  identifying individuals at elevated risk more  accurately. The study sought  to validate an existing LR  model and explore the  potential improvements  offered by a more flexible  machine learning approach, specifically the RF model. | The study involved 219,257  third-round participants from  the Dutch CRC screening  program up until 2018.  Additionally, two out-of-  sample validation cohorts  were used: 1,137,599 third-  round participants  post-2018 and 192,793  fourth-round participants  from 2020 onwards. | Colorectal cancer | The random forest (RF)  model was developed to  predict colorectal cancer  (CRC) risk using updated data from the Dutch CRC screening program. It was trained on 219,257 third-  round participants and  validated on two out-of-  sample cohorts: 1,137,599 third-round participants  post-2018 and 192,793 fourth-round participants from 2020 onwards. | The random forest (RF)  model was developed using data from 219,257 third-  round participants of the Dutch CRC screening  program up to 2018. The  model was then validated on two external cohorts:  1,137,599 third-round  participants post-2018 and 192,793 fourth-round  participants from 2020  onwards | External validation | The logistic regression (LR) model was used for  validation in the study. This model was validated using two external cohorts:  1,137,599 third-round  participants post-2018 and 192,793 fourth-round  participants from 2020  onwards. | The factors used for  prognostication in the study included sex, age, and two  preceding faecal  haemoglobin  concentrations. These  variables were used to  predict the risk of colorectal cancer (CRC) and advanced  neoplasia (AN) in the  subsequent screening  rounds. | Data Collection: The study utilized data from the Dutch CRC screening program,  including 219,257 third- round participants up to 2018.  Model Development: An  existing logistic regression  (LR) model was validated  and a new random forest  (RF) model was developed. Training: Both the LR and RF models were trained on the  third-round participant data. Validation: The models were externally validated using  two cohorts:  1,137,599 third-round  participants post-2018  192,793 fourth-round  participants from 2020  onwards.  Evaluation: The performance of the models was assessed using the area under the  receiver operating  characteristic curve (AUC)  and relative risks for  predicting advanced  neoplasia (AN) and  colorectal cancer (CRC). | The study found that both the logistic regression (LR) and random forest (RF)  models had similar  performance in predicting  advanced neoplasia (AN)  and colorectal cancer (CRC).  The LR model had an AUC of 0.77 for third-round  participants and 0.73 for  fourth-round participants.  The RF model also had an AUC of 0.77 for third-round participants and 0.73 for  fourth-round participants.  The highest risk 5% had a seven-fold increased risk of  AN compared to the  average, while the lowest  risk 80% had below-average risk. The LR model remained  preferred due to its  interpretability. | The LR model is a valid  method for predicting CRC risk in stool-based screening programs. Although its  predictive performance  slightly declined in later  rounds, it remained effective.  The RF model did not  improve CRC risk prediction compared to LR, likely due to the limited number of  explanatory variables. Thus, the LR model remains the  preferred prediction tool due to its interpretability. | Low |
| Myung-Giun Noh et al. | Practical prediction model of the clinical  response to  programmed death- ligand 1 inhibitors in advanced gastric cance(102) | 2021 | South Korea | To develop a reliable and  practical predictive model  for determining the clinical  response of patients with  advanced gastric cancer to PD-L1 inhibitors. This model was intended to enhance  personalized treatment  strategies, allowing clinicians to identify patients who are  more likely to benefit from PD-L1 inhibitor therapies and thereby improve  treatment outcomes | The study involved 533  patients with advanced  gastric cancer who were  treated with programmed  death-ligand 1 (PD-L1)  inhibitor | Advanced Gastric cancer | Random forest was applied by selecting input features  and splitting the dataset into training and testing sets. The algorithm built multiple  decision trees during training and averaged the  predictions for better  accuracy and to mitigate overfitting. Specific  parameters and features used in the model were  tuned to optimize  performance. | The model underwent  (internal/external) validation. Internal validation involved  dividing the original dataset into training and testing sets, ensuring the model was  evaluated on unseen data from the same distribution. External validation was  performed using a  completely independent dataset, providing a more rigorous assessment of  model generalizability. | Internal validation | Splitting the original dataset into training and testing  subsets to ensure the  model's performance was evaluated on unseen data  from the same distribution. This method helped assess the model's accuracy and reliability in predicting  clinical responses within the same dataset context. | Clinical Features: Patient  age, gender, performance status, and prior treatment history.  Molecular Features: PD-L1 expression levels, tumor  mutational burden, and  specific genetic alterations. | Data Collection: Clinical and molecular data from patients with advanced gastric  cancer treated with PD-L1 inhibitors were collected.  Feature Selection: Relevant features such as patient  demographics, clinical  characteristics, and  molecular markers were identiﬁed.  Model Development: A  random forest algorithm was used to develop the  predictive model. The  dataset was split into  training and testing subsets for model training and  validation.  Validation: Internal validation was performed by evaluating the model's performance on the testing subset to ensure its predictive accuracy and  reliability. | The study successfully  developed a predictive  model using the random  forest algorithm to estimate the clinical response to PD- L1 inhibitors in advanced  gastric cancer. The model demonstrated good  predictive accuracy during  internal validation, indicating its potential utility in clinical settings. Key prognostic  factors identified included PD-L1 expression levels, tumor mutational burden, and specific genetic  alterations. The results  suggest that the model  could help personalize  treatment plans and improve patient outcomes | The predictive model has  potential clinical utility in  personalizing treatment  strategies for advanced  gastric cancer patients,  optimizing the use of PD-L1 inhibitors and potentially  improving patient outcomes. | Low |

| Raoof Nopour | Prediction of five-  year survival among esophageal cancer patients using  machine learning(103) | 2023 | Iran | The aim of the study was to develop a prediction model for the five-year survival of esophageal cancer (EC)  patients in northern Iran  using machine learning (ML) algorithms. This model is  intended to enhance clinical outcomes and inform  various treatment and  preventive plans | The study used a single-  center database containing  data on 1656 EC patients. Of  these, 1255 were non-  survived cases, and 401  were survived cases | Esophageal cancer | The study used the Random Forest (RF) algorithm as one of the machine learning  techniques to develop  prediction models for the five-year survival of  esophageal cancer (EC)  patients. The performance of the RF model was assessed  using various evaluation  criteria, including negative  predictive value (NPV),  positive predictive value  (PPV), sensitivity, specificity, accuracy, Kappa, and F-  Score. The area under the  receiver operator  characteristics (AU-ROC)  was used for comparing the performance effectiveness of the different models. | Data Collection:  The study retrospectively  collected data from 1,656  esophageal cancer patients treated at Imam Khomeini  Hospital in Sari City between 2013 and 2020.  Feature Selection:  Multivariable regression  analysis was employed to identify the best predictors for five-year survival.  Significant predictors  included age at diagnosis,  BMI, smoking status, tumor characteristics, and  treatment variables.  Model Selection:  Several machine learning algorithms were evaluated, including:  Random Forest  eXtreme Gradient Boosting (XGBoost)  Support Vector Machine (SVM)  Artificial Neural Networks  (ANN)  Bayesian Networks  J-48 Decision Tree  K-Nearest Neighbors (KNN) | External validation | Random Forest (RF)  eXtreme Gradient Boosting (XGBoost)  K-Nearest Neighbors (KNN)  J-48 Decision Tree  Bayesian Network (BN) Artificial Neural Network  (ANN)  Support Vector Machine (SVM) | Clinicopathological  Variables,Laboratory  Indicators,Demographic and  Lifestyle Factors,Clinical Symptoms,Treatment-  Related Factors | Data Gathering and  Familiarization:  Collected data from EC  patients referred to Imam  Khomeini Hospital in Sari  City from 2013 to 2020.  Preparing and Analyzing the Dataset:  Removed redundant cases. Addressed missing values  by omitting cases with more than 10% missing data and ﬁlling the rest with the mode of each feature.  Conducted feature selection  to improve model  performance.  Model Development and Assessment:  Developed prediction  models using seven machine learning algorithms: Random  Forest (RF), eXtreme  Gradient Boosting  (XGBoost), K-Nearest  Neighbors (KNN), J-48 Decision Tree, Bayesian Network (BN), Artiﬁcial  Neural Network (ANN), and Support Vector Machine  (SVM).  Employed grid search for hyperparameter tuning.  Evaluated model  performance using various metrics, including NPV, PPV,  sensitivity, speciﬁcity,  accuracy, Kappa, F-Score, and AUC-ROC.  K-Fold Cross-Validation: Utilized stratiﬁed 10-fold cross-validation for  performance measurement to ensure generalizability  and eﬃciency.  External Validation Cohort: Tested the model's  generalizability using  external data from Imam  Khomeini Hospital in Tehran, involving 54 non-survived  and 46 survived samples | The outcome of the study indicated that the Random Forest (RF) model was the best-performing model for predicting five-year survival in esophageal cancer  patients, achieving an Area  Under the Receiver  Operating Characteristic  Curve (AUC-ROC) of 0.95.  The RF model demonstrated high classification capability with an accuracy of 96.9%, sensitivity of 98.8%,  specificity of 91%, and a Kappa statistic of 91.5% | The conclusion of the study is that the Random Forest  (RF) model was the most effective in predicting five- year survival among  esophageal cancer patients. The RF model demonstrated  superior performance  metrics, including an Area Under the Curve (AUC) of  0.95, accuracy of 96.9%,  and high sensitivity and  specificity. This study  emphasizes the importance of using machine learning  algorithms, particularly  ensemble methods, for  improving survival  predictions in clinical  settings | Low |
| --- | --- | --- | --- | --- | --- | --- | --- | --- | --- | --- | --- | --- | --- | --- | --- |
| Raoof Nopour | Design of risk  prediction model for esophageal cancer based on machine  learning approach(104) | 2024 | Iran | The aim of the study titled "Design of risk prediction model for esophageal  cancer based on machine learning approach" was to develop a risk prediction  model for esophageal  cancer (EC) using machine learning (ML) techniques.  The objective was to  leverage ML to stratify high- risk individuals for EC and to achieve efficient preventive measures at the community level by modifying  associated risk factors and implementing other clinical solutions | The population of the study  consisted of 3,256 cases of  esophageal cancer (EC) and  non-EC, collected  retrospectively from 2018 to  2022 in Sari City | Esophageal cancer | The study used a Random  Forest (RF) algorithm to  develop the esophageal  cancer risk prediction model by training it on 70% of the data, validating with 10%,  and testing with 20%. The hyperparameters for the RF model, such as max depth, number of iterations, and  number of randomly  selected features, were  optimized using grid search | Splitting the data into 70%  for training, 10% for  validation, and 20% for  testing.  Optimizing hyperparameters, such as max depth, number of iterations, and the number of randomly selected  features, using a grid search method to achieve the best performance | External validation | The Random Forest (RF)  algorithm was used as one of the six machine learning models to develop the  esophageal cancer (EC) risk prediction model.  The data was split into 70%  for training, 10% for  validation, and 20% for  testing.  Hyperparameters such as max depth, number of  iterations, and the number of randomly selected features were optimized using grid  search | Age  Sex  Body Mass Index (BMI)  Smoking  Gastroesophageal Reflux Disease (GERD)  Barrett’s esophagus  Weight loss  Fruit consumption  Vegetable consumption Drinking hot liquids  High fat intake  Physical inactivity  Achalasia  History of certain other  cancers  HPV infection  Lye ingestion  Red meat consumption  History of radiotherapy  Spicy and salty food  consumption  Nervousness and anxiety  Income level  Educational level  Race  Place of residence  Occupation type | Study Design and Setting:  The study was a longitudinal and retrospective analysis  performed from 2018 to 2022 in three clinical  settings: Tooba Clinic,  Hekmat, and Imam Khomeini  Hospitals in Sari City,  Mazandaran province.  An integrated electronic  database containing data from 3256 samples of  individuals suspected of  having esophageal cancer (EC) was used. Among  these, 1283 cases had  positive diagnostic results, and 1973 had negative  results.  Study Roadmap:  The study was conducted in seven phases:  Data Acquisition: Collecting the data from the clinical  settings.  Database Description and Familiarization:  Understanding and  describing the structure and content of the database.  Data Preprocessing:  Cleaning and preparing the data for analysis.  Feature Selection: Identifying the most relevant features  for predicting EC risk.  Machine Learning Model  Selection and Development: Selecting and developing  appropriate ML models.  Performance Evaluation:  Evaluating the performance of the developed models.  External Validation:  Validating the model with an external dataset.  Machine Learning  Algorithms:  Six ML algorithms were used  to develop the risk  prediction model for EC: Random Forest (RF)  eXtreme Gradient Boosting (XG-Boost)  Bagging  K-Nearest Neighbor (K-NN) Support Vector Machine  (SVM)  Artiﬁcial Neural Networks  (ANNs)  Performance Comparison: The performance of these algorithms was compared, with the XG-Boost model  achieving the best  predictability with AU-ROC scores of 0.92 for internal  validation and 0.889 for  external validation.  Predictors:  The top predictors of EC risk identiﬁed by the XG-Boost  model were sex, drinking hot liquids, fruit consumption,  achalasia, and vegetable consumption | The outcome of the study  showed that the eXtreme  Gradient Boosting (XG-  Boost) model had the best predictability for esophageal cancer risk, with AU-ROC  scores of 0.92 for internal validation and 0.889 for  external validation | The conclusion of the study is that the eXtreme Gradient Boosting (XG-Boost) model, an ensemble machine  learning approach,  demonstrated high  predictability and  generalizability in predicting esophageal cancer (EC) risk. It showed potential benefits for screening individuals at high risk for EC, suggesting that such predictive models can be used effectively in  preventive medicine to  improve overall health  outcomes and reduce the  need for costly interventional treatments. The model's  implementation in clinical decision support systems could aid healthcare  providers in better risk  stratification and preventive measures | Low |
| Beth L Nordstrom et al. | Validation of Claims Algorithms for  Progression to  Metastatic Cancer in Patients with Breast, Non-small Cell Lung, and Colorectal  Cancer(105) | 2016 | United states | The aim of the study was to develop and validate  algorithms for identifying the progression to metastatic  cancer in patients with  breast cancer, non-small cell lung cancer (NSCLC), and  colorectal cancer (CRC)  using routinely collected  claims data. The researchers sought to create algorithms that could accurately  determine cancer  progression, facilitating more effective research on the  effectiveness and safety of oncology treatments based  on these data | The study included 1,017  patients diagnosed with  breast cancer, non-small cell  lung cancer (NSCLC), or  colorectal cancer (CRC) from  2004 to 2011 within the  Geisinger Health System.  Specifically, there were 502  breast cancer patients, 236  NSCLC patients, and 279  CRC patients. The median  ages were 62, 72, and 71  years for breast cancer,  NSCLC, and CRC patients,  respectively. Most patients  were non-Hispanic whites,  with men making up the  majority in the NSCLC  (56.8%) and CRC (52%)  groups | Colorectal cancer | In the study, random forests (RF) were used to evaluate the importance of various  variables in predicting the progression to metastatic cancer. RF is a machine  learning technique that  builds multiple decision trees and aggregates their results to enhance prediction  accuracy. The study used RF to rank the importance of  potential predictor variables, which helped in narrowing  down to a more concise set of variables for the final  predictive models. The RF process involved running  100 forests with 1000 trees each and averaging the  results to determine the  most significant predictors | The study used the Random Forest algorithm to develop a prognostic model for  predicting survival in lung adenocarcinoma patients.  They utilized gene  expression data and clinical variables as input features for the model. The model's performance was assessed using internal validation  techniques, specifically a cross-validation approach,  to ensure the robustness and reliability of the  predictions. | Internal validation | random forests models were employed to construct and  evaluate several algorithms for identifying progression to metastatic cancer in patients with breast, non-small cell  lung, and colorectal cancer. These models were utilized to assess the performance measures of the algorithms, such as sensitivity,  specificity, positive  predictive value (PPV), and negative predictive value  (NPV). | Secondary Malignancy  Diagnosis: The presence of ICD-9 codes for secondary malignancy.  Radiology or Pathology  Claims: Radiology or  pathology claims appearing ≥60 days after the initial  cancer diagnosis.  Diagnosis of Secondary  Malignancy: Diagnosis of  secondary malignancy or a second primary tumor.  Initiation of Immunotherapy: Particularly for breast cancer  patients, the initiation of immunotherapy was a  significant factor.  Change in Chemotherapy Regimen: Relevant for non- small cell lung cancer  (NSCLC) and colorectal  cancer (CRC) patients.  Use of Pain Medication: Specifically noted for  NSCLC patients.  Initiation of Metastatic-  Specific Therapy: Also  significant for NSCLC  patients | Data Collection: Gene  expression data and clinical variables were collected  from lung adenocarcinoma patients.  Feature Selection: Important features were identiﬁed  using the Random Forest algorithm.  Model Building: The  Random Forest model was trained using the selected features.  Validation: The model was  validated using a 10-fold  cross-validation approach to assess its predictive  performance and  robustness. | The outcome of the study demonstrated that the  Random Forest-based  prognostic model effectively predicted the survival of lung adenocarcinoma patients.  The model showed a high level of accuracy and  robustness through internal validation using a 10-fold  cross-validation method.  The results indicated that the model could reliably stratify patients into different risk  categories based on their  gene expression profiles and clinical variables, potentially aiding in personalized  treatment strategies for lung adenocarcinoma. | The study concluded that  while simple algorithms  using secondary malignancy diagnosis codes had some  utility, they generally had low sensitivity and positive  predictive value (PPV) but higher specificity and  negative predictive value (NPV). Accurate  identification of cancer  progression likely requires verification through manual chart review to improve  these measures | High |
| Jing Ou et al. | CT radiomic features for predicting  resectability of  oesophageal  squamous cell  carcinoma as given by feature analysis: A case control study(106) | 2019 | China | The aim of the study was to evaluate the ability of CT  radiomic features to predict the resectability of  esophageal squamous cell carcinoma (ESCC). The  researchers sought to  develop and validate a  radiomic model that could help in distinguishing  between resectable and  unresectable cases of ESCC using extracted CT imaging features | The study included a total of  591 patients with  esophageal squamous cell  carcinoma (ESCC). This  population was divided into  a training cohort of 413  patients (189 resectable and  224 unresectable) and a  validation cohort of 178  patients (81 resectable and  97 unresectable) | Oesophageal squamous cell carcinoma | Random forest was used as one of the machine learning methods to create a  predictive radiomic model. It served as an ensemble  learning method based on  Bagging, which is suitable  for classification, regression, and other problems. The  random forest model  classified instances based  on the optimal radiomic  features selected during the feature extraction and  selection process | The model development  included using multivariable logistic regression, decision tree, random forest, support vector machine (SVM), and X-Gradient boost methods.  Validation was performed internally, with patients  randomly allocated to  training (413 patients) and validation (178 patients)  cohorts | Internal validation | The model used for  validation included multiple machine learning methods:  multivariable logistic  regression, decision tree,  random forest, support  vector machine (SVM), and X-Gradient boost. These  models were developed  using the selected radiomic features and validated  internally with a separate  validation cohort of patients | The factors used for  prognostication included  sex, drinking hot liquids, fruit consumption, achalasia, and vegetable consumption.  These factors were ranked based on their relative  importance using the XG- Boost model | The method of the study  was a longitudinal and  retrospective analysis that  involved extracting radiomic  features from thoracic  contrast-enhanced CT scans of patients with esophageal squamous cell carcinoma.  Machine learning models,  including logistic regression, decision tree, random forest, support vector machine, and X-Gradient boost, were  developed and validated internally using these  features to predict tumor resectability | The model development and validation in the study  involved using the XG-Boost algorithm, which showed the best performance for  predicting esophageal  cancer risk. The model was  validated both internally and externally. External validation was performed using 75  esophageal cancer cases and 101 non-cancer cases from Valieasr AJ Hospital, achieving an accuracy of 85% | The conclusion of the study was that the XG-Boost  algorithm showed potential benefits in screening  individuals for esophageal cancer by effectively  stratifying risk and  identifying high-risk groups.  This model demonstrated high predictability and  generalizability in both  internal and external  validations, suggesting its applicability in various  clinical settings for early prediction and preventive measures against  esophageal cancer | Moderate |
| Misun Park et al. | A patient-derived  organoid-based  radiosensitivity  model for the  prediction of  radiation responses in patients with  rectal cancer(107) | 2021 | South Korea | The aim of the study was to develop a patient-derived  organoid-based model for predicting the  radiosensitivity of rectal  cancer patients to radiation therapy. This model aims to help in tailoring  individualized radiation  treatments based on the  predicted responses of the patient's cancer cells | The study included 113,663  participants in the  population sample. | Rectal cancer | The random forest algorithm was utilized to develop a  predictive model by  analyzing patient-derived  organoid data to determine radiosensitivity. | Model development in this  study involved creating a  predictive model based on  the collected data to identify patterns and trends related to the research question.  This process likely included defining the variables to be included in the model,  selecting an appropriate  modeling technique (e.g.  regression analysis, machine learning algorithm), and  evaluating the model's  performance through  validation and testing. The researchers may have also  conducted sensitivity  analyses and model  refinement to improve the accuracy and reliability of the predictive model.  Overall, model development in this study was a  systematic and iterative  process aimed at deriving  meaningful insights from the data. | External validation | Specifically, it employed patient-derived organoids (PDOs) to predict the  radiosensitivity of rectal  cancer patients. Additionally, they used clonogenic assays and in vivo xenograft models to validate the  radiosensitivity predictions. | Tumor Regression Grade  (TRG): This is a measure of how much the tumor has  shrunk or changed in  response to treatment.  TNM Downstaging:  Comparison of pre-  treatment clinical staging and post-treatment  pathological staging to assess tumor response. Survival Fraction Data:  Quantitative data on the  proportion of cells that  survive radiation treatment.  Machine Learning  Predictions: Utilizing  algorithms like random  forest to predict good and poor responders based on clinical and experimental radioresponse data | Patient Enrollment:  Prospectively enrolling 33 patients with mid-to-lower rectal adenocarcinoma.  Organoid Culture: Deriving tumor organoids from  patient biopsies.  Radiation Treatment:  Subjecting both patient- derived organoids and patients to irradiation.  Response Assessment:  Measuring radiation  responses in organoids and comparing them with clinical outcomes.  Machine Learning: Using  survival fraction data to train machine learning models  (e.g., random forest) to  predict patient  radiosensitivity and  treatment outcomes. | The outcome of the study  showed that the radiation  responses of patient-derived organoids closely matched the clinical outcomes of the corresponding patients. The machine learning models  used for prediction  demonstrated high accuracy, with the model for good  responders achieving an  accuracy of 81.5% and the model for poor responders achieving an accuracy of  92.1%. This indicated that  the organoid-based model could effectively predict  individual patient responses to radiotherapy, supporting its potential for personalized treatment strategies | The conclusion of the study was that patient-derived  organoids effectively mimic the radiation responses of rectal cancer patients, and  the organoid-based  radiosensitivity model can  accurately predict patient  outcomes. This model has  significant potential to  enhance precision medicine in treating rectal cancer by allowing personalized  prediction of radiotherapy responses | Low |
| Desbordes Paul et al. | Feature selection for outcome prediction in oesophageal  cancer using genetic algorithm and random forest  classifier(108) | 2016 | France | The aim of the study was to develop a feature selection strategy using a genetic  algorithm and random forest classifier to identify the most relevant predictive and  prognostic features for  outcome prediction in  oesophageal cancer | The population of the study  consisted of 65 patients with  locally advanced  oesophageal cancer who  were eligible for  chemoradiation therapy | Oesophageal cancer | The random forest was used to develop the model by  constructing a multitude of independent trees from a bootstrap sample of  patients. Each tree was built using a subset of features  randomly selected at each node, and the final  classification was made through a majority vote among the trees. This  method was evaluated using a K-fold cross-validation  technique, specifically with K = 5 | The model development  involved constructing a  multitude of independent  trees from a bootstrap  sample of patients, with  each tree built using a  subset of features randomly selected at each node. The final classification was  determined by majority  voting among the trees. The  validation was internal, using a 5-fold cross-validation  method to evaluate the  accuracy of the model | Internal validation | The model used for  validation was a random  forest classifier, evaluated by a 5-fold cross-validation  method | The factors used for  prognostication were patient age, disease location, stage, WHO stage, NRI (group 2),  IDM from the GLC matrix (group 7), SZE from the GLSZ matrix, and HGZE from the GLSZ matrix | The method of this study  involved a feature selection strategy using a Genetic  Algorithm combined with a Random Forest (GARF). It  consisted of two steps: first,  eliminating correlated  features using Spearman's rank correlation analysis,  and second, selecting the most relevant subsets of  features using a genetic  algorithm with a multi-  parametric fitness function based on a random forest classifier, AUC  measurement, and a sparsity constraint | The study achieved an  outcome with a random  forest misclassification rate of 18 ± 4% and an AUC of  0.823 ± 0.032 for predictive results, and an error rate of 20 ± 7% and an AUC of  0.750 ± 0.108 for prognostic results. | The study concluded that  the GARF method improves  outcome prediction  compared to other tested  methods by at least 8% for predictive studies and 11% for prognostic ones. It  highlighted the usefulness of machine learning  techniques, particularly  random forests, in selecting subsets of multimodal  features | Moderate |
| Manuel Pera et al. | Machine Learning Risk Prediction Model of 90-day  Mortality After Gastrectomy for  Cancer(109) | 2022 | Spain | The aim of the study was to develop a clinically useful  risk prediction model for 90- day mortality after gastric  cancer resection with  curative intent, followed by  internal-external validation of the model | The study population  comprised 3,182 patients  from the SEEGCR database,  who had primary gastric or  gastroesophageal junction  (GEJ) cancer and underwent  partial or total gastrectomy  with curative intent from  January 2014 to October  2021 | Gastric cancer | Random Forest (RF) was  used to predict 90-day  mortality by combining  decision trees built on  subsampling of the dataset. It was applied alongside  other models, such as cross- validated elastic net  regularized logistic  regression (cv-Enet) and  glmboost. To address class imbalance, the Synthetic  Minority Over-sampling  Technique (SMOTE) was  used during model training.  Hyperparameter tuning was performed using nested  resampling, specifically a k- fold cross-validation  procedure nested inside another k-fold cross-  validation for model  selection. For prediction, a 10-fold cross-validation with  5 repeats and 1000  evaluations in random  search was used | The model development and  validation used 10-fold  cross-validation to evaluate performance, incorporating methods such as Random Forest, cv-Enet, and  glmboost. Calibration was  checked visually and with  the Hosmer-Lemeshow test, ensuring robust internal  validation. | Internal validation | The model used for  validation was a 10-fold  cross-validation procedure | Age  Hospital volume activity  Preoperative albumin level Preoperative hemoglobin level  Type of gastrectomy (total/ subtotal)  History of chronic  obstructive pulmonary  disease (COPD) | Consecutive patients with  gastric adenocarcinoma who  underwent potentially  curative gastrectomy  between 2014 and 2021 registered in the Spanish EURECCA  Esophagogastric Cancer Registry database were  included. The 90DM for all  causes was  the study outcome.  Preoperative clinical  characteristics were tested in four 90DM  predictive models: Cross  Validated Elastic regularized logistic regression method (cv-  Enet), boosting linear  regression (glmboost),  random forest (RF), and an ensemble model.  Performance was evaluated using the area under the  curve (AUC) by 10-fold  crossvalidation. | 3,182 and 260 patients from  39 institutions in six regions were included in the  development and validation cohorts, respectively. The  90DM rate was 5.6% and 6.2%,  respectively. The RF model showed the best  discrimination capacity with a validated AUC  of 0.844 (95% confidence interval [CI] 0.841-0.848) as compared with cv-Enet  (0.796,  95% CI 0.784-0.808)  glmboost (0.797, 95% CI  0.785-0.809), and ensemble  model (0.847,  95% CI 0.836-0.858) in the development cohort. Similar discriminative capacity was observed in the validation  cohort. | A robust clinical model for predicting the risk of 90DM after surgery of  gastric cancer was  developed. Its use may aid patients and surgeons in  making informed  decisions. | Low |

| Filippo Pietrantonio et al. | Estimating Survival Probabilities of  Advanced Gastric  Cancer Patients in  the Second-Line  Setting: The Gastric Life Nomogram(110) | 2018 | Italy | The aim of the study was to develop a prediction model for 90-day mortality in  patients undergoing gastric cancer resection using a  large population-based,  multicenter, and audited  clinical database combined with machine learning  techniques | The study population  consisted of 320 evaluable  patients in the development  set treated at 26 institutions  between 2006 and 2016,  and 295 and 172 evaluable  patients in the Italian and  Korean validation sets,  respectively | Advanced Gastric cancer | Random forest was used in this study by fitting the  model using the rfsrc  function in the  randomForestSRC package | Model development and  validation involved using  synthetic minority technique (SMOTE) for training,  hyperparameter tuning with nested resampling, and 10- fold cross-validation. Model performance was evaluated using the area under the  ROC curve (AUC), and  internal validation was  performed with 10-fold  cross-validation | External validation | The model used for  prognostication in the study was a combination of cross-  validated elastic net  regularized logistic  regression (cv-Enet), random forest (RF), and glmboost  models | The factors used for  prognostication were age, hospital volume activity,  preoperative albumin and hemoglobin levels, type of gastrectomy, and history of chronic obstructive  pulmonary disease | The nomogram was  developed on a set of 320  Italian patients and validated on two independent sets  (295 Italian and 172 Korean  patients). Putative  prognostic variables were  selected using a random  forest model and included in the multivariable Cox model.  The nomogram's  performance was evaluated by calibration plot and C  index. | ECOG performance status, neutrophils to lymphocytes ratio, and peritoneal  involvement were selected and included into the  multivariable model. The C index was 0.72 (95% CI  0.68-0.75) in the  development set, 0.69 (95% CI 0.65-0.73) in the Italian  validation set, but only 0.57 (95% CI 0.52-0.62) in the  Korean set. While Italian  calibrations were quite good, the Korean one was poor.  Regarding 6-month OS  predictions, calibration was best in both Caucasian  cohorts and worst the in Asian one. | Our nomogram may be a  useful tool to predict 3- or 6- month OS in Caucasian  gastric cancer patients eligible for second-line therapy. Based on three  easy-to-collect variables, the Gastric Life nomogram may help clinicians improve  patient selection for second-  line treatments and assist in clinical trial enrollment. | Low |
| --- | --- | --- | --- | --- | --- | --- | --- | --- | --- | --- | --- | --- | --- | --- | --- |
| Filippo Pietrantonio et al. | Nomogram to  predict the  outcomes of patients with microsatellite  instability-high  metastatic colorectal cancer receiving  immune checkpoint inhibitors(111) | 2021 | Italy | The aim of the study was to develop and externally  validate a nomogram to  estimate progression-free  survival (PFS) and event-free probability (EFP) in patients with microsatellite instability- high (MSI-high) metastatic  colorectal cancer (mCRC) receiving immune  checkpoint inhibitors (ICIs) | The population included in  the study consisted of  patients with microsatellite  instability-high (MSI-high)  metastatic colorectal cancer  (mCRC) who were receiving  immune checkpoint  inhibitors (ICIs). Specifically,  the development set  included 320 patients  treated at 26 institutions  between 2006 and 2016,  and the validation sets  comprised 295 Italian  patients and 172 Korean  patients | Colorectal cancer | The random forest was used to develop the model by  combining decision trees built on subsampling the dataset. The model was  validated using internal  validation with 10-fold cross- validation. | The model was internally validated using 10-fold  cross-validation. The best models were combined to  create an ensemble model  through logistic regression of predicted probabilities.  Discrimination was assessed with AUC, and calibration  was evaluated using the  Hosmer-Lemeshow test and isotonic regression | Internal validation | The model used for  validation in the article was a Cox regression model | Age  Sex  Primary tumor sidedness Primary tumor resection Mucinous versus non-  mucinous histotype  RAS/BRAF mutational status  Synchronous versus  metachronous metastases Number of metastatic sites Presence of peritoneal  metastases  Lung metastases  Liver metastases  Bone metastases  Brain metastases  Lymph nodal metastases  Prior adjuvant treatment  Prior systemic treatment for  metastatic disease  Number of prior treatment  lines for metastatic disease ICI regimen (anti-PD-(L)1  monotherapy vs anti-PD-1 + anti-CTLA-4 combination)  ICI line  Eastern Cooperative  Oncology Group  Performance Status (ECOG PS) at the time of ICI  treatment start  Baseline lactate  dehydrogenase  Baseline platelet (PLT) count (× 103/mm3)  Baseline neutrophil-to-  lymphocyte ratio (NLR) | The PFS and EFP were  estimated using a cure  model fitted on a developing set of 163 patients and  validated on a set of 146 patients with MSI-high  mCRC  receiving anti-programmed death (ligand)1 (PD-(  L)1) ±  anticytotoxic T-lymphocyte  antigen 4 (CTLA-4) agents. A  total of 23 putative  prognostic factors were chosen and  then selected using a  random survival forest (RSF). The  model performance in  estimating PFS probability was  evaluated by assessing  calibration (internally —  developing  set and externally —  validating set) and  quantifying the  discriminative ability (Harrell C index) | RFS selected five variables:  ICI type (anti-PD-  (  L)1  monotherapy vs anti-CTLA- 4 combo), ECOG PS (0 vs  >0),  neutrophil-to-  lymphocyte  ratio (≤3 vs >3), platelet  count,  and prior treatment lines. As both in the developing and validation series most PFS  events occurred within 12  months, this was chosen as cut-point  for PFS prediction. The  combination of the selected variables allowed estimation of  the 12-month  PFS (focused on patients with low chance of  being cured) and the EFP  (focused on patients likely to be  event-free  at a certain point of their follow-up).  ICI type was  significantly associated with disease control, as patients receiving the anti-CTLA-  4-combination  experienced the best  outcomes. The calibration of PFS predictions was good  both  in the developing and  validating sets. The median value of  the EFP (46%) allowed  segregation of two  prognostic groups  in both the developing (PFS HR=3.73, 95% CI 2.25 to  6.18;  p<0.0001) and validating  (PFS HR=1.86, 95% CI 1.07 to  3.23; p=0.0269) sets. | The study concluded that a nomogram based on five  easily assessable clinical  variables was developed to  estimate 12-month  progression-free survival  (PFS) and the event-free  probability (EFP) of patients with MSI-high metastatic  colorectal cancer (mCRC) receiving immune  checkpoint inhibitors (ICIs). Prospective validation of the nomogram is required to  assess its discriminative performance in selected patient subgroups | Low |
| Inese Polaka et al. | Modular Point-of-  Care Breath Analyzer and Shape  Taxonomy-Based  Machine Learning for Gastric Cancer  Detection(112) | 2022 | Latvia | The aim of the study was to  report the diagnostic  performance of a modular  point-of-care breath analyzer  using gold nanoparticle  (GNP) and metal oxide  (MOX) semiconductor  sensors for detecting and  identifying gastric cancer in an online mode without  additional breath collection procedures or laboratory  settings. | The population of this study  included patients who were  at least 18 years old and  able to undergo a breath  exam. Patients with  morphologically confirmed  gastric adenocarcinoma  were included prior to their  gastric surgery, and a control  group of patients without  gastric cancer was included  prior to upper endoscopy | Gastric cancer | Random Forest was used as  a classifier in this study to achieve the second-best  results among the models tested. It was used to  evaluate the diagnostic  performance of the breath analyzer for gastric cancer detection by comparing  various feature sets and combinations | The model development  involved using the Random Forest classifier to identify relevant features and  combinations for detecting  gastric cancer. The  validation type used in the  study was internal validation. | Internal validation | The Random Forest model was used for validation,  specifically as a classifier in this study to evaluate the  diagnostic performance of the breath analyzer for  gastric cancer detection by comparing various feature sets and combinations | The factors used for  prognostication included  age, sex, primary tumor  sidedness, primary tumor  resection, mucinous versus non-mucinous histotype,  RAS/BRAF mutational  status, synchronous versus metachronous metastases, number of metastatic sites, presence of peritoneal  metastases, lung  metastases, liver  metastases, bone  metastases, brain  metastases, lymph nodal metastases, prior adjuvant treatment, prior systemic treatment for metastatic  disease, number of prior treatment lines for  metastatic disease, ICI  regimen (anti-PD-(L)1  monotherapy vs anti-PD-1 + anti-CTLA-4 combination),  ICI line, Eastern Cooperative Oncology Group  Performance Status (ECOG PS) at the time of ICI  treatment start, baseline  lactate dehydrogenase,  baseline platelet (PLT) count, and baseline neutrophil-to- lymphocyte ratio (NLR) | The method of this study  involved the preprocessing of sensor response curves, clustering of measurements, and feature extraction for  classification model training. The preprocessing included normalizing readings and  removing outliers.  Hierarchical clustering was  used to group similar curves, and these clusters were  used as features for  classification | The outcome of the study showed that the Naïve  Bayes classifier achieved the highest overall accuracy of  77.81% with a sensitivity  range of 46.9% to 66.54%  and specificity range of  83.64% to 85.27%. The  Random Forest classifier  achieved an overall accuracy ranging from 69.89% to  75.01%, sensitivity from  43.23% to 48.79%, and  specificity from 82.78% to  90.12%. The Support Vector Machines (SVM) classifier  showed an overall accuracy ranging from 72.75% to  75.1%, sensitivity from  40.86% to 61.05%, and  specificity from 80.72% to 91.14% | The proposed device allows the breath to be analyzed in any location, thus providing more flexibility than  laboratory-based  approaches. It uses a  combination of GNP and metal  oxide sensors, and provides good and stable accuracy. Paired with the suggested  data  analysis methods, it can  provide a quick and accurate technique for the detection  of gastric  cancer-specific breath, and  potentially for other  applications, e.g., the  detection of other  cancers, the monitoring of  the course of diseases, and population-based screening. | Low |
| Jacobo Porto-  Álvarez et al. | CT-Based Radiomics to Predict KRAS  Mutation in CRC Patients Using a Machine Learning Algorithm: A  Retrospective Study(113) | 2023 | Spain | The aim of the study is to  evaluate the potential of a novel therapeutic approach using a combination of  specific drugs to treat a  particular disease, focusing on the mechanisms of  action, efficacy, and safety of the treatment. | The population consisted of  332 patients diagnosed with  CRC, and the study utilized  retrospective data collected  from these individuals | Colorectal cancer | The study used Random  Forest as one of several  machine learning algorithms to predict the presence of  KRAS mutation in colorectal cancer patients based on  CT-based radiomics  features. The Random Forest  classifier was part of a  comparative analysis  involving other classifiers like AdaBoost, neural networks, and decision trees. | In the study "CT-Based  Radiomics to Predict KRAS Mutation in CRC Patients  Using a Machine Learning Algorithm," the model  development and validation process involved using  various machine learning  classifiers, including random forest, to predict the  presence of KRAS mutation in colorectal cancer (CRC) patients.  The validation of the model was conducted internally. The study used a  retrospective dataset of 56 CRC patients from the  Hospital of Santiago de  Compostela, Spain.  Radiomics features were  extracted from CT images,  and the models were trained and tested on this dataset to evaluate their performance. The study did not involve an external validation dataset | Internal validation | The internal validation was performed using cross-  validation techniques,  ensuring the robustness of the model within the same dataset used for model  training. This approach is typical in initial model  development phases to  check for consistency and reliability before applying external validation on  independent datasets. | Radiomic Features from CT  Images: These features are  extracted from pre-treatment contrast-enhanced CT scans and include texture patterns that may correlate with the  KRAS mutation status.  Machine Learning  Classifiers: Various  algorithms such as  AdaBoost, neural networks, decision trees, support  vector machines, and  random forests were used to classify patients based on  their KRAS mutation status.  Clinical Data: This includes information like tumor  location, presence of hepatic or pulmonary metastases,  tumor stage, and  differentiation. These clinical  parameters are combined with radiomic data to  improve prognostication accuracy. | The method of the study  involved a retrospective  analysis using contrast-  enhanced CT scans of  colorectal cancer (CRC)  patients. Radiomic features were extracted from these  scans, and various machine learning algorithms,  including AdaBoost, neural networks, decision trees,  support vector machines, and random forests, were employed to classify KRAS mutation status. The study combined radiomic data  with clinical information to  improve the prediction  accuracy of KRAS mutations in CRC patients | The outcome of the study demonstrated that the CT- based radiomics approach combined with machine  learning algorithms  effectively predicted KRAS mutation status in colorectal cancer (CRC) patients. The study found that specific  radiomic features from pre- treatment contrast-  enhanced CT scans, when analyzed with classifiers  such as AdaBoost and  neural networks, achieved high predictive accuracy.  This approach showed  potential in enhancing  personalized treatment  planning and improving  prognosis by identifying  KRAS mutation status non- invasively | The conclusion of the study was that CT-based  radiomics, combined with  machine learning algorithms, is a feasible and effective  method for predicting KRAS mutation status in colorectal cancer (CRC) patients. This non-invasive approach has the potential to enhance  personalized treatment  planning and improve  patient outcomes by  accurately identifying KRAS mutations | Moderate |
| Mohamad Amin  Pourhoseingholi et al. | Comparison of basic and ensemble data  mining methods in  predicting 5-year  survival of colorectal cancer patients(114) | 2017 | Iran | The aim of the study was to compare the efficiency of  prediction models based on multiple basic and ensemble data mining methods for  predicting 5-year survival of colorectal cancer (CRC)  patients | The population in this study  consisted of 395 colorectal  cancer (CRC) patients after  preprocessing and balancing  the data. Initially, there were  1127 records, but after  excluding irrelevant and  incomplete records, 261  remained. The Synthetic  Minority Oversampling  Technique (SMOTE) was  used to address class  imbalance, resulting in 395  records with approximately  equal numbers of survival  and non-survival patients | Colorectal cancer | The Random Forest (RF)  model in this study was used as a base classifier within  the ensemble voting  method. RF was  implemented specifically for decision trees, and it  contributed to the ensemble model's high predictive  power for the 5-year survival prediction of colorectal  cancer (CRC) patients. | Model Development  Data Preparation: The initial dataset comprised 21  variables and 261 records, which was balanced using the Synthetic Minority  Oversampling Technique  (SMOTE) to address class imbalance, resulting in 395 records with a nearly equal distribution of survival and non-survival instances.  Feature Selection: Both filter and wrapper methods were used for feature selection,  with the wrapper method providing better  classification results.  Ultimately, 16 features were  selected for model  construction.  Model Construction: Several  supervised classification methods from the WEKA toolkit were employed,  including:  Basic classifiers: C4.5, SVM, Naive Bayes, Bayesian  Network, ADTree, Radial  Basis Function, REPTree, KNN, and Random Forest.  Ensemble classifiers:  Bagging (with C4.5,  REPTree, Naive Bayes,  ADTree, Radial Basis  Function, SVM, Bayesian Network, KNN as base  learners) and Voting (using SVM, C4.5, Random Forest,  Bayesian Network, and  Naive Bayes as base  learners).  A total of 18 models were  developed: 9 basic individual classifiers, 8 ensemble  bagging models, and 1  ensemble voting model | Internal validation | The validation of the models  was done using internal  validation, specifically  through a stratified 10-fold cross-validation method.  This approach helps in  avoiding overfitting by  splitting the dataset into 10 stratified segments and  iterating 10 times, each time using one segment for  testing and the remaining segments for training | Age at Diagnosis (Dx)  Gender  Marital Status at Dx  Ethnicity  BMI  Hypertension  Diabetes Mellitus  Familial History of Cancer Personal History of Cancer  Bowel Obstruction  Bowel Perforation  Site (tumor location)  Histological Type  Tumor Size  Tumor Grade  Tumor Extension  Regional Lymph Node  Metastasis  Distant Metastasis  Tumor Stage  Inflammatory Bowel Disease (IBD)  Treatment Methods | The study used a  retrospective analysis with data from 1127 colorectal  cancer patients. After  preprocessing and feature selection, 16 prognostic  factors were identified. The data was balanced using  SMOTE, resulting in 395 records. Various  classification models,  including basic and  ensemble methods, were developed and validated using stratified 10-fold  cross-validation and  evaluated with AUC metrics | Model Performance: All  developed models had high predictive performance, with Area Under the ROC Curve (AUC) values exceeding  0.90.  Best Model: The ensemble voting model achieved the highest AUC of 0.96.  Ensemble Models: Ensemble methods generally  outperformed individual classifiers. | The conclusion of the study was that all developed  models achieved high  classification performance, with ensemble models  generally outperforming individual classifiers. The  ensemble voting model  achieved the highest AUC of 0.96. However, the ensemble voting method did not  significantly improve the  predictive performance over Random Forest (RF) and  Bayesian Network (BN)  classifiers. The study  suggested that RF and BN  are robust and powerful  machine learning techniques for predicting survival in  colorectal cancer patients, and these methods are also easily interpretable, which is important for clinical use | Moderate |
| Wei-Xiang Qi et al. | A machine learning approach using 18F-  FDG PET and  enhanced CT scan- based radiomics  combined with  clinical model to  predict pathological complete response in ESCC patients  after neoadjuvant  chemoradiotherapy and anti-PD-1  inhibitors(115) | 2024 | China | The aim of the study was to  evaluate the value of an integrated multimodal  radiomics approach  combined with machine learning to predict  pathological complete  response (pCR) in  esophageal squamous cell carcinoma (ESCC) patients treated with neoadjuvant  chemoradiotherapy (nCRT) and anti-PD-1 inhibitors | The population of the study  consisted of 126 patients  with histologically proven  esophageal squamous cell  carcinoma (ESCC), who  were treated with  neoadjuvant  chemoradiotherapy (nCRT)  and anti-PD-1 inhibitors.  These patients were  identified from a prospective  cohort and treated at Ruijin  Hospital, Shanghai Jiao  Tong University School of  Medicine, between January  2019 and July 2023 | Oesophageal squamous cell carcinoma | The Random Forest model in  the study was used to  develop the prediction  model by constructing a  forest of multiple decision  trees. It involved randomly  selecting subsets of features and training data, then  aggregating the predictions  from these individual trees to make the final prediction.  The prediction was  determined by selecting the  label with the highest  frequency among the  constituent trees | Model Development:  Machine Learning  Algorithms Used: Support Vector Machine (SVM),  Random Forest (RF),  eXtreme Gradient Boosting  (XGB), and Logistic  Regression (LR).  Feature Extraction:  Radiomics features were extracted from 18F-FDG PET and enhanced CT  images.  Feature Selection: Employed variance threshold selection  and LASSO regression to reduce the initial 842  features to the most  significant ones for the  model.  Model Training: Utilized k- fold cross-validation for  training. Hyperparameters for each model were fine- tuned using this method. Validation:  Performance Metrics:  Accuracy, sensitivity,  specificity, and area under the curve (AUC) of the  receiver operating  characteristic (ROC) curve. Validation Cohorts: Thirteen  patients were assigned to  the test cohort, and fifty-two patients were used for  training.  Evaluation: Models were  evaluated using the test set. SVM performed best with  AUC values of 0.775 for PET, 0.710 for CT, and 0.722 for  clinical models. The  combination model using SVM with PET, CT, and  clinical features achieved the highest AUC of 0.852 in the test set | Internal validation | The validation of the model was internal, as the study  employed a 5-fold cross-  validation method on the  training data to determine the optimal parameters and assess the model's  performance | TNM Staging: Higher TNM stages indicated increased tumor burden.  Tumor Length: Longer tumor length was associated with increased tumor burden and lower probability of  pathological complete  response (pCR).  Time Interval from nCRT to  Surgery: Longer intervals  were associated with  improved pathological  response.  Baseline Blood Biomarkers:  Lymphocyte Count: Higher counts were associated with improved pathological  response.  Neutrophil-to-Lymphocyte  Ratio (NLR): Lower NLR was associated with better  outcomes.  Other Clinical Factors:  Body Mass Index (BMI)  Smoking Status | Design: Retrospective  analysis from prospective trials (NCT04435197,  NCT04513418,  NCT03990532).  Participants: 126 patients  with histologically proven  esophageal squamous cell carcinoma (ESCC) treated  with neoadjuvant  chemoradiotherapy (nCRT) and PD-1 inhibitors followed by esophagectomy at Ruijin Hospital.  Data Collection: Clinical and demographic data, surgical procedures, and imaging  data from 18F-FDG PET/CT and enhanced CT images. Feature Extraction:  Radiomics features from  PET and CT images,  selected via LASSO  regression.  Machine Learning Models: Support Vector Machine  (SVM), Random Forest (RF), eXtreme Gradient Boosting (XGB), and Logistic  Regression (LR).  Model Training and  Validation: Training on 52  patients, testing on 13  patients, using k-fold cross- validation and evaluating  performance via accuracy, sensitivity, speciﬁcity, and AUC. | Clinical information of 126 ESCC patients were  included  for analysis. Radiomics  features were extracted from 18F-FDG PET and enhanced  plan CT images. Four  machine learning algorithms, including SVM (Support  Vector Machine), Random Forest (RF), and eXtreme  Gradient Boosting (XGB)  and logistic regression (LR), were applied using k-fold  cross-validation to predict pCR after nCRT. The  predictive ability of the  models was assessed using receiver  operating characteristics (ROC) curve analysis. | Machine learning models utilizing radiomics features from 18FFDG  PET and enhanced plan CT exhibit promising  performance in predicting  pCR in ESCC after nCRT  and anti-PD-1 inhibitors. The fusion of features from  multiple modalities  radiomics and clinical  features enhances the better predictive performance  compared to using a single modality alone. | Moderate |

| Zhang Qi et al. | Prediction model of poorly differentiated colorectal cancer  (CRC) based on gut bacteria(116) | 2022 | China | The aim of the study was to  screen the characteristic intestinal microbiota of  poorly differentiated  intestinal cancer. | The study population  consisted of 65 patients with  moderately and poorly  differentiated colorectal  cancer | Colorectal cancer | Random forest was used to  develop the model by  constructing multiple  decision trees in a random manner and aggregating  their predictions. | The model development  utilized random forest by  constructing a forest of  multiple decision trees,  aggregating their predictions for final output. Internal  validation was performed  using k-fold cross-validation to assess model  performance | Internal validation | The model validation was internal, utilizing k-fold  cross-validation to assess the predictive ability of the models | The factors used for  prognostication in the study included specific bacterial taxa such as Megamonas  and Bifidobacterium, which were identified as important bacteria in the model for  colorectal cancer  differentiation | Fecal samples were  collected from 124 patients with moderately  differentiated CRC and 123  patients  with poorly differentiated  CRC, and the bacterial 16S rRNA V1-V4 region of the  fecal samples was  sequenced. Alpha  diversity analysis was  performed on fecal samples to assess the diversity and  abundance of flora. The RDP classifier  Bayesian algorithm was used to analyze the  community structure. Linear discriminant analysis and  Student’s t test  were used to screen the differences in flora. The  PICRUSt1 method was used  to predict the bacterial  function, and  six machine learning models, including logistic regression,  random forest, neural  network, support vector machine,  CatBoost and gradient  boosting decision tree, were used to construct a  prediction model for the poor differentiation  of colorectal cancer. | There was no significant  difference in fecal flora alpha diversity between  moderately and poorly  differentiated  colorectal cancer (P > 0.05). The bacteria that accounted for a large proportion of  patients with poorly  differentiated  and moderately  differentiated colorectal  cancer were Blautia,  Escherichia-Shigella,  Streptococcus,  Lactobacillus,  and Bacteroides. At the  genus level, there were nine bacteria with high  abundance in the poorly  differentiated group,  including Bifidobacterium,  norank f Oscillospiraceae, Eisenbergiella, etc. There  were six bacteria with high abundance  in the moderately  differentiated group,  including Megamonas,  Erysipelotrichaceae_UCG-00 3, Actinomyces, etc. The RF  model had the highest  prediction accuracy  (100.00% correct). The  bacteria that had the  greatest variable importance  in the model were  Pseudoramibacter,  Megamonas and  Bifidobacterium. | The degree of pathological differentiation of colorectal cancer was related to gut flora, and poorly  differentiated  colorectal cancer had some different bacterial flora, and intestinal bacteria can be  used as biomarkers for  predicting poorly  differentiated CRC. | Moderate |
| --- | --- | --- | --- | --- | --- | --- | --- | --- | --- | --- | --- | --- | --- | --- | --- |
| Li Qin et al. | Development and validation of machine learning models for postoperative  venous  thromboembolism  prediction in  colorectal cancer  inpatients: a  retrospective study(117) | 2023 | China | The aim of the study was to develop different binary  classification venous  thromboembolism (VTE)  predictive models for  surgically hospitalized  colorectal cancer (CRC)  patients using various  machine learning methods, and to compare the  performance of these  models with existing risk models | The study included a  population of 1,191 surgical  colorectal cancer patients  who met the eligibility criteria  during the study period | Colorectal cancer | The random forest model  was developed by selecting subsets of features and  training data randomly, and then aggregating the  predictions from multiple decision trees | Model development involved using random forest, logistic  regression, and other  machine learning algorithms to create predictive models. The models were validated  internally using 5-fold cross- validation | Internal validation | The type of validation used in the study was internal  validation through 5-fold cross-validation | The factors used for  prognostication in the study were based on two VTE risk models: the Caprini score  and the Khorana score.  These factors included age, cancer site,  prechemotherapy platelet  count, hemoglobin level,  prechemotherapy leukocyte count, BMI, history of VTE,  and other clinical parameters | Data were extracted from  4,914 patients with  colorectal cancer between August 2019 and August  2022, and 1,191 patients  who underwent surgery on the primary tumor site with curative intent were  included. The variables  analyzed included patient-  level factors, cancer-level  factors, and laboratory test results. Model training was conducted on 30% of the  dataset using a ten-fold  cross-validation method and model validation was  performed using the total dataset. The primary  outcome was VTE  occurrence in postoperative 30 days. Six ML algorithms, including logistic regression (LR), random forest (RF),  extreme gradient boosting  (XGBoost), weighted support vector machine (SVM), a  multilayer perception (MLP) network, and a long short- term memory (LSTM)  network, were applied for  model fitting. The model  evaluation was based on six indicators, including receiver operating characteristic  curve-area under the curve  (ROC-AUC), sensitivity  (SEN), specificity (SPE),  positive predictive value  (PPV), negative predictive  value (NPV), and Brier score. Two previous VTE models  (Caprini and Khorana) were used as the benchmarks | The incidence of  postoperative VTE was  10.8%. The top ten  significant predictors  included lymph node  metastasis, C-reactive  protein, tumor grade,  anemia, primary tumor  location, sex, age, D-dimer level, thrombin time, and  tumor stage. In our results,  the XGBoost model showed the best performance, with a ROC-AUC of 0.990, a SEN  of 96.9%, a SPE of 96.1% in training dataset and a ROC- AUC of 0.908, a SEN of  77.5%, a SPE of 93.7% in validation dataset. All ML  models outperformed the previously developed  models (Caprini and  Khorana). | This study developed  postoperative VTE predictive  models using six ML  algorithms. The XGBoost  VTE model might supply a complementary tool for  clinical VTE prophylaxis  decision-making and the  proposed risk factors could shed some light on VTE risk  stratification in CRC  patients. | Low |
| Binxu Qiu et al. | Application of machine learning techniques in real- world research to predict the risk of liver metastasis in rectal cancer(118) | 2022 | China | The aim of the study was to develop and validate a  clinical prediction model  using machine learning  algorithms to predict the risk  of liver metastasis in patients with rectal cancer | he study included 19,957  patients from the SEER  database and an external  validation set of 924 patients  from two centers in China | Rectal cancer | The random forest algorithm was used as one of the six  machine learning algorithms to predict liver metastasis in rectal cancer patients.  Patients from the SEER  database were randomly  partitioned into a training set and an internal test set, with the training set used to build the model and the internal  test set used for initial  evaluation | Model development involved using six machine learning  algorithms, including  random forest, to predict  liver metastasis in rectal  cancer patients. The SEER database patients were split into training and internal test sets, with k-fold cross-  validation and grid search used for parameter  optimization. The model was initially validated internally  and further validated  externally with patient data from two hospitals in China | Internal/External validation | The model was validated  using both internal and  external validation. Internal  validation was performed  using the internal test set  from the SEER database,  while external validation was conducted using a cohort  from two hospitals in China | The factors used for  prognostication in the study included age at diagnosis, gender, T-stage, N-stage,  CEA level, grade of  differentiation, and tumor size | We integrated two rectal cancer cohorts from  Surveillance,  Epidemiology, and End  Results (SEER) and Chinese multicenter hospitals  from 2010-2017. We also built and validated liver  metastasis prediction  models for rectal cancer  using six machine learning algorithms, including  random forest (RF), light  gradient boosting (LGBM), extreme gradient  boosting (XGB), multilayer  perceptron (MLP), logistic regression (LR), and  Knearest  neighbor (KNN). The models were evaluated by  combining several  metrics, such as the area under the curve (AUC),  accuracy score, sensitivity, specificity and F1 score.  Finally, we created a network  calculator using the  best model. | The study cohort consisted of 19,958 patients from the SEER database  and 924 patients from two hospitals in China. The AUC  values of the six  prediction models ranged from 0.70 to 0.95. The XGB model showed the best  predictive power, with the following metrics assessed in the internal test set:  AUC (0.918), accuracy  (0.884), sensitivity (0.721),  and specificity (0.787). The XGB model was assessed in the outer test set with the  following metrics: AUC  (0.926), accuracy (0.919),  sensitivity (0.740), and  specificity (0.765). The XGB algorithm also shows a good fit on the calibration decision curves for both the  internal test set and the  external validation set.  Finally, we constructed an online  web calculator using the XGB model to help  generalize the model and to assist  physicians in their decision- making better. | We successfully developed an XGB-based machine  learning  model to predict liver  metastasis from rectal  cancer, which was also  validated with a real-world  dataset. Finally, we  developed a web-based predictor to guide clinical diagnosis and treatment strategies better. | Low |
| S A Rahman et al. | Prediction of long- term survival after gastrectomy using random survival  Forests(119) | 2021 | United kingdom | The aim of the study was to derive and validate a  prognostic model for overall survival after surgery for  gastric adenocarcinoma using a large national  dataset | The study population  consisted of 2,931 patients  who underwent a  gastrectomy for  adenocarcinoma. The  demographics were 65.3%  male with a median age of  71 years at diagnosis | Adenocarcinoma of stomach | The model in the article was developed using a random survival forest (RSF)  methodology. The model was trained and validated  internally using  bootstrapping, with  calibration and  discrimination assessed  through metrics like the  time-dependent area under the receiver operator curve (tAUC) . | The model development  used a random survival  forest (RSF) approach,  incorporating 29 clinical and pathological variables.  Validation was internal, using  bootstrapping methods to  assess calibration and  discrimination, including  metrics like time-dependent area under the receiver  operator curve (tAUC) and the integrated Brier score. | Internal validation | The model used for  validation was a random survival forest (RSF),  validated internally using bootstrapping methods. | The factors used for  prognostication included age, cT stage, cN stage, WHO performance status, ASA grade, pT/ypT, total number of positive lymph nodes, grade of  differentiation, completeness  of resection (R0/R1), and neoadjuvant treatment  received. | National audit data from  England and Wales were  used to identify patients who underwent a potentially  curative gastrectomy  for adenocarcinoma of the stomach. A total of 2931  patients were included and 29 clinical and pathological variables were  considered for their impact on survival. A non-linear  random survival forest  methodology was then  trained and validated  internally  using bootstrapping with calibration and  discrimination (time-  dependent area under the receiver operator curve  (tAUC)) assessed | The median survival of the  cohort was 69 months, with a 5-year survival of 53.2 per cent. Ten variables were  found to  influence survival  significantly and were  included in the final model, with the most important  being lymph node positivity, pT stage  and achieving an R0  resection. Patient  characteristics including  ASA grade and age were  also influential. On validation the model  achieved excellent  performance with a 5-year  tAUC of 0.80 (95 per cent c.i.  0.78 to 0.82) and good  agreement between  observed and  predicted survival  probabilities. A wide spread of predictions for 3-year  (14.8–98.3 (i.q.r. 43.2–84.4) per cent) and 5-year (9.4– 96.1 (i.q.r.  31.7–73.8) per cent) survival were seen. | A prognostic model for  survival after a potentially  curative resection for gastric adenocarcinoma was  derived and  exhibited excellent  discrimination and  calibration of predictions. | Low |
| S A Rahman et al. | Machine learning to predict early recurrence after oesophageal cancer surgery(120) | 2020 | United kingdom | The aim of the study was to develop and validate a  clinically useful predictive  model for early recurrence in oesophageal  adenocarcinoma using  machine learning techniques | The study population  consisted of a large  multicentre cohort of  patients who underwent  oesophagectomy for  adenocarcinoma of the  oesophagus, specifically  including those who  received neoadjuvant  therapy. | Oesophageal  adenocarcinoma | The random forest was used to develop the model by  combining 1000 decision  trees created on random  subsets of the data set. The model's hyperparameters  were tuned using cross- validation. Validation was  internal, using 0.632  bootstrapping with 1000  resampled data sets, and an internal-external validation  procedure was also  employed | Model development used a random forest with 1000  decision trees, tuned by  cross-validation. Validation was internal, using 0.632  bootstrapping with 1000  resampled data sets and an internal-external validation procedure | Internal/External validation | The model used for  validation was an ensemble model combining elastic net regularized logistic  regression (ELR), random forest (RF), and extreme gradient boosting (XGB) | Number of positive lymph nodes  Lymphovascular invasion Age  Sex  Tumor site  Response to neoadjuvant therapy  ypT category  Total number of lymph  nodes examined  Completeness of resection (R0/R1)  Tumor grade  Neoadjuvant treatment  (NACT/NACRT) | Consecutive patients who  underwent oesophagectomy for adenocarcinoma and had  neoadjuvant  treatment in one Dutch and six UK oesophagogastric  units were analysed. Using clinical characteristics  and postoperative  histopathology, models were generated using elastic net regression (ELR) and the  machine learningmethods  random forest (RF) and  extreme gradient boosting  (XGB). Finally, a combined  (ensemble) model of these  was generated. The relative importance of factors to  outcome was calculated  as a percentage contribution to the model. | A total of 812 patients were included. The recurrence  rate at less than 1 year was 29⋅1 per  cent. All of the models  demonstrated good  discrimination. Internally validated areas under the  receiver  operating characteristic  (ROC) curve (AUCs) were  similar, with the ensemble  model performing best  (AUC 0⋅791 for ELR, 0⋅801 for RF, 0⋅804 for XGB, 0⋅805 for ensemble). Performance was similar when  internal–external  validationwas used  (validation across sites,  AUC0 ⋅804 for ensemble). In the final model,  the most important variables were number of positive  lymph nodes (25⋅7 per cent)  and lymphovascular  invasion (16⋅9 per cent) . | The model derived using  machine learning  approaches and an  international data set  provided excellent  performance in quantifying the risk of early recurrence after surgery, and will be  useful  in prognostication for  clinicians and patients. | Low |
| Sha Sa et al. | Development and validation of a preoperative prediction model for colorectal cancer T- staging based on  MDCT images and clinical information(121) | 2017 | China | The aim of this study was to develope and validate a  preoperative prediction  model for colorectal cancer T-staging based on MDCT images and clinical  information | The population of the study  included 611 patients with  colorectal cancer lesions  confirmed by pathology. The  average age was 60.8 ± 10.8  years, ranging from 28 to 93  years, with a male to female  ratio of 1.24:1. Tumors were  more common in the left  colon, with the rectum and  sigmoid colon being the  predilection sites | Colorectal cancer | The random forest algorithm  was used in this study to  establish a preoperative  staging model for colorectal cancer. The model was  developed by generating training and test samples through the bootstrap  resampling technique,  creating multiple decision trees, and combining the results through majority  voting | The model development  involved training the random forest algorithm on clinical, imaging, and pathological  data from 419 patients.  Validation was performed internally by testing the  model on a separate group of 192 patients from the  same cohort | Internal validation | The model used for  validation was a random forest algorithm-based prediction model for  preoperative colorectal  cancer T-staging | The factors used for  prognostication in the model were: age, gender, CEA  level, CA19-9 level, tumor location, tumor size,  intestinal wall thickness, wall deformity, contrast  enhancement rate,  enhancement homogeneity, blurred outer edge, fat  infiltration, and infiltration into surrounding tissue | The clinical, imaging and pathological data of 611  patients with colorectal  cancer (419 patients in the training group and 192  patients  in the validation group) were collected. A spearman  correlation analysis was used to  validate the relationship among these factors and pathological T-staging. A prediction  model was trained with the  random forest algorithm. T  staging of the patients in the validation group was  predicted by both prediction model and traditional  method. The  consistency, accuracy,  sensitivity, specificity and area under the curve (AUC) were  used to compare the efficacy of the two methods.  Conclusions: The newly  established comprehensive model can improve the  predictive efficiency of  preoperative colorectal  cancer T-staging. | The outcome showed that the model significantly  improved the accuracy of preoperative T-staging of colorectal cancer, with an accuracy of 86.98%  compared to 51.04% using traditional methods | The conclusion was that the newly established random  forest-based model  improved the predictive  efficiency of preoperative colorectal cancer T-staging | Moderate |
| Ramon M Salazar et al. | Random Forest Modeling of Acute Toxicity in Anal Cancer: Effects of Peritoneal Cavity Contouring  Approaches on  Model Performance(122) | 2024 | United states | The aim of the study was to  determine whether the  predictive ability of acute  gastrointestinal (GI) toxicity random forest models could be improved by using dose- volume metrics derived from  bowel bag contours  following different contouring approaches | The study population  consisted of 119 patients  who underwent preoperative  chemoradiation therapy for  rectal cancer | Rectal cancer | The random forest model in the article was used to  predict acute grade =3  gastrointestinal (GI) toxicity based on dose-volume  metrics and  clinicopathologic factors for patients treated with  definitive chemoradiation for  squamous cell carcinoma of the anus. The model used  three different types of  bowel bag segmentations:  physician-delineated,  autosegmented by a deep learning model (nnU-Net)  following Radiation Therapy Oncology Group (RTOG)  guidelines, and  autosegmented  encompassing the entire  bowel space. The model's performance was evaluated using repeated cross-  validation (100 iterations with a 50%/50% training/ test split) | The model development  involved using a random  forest machine learning  approach to predict acute grade =3 GI toxicity. Three types of random forest  models were constructed based on different bowel  bag segmentation  approaches. The models were validated internally using repeated cross-  validation (100 iterations with a 50%/50% training/ test split) | Internal validation | The validation of the model was internal, as it used  repeated cross-validation on  the same dataset of 246 patients, without any  mention of an external  dataset for validation in the provided text | The factors used for  prognostication in the model included dose-volume  metrics and  clinicopathologic factors  such as age, sex, smoking  history, human  immunodeficiency virus  status, tumor location and size, tumor stages, surgery before radiation therapy,  chemotherapy treatment, and prescription dose | The method of this study  involved using a random  forest machine learning  approach to predict acute  grade =3 GI toxicity from  dose-volume metrics and  clinicopathologic factors in  246 patients treated with  chemoradiation for anal  cancer. Three types of bowel bag segmentations were  used: physician-delineated, autosegmented by a deep learning model (nnU-Net)  following RTOG guidelines, and autosegmented  encompassing the entire  bowel space. The models' performance was evaluated using repeated cross-  validation (100 iterations with a 50%/50% training/ test split) | The outcome of the study  indicated that the random  forest models based on  nnU-Net autosegmentations following RTOG guidelines significantly outperformed  those based on physician- delineated contours.  Additionally, models that  autosegmented the entire bowel space showed even greater improvement in  performance. The  performance metrics for the best models were:  AUROCC: 0.87 ± 0.05  AUPRC: 0.70 ± 0.09  F1 score: 0.68 ± 0.09 | The conclusion of the study was that autosegmented  bowel bag contours,  particularly those  encompassing the entire bowel space, significantly improve the predictive  performance of random forest models for acute  grade =3 GI toxicity in anal  cancer patients compared to physician-delineated  contours | Moderate |

| Lin, X. et al. | MRI-based radiomics model for preoperative prediction of extramural venous invasion of rectal  Adenocarcinoma(123) | 2024 | china | To assess EMVI  preoperatively through  radiomics technology, and use different algorithms  combined with clinical  factors to establish a variety of models in order to make the most accurate  judgments before surgery. | A total of 212 patients with  rectal adenocarcinoma  between September 2012  and July 2019 | rectal adenocarcinoma | as a prediction model | model development | internal validation | Sensitivity, specificity,  positive predictive value (PPV), and negative  predictive value (NPV) | Extramural venous invasion (EMVI) | A total of 212 patients with rectal adenocarcinoma  between September 2012  and July 2019 were included and distributed to training  and validation datasets.  Radiomics features were  extracted from pretreatment T2-weighted images.  Different prediction models (clinical model, logistic  regression [LR], random  forest [RF], support vector machine [SVM], clinical-LR model, clinical-RF model, and clinical-SVM model)  were constructed on the  basis of radiomics features and clinical factors,  respectively. The area under  the curve (AUC) and  accuracy were used to  assess the predictive  efficacy of different models. Sensitivity, specificity,  positive predictive value (PPV), and negative  predictive value (NPV) were also calculated. | The AUC of the LR model,  RF model, and SVM model for the training dataset was 0.959 (95% confidence  interval [CI] = 0.932–0.986), 0.861 (95% CI = 0.800–  0.922), and 0.981 (95% CI = 0.963–0.998), respectively, and 0.846 (95% CI = 0.744– 0.948), 0.831 (95% CI =  0.724–0.937), and 0.861 (95% CI = 0.761–0.960), respectively, for the  validation dataset | The radiomics-based  prediction model is a  valuable tool in EMVI  detection and can assist  decision-making in clinical practice. | Low |
| --- | --- | --- | --- | --- | --- | --- | --- | --- | --- | --- | --- | --- | --- | --- | --- |
| Liu, Z. et al. | Construction of Immune Infiltration-  Related LncRNA  Signatures Based on Machine Learning for the Prognosis in  Colon Cancer(124) | 2023 | china | to investigate dysfunctional IRL and construct a risk  model for improving the  outcomes of patients.to  explore immune-related and can be used for immuno  therapy lncRNAs in colon cancer. | Nineteen immune cell types | Colon Cancer | to mine the optimal  combination of lncRNAs | model development | internal validation | validated this model by  RNA-seq data | immune-related lncRNAs (IRL) (immune infltra tion- related and prognostic  lncRNA) | Nineteen immune cell types were collected for identi | Three lncRNAs, CYB561D2, LINC00638, and DANCR, are identifed as optimal lncRNAs that are related to the  prognosis of colon cancer with the maximum  AUC=0.770 | In conclusion, we give an immune-related and  prognostic lncRNA signature by com | Low |
| Shafi, A. S. M. et al. | Detection of colon cancer based on  microarray dataset using machine  learning as a feature selection and  classification  techniques(125) | 2020 | Bangladesh | analyze and predicts colon cancer data employing a  machine learning approach and feature selection  technique based on a  random forest classifer. | 62 cases (tests) and 2000 genes (attrib | colon cancer | feature selection and  classification techniques | model validation | external validation | tenfold cross-validation | top 20-genes selected by the two feature selection meth | combining the “Mean  Decrease Accuracy” and “Mean Decrease Gini” as feature selection methods into a renowned classifer  namely Random  Forest, with the aim of  increasing the prediction model’s accuracy level. In addition, we have also  shown a comparative  model analysis with  selection of features and  model without selection of features | resulting in a weighted recall,  precision, and F1-score of 83.68%, 83.87%, and  83.68% respectively. The overall accuracy of this  model is 83.871% using all genes. | In this examination, we  assessed the utilization of  machine learning techniques for the order of classifcation of colon cancer prediction/  prognosis dependent on the variation in gene expression. We additionally examined to discover the dependability of the most signifcant gene  expression or patterns from a natural point of view. For this reason, we have  presented the results of our experiments with and  without feature selection  algorithm. We also  compared the attributes  identifers of top 33 selected genes with those obtained from 2000 genes. We  achieved the best predic | Moderate |
| Shah, M. S. et al. | Re-purposing 16S  rRNA gene  sequence data from within case paired  tumor biopsy and tumor-adjacent  biopsy or fecal  samples to identify  microbial markers for colorectal cancer(126) | 2018 | United States | 1) to evaluate the degree to which tumor-associated  microbial communities were consistent with one another across studies (vs. non-  affected tissues) 2) to impute mechanistic pathways  through which mucosal  markers might operate and  3) to determine the degree to which fecal and mucosal  microbial communities  overlap with one another | ten colorectal cancer  associated studies-  comprising 588 matched  tumor and tumor-adjacent  specimens (n = 294 pairs  from nine studies)  and 84 matched fecal and  tumor biopsy specimens (n =  42 pairs from four studies; | colorectal cancer | random forest (RF) classifier | model validation | internal validation | e internally cross-validated ten-fold times with five  repeats | 16S rRNA gene(Microbial profiles) | n/a | The tumor biopsy vs. fecal  classifier [area under curve  (AUC) = 82.5] was better  able to distinguish CRC fecal samples from tumor tissue  samples than tumor vs.  tumor adjacent biopsy  classifier (AUC = 64.3).  Again, given the  compositional overlap  between these niches, these classifiers relied on  differentially abundant  features rather than niche- specific distribution. | n/a | Low |
| Wang, H. et al. | Blood Biomarkers Panels for Screening of Colorectal Cancer and Adenoma on a  Machine Learning- Assisted Detection Platform(127) | 2023 | china | The study aims to construct a machine learning-assisted detection platform with  available multi-targets for CRC and colorectal  adenoma (CRA) screening. | 817 participants were  retained, consisting of 229  HCs, 186 AAs, 198 nAAs,  and 204 CRCs | Colorectal Cancer and  Adenoma | RF is used to identify  essential features that  improve the quality of  training data set in our study  for both classification  problems and regression problems. | model development | internal and external  validation | areas under the curve (AUC), sensitivity (SE), specificity  (SP), accuracy (ACC), and F1 score, | CHO, LDL, HDL, TP, ALB, A/ G - NEUT, NLR,  LMR, PLR, and SII- CEA and AFP | This was a retrospective  study that the blood test  data from 204 CRCs, 384  CRAs, and 229 healthy  controls was extracted. The classified models were  constructed with 4 machine learning (ML) algorithms  including support vector  machine (SVM), random  forest (RF), decision tree  (DT), and eXtreme Gradient  Boosting (XGB) based on the candidate biomarkers. The  importance index was used by SHapely Adaptive  exPlanations (SHAP)  analysis to identify the  dominant characteristics.  The performance of  classified models was  evaluated. The most  dominating features from the  proposed panel were  developed by logistic  regression (LR) for  identification CRC from  control | the RF model with 26  candidate biomarkers  provided the best predictive  parameters (AUC: .941,  sensitivity: .902, and  specificity: .912). | In our investigation, 4 ML models were constructed  and compared, leveraging routine clinical data to  predict CRA and CRC. Our results highlight the SVM  model, incorporating 26 characteristics  demonstrated the best  performance in both CRA and CRC prediction. In  addition, for the simplified CRC model, RF algorithm outperformed its  counterparts. Summing up the above, compared with other models, the RF  emerges as a potentially  non-invasive,  straightforward, and robust tool for cancer detection. | Low |
| Wei, F. Z. et al. | Differential Expression Analysis Revealing CLCA1 to Be a Prognostic and Diagnostic  Biomarker for  Colorectal Cancer(128) | 2020 | china | find specific diagnostic  markers | 10 public datasets from the  NCBI Gene Expression  Omnibus (NCBI-GEO)- 41  control tissues and 482 CRC  tissues | Colorectal cancer (CRC) | utilized the random forest method to validat and  develop | model development | n/a | R package “randomForest” to validate the prognostic  model-accuracy, rrror rate, sensitivity  and precision -The Human Protein Atlas and Kaplan- Meier Plotter database | CLCA1 | In this study, we utilized 10 public datasets from the  NCBI Gene Expression  Omnibus (NCBI-GEO)  database to identify a set of significantly differentially  expressed genes (DEGs)  between tumor and control samples and WGCNA  (Weighted Gene Co-  Expression Network  Analysis) to construct gene co-expression networks  incorporating the DEGs from The Cancer Genome Atlas  (TCGA) and then identify  genes shared between the GEO datasets and key  modules. Then, these genes  were screened via MCC to identify 20 hub genes. We utilized regression analyses to develop a prognostic  model and utilized the  random forest method to validate. | ROC curve analysis showed  that these 20 genes have high diagnostic value for  CRC: CLCA1 AUC= 0.959, TMIGD1 AUC= 0.998,  SLC30A10 AUC= 0.993,  MT1F AUC= 0.933, MT1M AUC= 0.975, MT1G AUC= 0.944, MT1H AUC= 0.947, MT1E AUC= 0.943,  GUCA2B AUC= 0.991,  GUCA2A AUC= 0.99,  SLC26A3 AUC= 0.989,  CLCA4 AUC= 0.984,  MS4A12 AUC= 0.978, SI  AUC= 0.94, SLC9A2 AUC= 0.959, GCG AUC= 0.992,  PYY AUC= 0.993, SST  AUC= 0.992, SLC4A4 AUC= 0.997, and SLC16A9 AUC= 0.903 | In summary, we determined that CLCA1 could be used  as a prognostic marker for  CRC and correlated with  immune infiltration. It may be a potential therapeutic target for CRC to improve the  prognosis of patients.  However, our work has some limitations. First, more work needs to be done on the  pathogenic immune  responses and gene  expression in CRC cells to  identify the mechanism  linking the immune response with the development of  CRC. Second, validation in GEO datasets is not ideal and pure bioinformatics  analysis cannot well prove the prognostic significance of CLCA1 in colorectal  cancer, in future research we will focus on large-scale  population for further  investigation. Furthermore,  basic research needs to be  done to verify our model and the regulatory mechanism in vitro and in vivo | Moderate |

| Wu, X. et al. | Finding gastric cancer related genes and clinical  biomarkers for  detection based on  gene-gene  interaction network(129) | 2016 | china | The objective of this study was to develop an effective computational method to  meaningfully interpret these GC-related genes and to  predict potential prognostic genes for clinical detection. | 36 genes related to the  pathogenesis and  susceptibility of gastric  cancer backed by data | gastric cancer | classification and  incremental feature selection | model development | external validation | ten-fold cross validation- Matthews’s Correlation  Coefficient (MCC). | gastric cancer related genes | We employed the shortest path algorithm and  permutation test to probe the genes that have re | RF algorithm (Random  Forest algorithm) predicted 886 GC-related genes from all the genes in the  Ensemble Database. In  addition to those genes  considered as positive  control, we also predicted 827 genes associated with GC. --- As a result, we  confirmed that 39 genes  show significantly different expression (See Fig. 2),  which suggests that these genes are really involved in gastric cancer and this  supporting result for our identification for gastric cancer related genes is effective and reliable. | By analyzing the features of known GC-related genes, we employed a systematic  method to predict gastric cancer related genes and novel prognostic genes for accurate clinical detection. | Low |
| --- | --- | --- | --- | --- | --- | --- | --- | --- | --- | --- | --- | --- | --- | --- | --- |
| Yao, D. et al. | Geometric complement heterogeneous information and  random forest for  predicting lncRNA- disease associations(130) | 2022 | china | In this work, we have  proposed a computational model for predicting  lncRNA-disease  associations based on  geometric complement  heterogeneous information and random forest | geometric complement heterogeneous information | colon and stomach cancer | The random forest method is  used in the GCHIRFLDA model for predicting  lncRNA-disease  associations. a random  forest classifier was trained  on  the constructed sample set to score potential lncRNA- disease associations | model development | internal validation | five-fold cross-validation- SVM and Xgboost | lncRNA | GCHIRFLDA (Geometric  Complement Heterogeneous Information and Random  Forest for predicting  lncRNA-Disease  Associations(geometric  complement heterogeneous information was used to  integrate lncRNA-miRNA interactions and miRNA- disease associations | AUC (area under the receiver operating characteristic  curve) is 0.9897 and the AUPR (area under  the precision-recall curve) is 0.7040  GPT-4o&Web Access | In this work, we proposed a geometric complement  heterogeneous information and random forest-based  approach for predicting  LDAs (named GCHIRFLDA). Firstly, the potential LDA  matrix is constructed by integrating the LMIs and MDAs  with the original LDA matrix. Then, the Jaccard similarity and  the Gaussian interaction  profile similarity of lncRNA and disease  are combined to represent features of lncRNA and  disease. Next,  a low-dimensional feature  space is extracted by using autoencoder. Finally, RF is  employed as the classifier to predict potential LDAs. In  conclusion, the AUC and AUPR  comparison with other LDA prediction models based on five fold cross-validation and the case studies show that  our model  has better LDA prediction performance.  Although the GCHIRFLDA  model has a good  performance,  it still has some limitations. Firstly, the lack of data  verified by  biological experimental is a big shortcoming for  computational  models. Secondly, randomly selecting the unknown  lncRNA disease pairs as  negative samples may  incorrectly classify potential  positive samples as negative samples, which may affect  the prediction performance.  Finally, only the  heterogeneous information of miRNAs is introduced in this work, and in the future, more biological information will be fused to improve the performance of the LDA  prediction model. | Moderate |
| Zhao, G. et al. | Droplet digital PCR- based circulating  microRNA detection serve as a promising diagnostic method  for gastric cancer(131) | 2018 | china | In this study, we explored  the use of random forest  model based learning for GC diagnosis, by using circu | training cohort of 147  participants and a validation  cohort of 28 participants | gastric cancer | established in the training stage.and analysis | model validation | internal validation | validation cohort | stable circulating  microRNA(miR-21, miR-93,  miR-106a and  miR-106b) | Based on the quantitative  droplet digital PCR (ddPCR),  four miRNAs (miR-21,  miR-93, miR-106a and  miR-106b) related to the  presence of GC were  identified in plasma from a training cohort of 147  participants and a validation cohort of 28 participants. | AUC value: 0.887 ROC: 0.809 | Overall, the present study  demonstrated that by using the ddPCR technique,  circulating miR-21, miR-93, miR-106a and miR-106b  could be used as diagnostic plasma biomarkers in gastric cancer patients | Low |
| Zhou, R. et al. | Immune cell infiltration as a  biomarker for the  diagnosis and  prognosis of stage I- III colon cancer(132) | 2019 | china | With the goal of improving  early diagnosis and  prognosis prediction in colon cancer, in the current study, we employed the algorithm “Cell type Identification By  Estimating Relative Subsets Of RNA Transcripts  (CIBERSORT)”, which has  been deemed to be the most accurate method available | 870 colon cancer patients and 70 normal controls | stage I–III colon cancer | used to establish  immunoscores for diagnosis and prognosis (Random  forest analysis and least  absolute shrinkage and  selection operator (LASSO) analysis were both applied to identify the most  important  immune cells that could be used to differentiate tumor and  normal tissues) | model development | internal validation | Kaplan–Meier method | Immune cell infiltration | We developed a novel  immune model based on  systematic assessments of the immune landscape  inferred from bulk tumor  transcriptomes of stage I–III colon cancer patients. The “Cell type Identification By Estimating Relative Subsets Of RNA Transcripts  (CIBERSORT)” algorithm  was used to estimate the  fraction of 22 immune cell  types from six microarray  public datasets. The random forest method and least  absolute shrinkage and  selection operator model  were then used to establish immunoscores for diagnosis and prognosis. | we constructed adiagnostic model, designated the  diagnostic immune risk  score (dIRS), that showed high specificity and  sensitivity in both the  training [area under the  curve (AUC)=0.98, p | In conclusion, our study  demonstrates the utility of con | Low |

Supplementary table: Extracted data from the included studies in the review. Articles in this table are cited in the main manuscript.

.
